# Bone mineral density and risk of type 2 diabetes and coronary heart disease: A Mendelian randomization study

**DOI:** 10.12688/wellcomeopenres.12288.1

**Published:** 2017-08-22

**Authors:** Wei Gan, Robert J. Clarke, Anubha Mahajan, Benard Kulohoma, Hidetoshi Kitajima, Neil R. Robertson, N. William Rayner, Robin G. Walters, Michael V. Holmes, Zhengming Chen, Mark I. McCarthy

**Affiliations:** 1 Wellcome Trust Centre for Human Genetics, University of Oxford, Oxford, OX3 7BN, UK; 2 Oxford Centre for Diabetes, Endocrinology, and Metabolism, University of Oxford, Oxford, OX3 7LE, UK; 3 Big Data Institute, University of Oxford, Oxford, OX3 7FZ, UK; 4 Clinical Trial Service Unit and Epidemiological Studies Unit, Nuffield Department of Population Health, University of Oxford, Oxford, OX3 7LF, UK; 5 Centre for Biotechnology and Bioinformatics, University of Nairobi, Nairobi, Kenya; 6 National Institute of Health Research Oxford Biomedical Research Centre, Oxford, OX3 7LE, UK; 7 Medical Research Council Population Health Research Unit, University of Oxford, Oxford, OX3 7LF, UK

**Keywords:** Bone mineral density, Type 2 diabetes, Cardiovascular disease, Coronary artery disease, insulin resistance, Mendelian randomization, UK biobank, Causality

## Abstract

**Background:** Observational studies have demonstrated that increased bone mineral density is associated with a higher risk of type 2 diabetes (T2D), but the relationship with risk of coronary heart disease (CHD) is less clear. Moreover, substantial uncertainty remains about the causal relevance of increased bone mineral density for T2D and CHD, which can be assessed by Mendelian randomisation studies.

**Methods:** We identified 235 independent single nucleotide polymorphisms (SNPs) associated at *p*<5×10 ^-8^ with estimated heel bone mineral density (eBMD) in 116,501 individuals from the UK Biobank study, accounting for 13.9% of eBMD variance. For each eBMD-associated SNP, we extracted effect estimates from the largest available GWAS studies for T2D (DIAGRAM: n=26,676 T2D cases and 132,532 controls) and CHD (CARDIoGRAMplusC4D: n=60,801 CHD cases and 123,504 controls). A two-sample design using several Mendelian randomization approaches was used to investigate the causal relevance of eBMD for risk of T2D and CHD. In addition, we explored the relationship of eBMD, instrumented by the 235 SNPs, on 12 cardiovascular and metabolic risk factors. Finally, we conducted Mendelian randomization analysis in the reverse direction to investigate reverse causality.

**Results:** Each one standard deviation increase in genetically instrumented eBMD (equivalent to 0.14 g/cm ^2^) was associated with an 8% higher risk of T2D (odds ratio [OR] 1.08; 95% confidence interval [CI]: 1.02 to 1.14; *p*=0.012) and 5% higher risk of CHD (OR 1.05; 95%CI: 1.00 to 1.10; *p*=0.034). Consistent results were obtained in sensitivity analyses using several different Mendelian randomization approaches. Equivalent increases in eBMD were also associated with lower plasma levels of HDL-cholesterol and increased insulin resistance. Mendelian randomization in the reverse direction using 94 T2D SNPs or 52 CHD SNPs showed no evidence of reverse causality with eBMD.

**Conclusions: **These findings suggest a causal relationship between elevated bone mineral density with risks of both T2D and CHD.

## Introduction

The worldwide prevalence of type 2 diabetes (T2D) has increased dramatically over the last few decades, with an estimated 400 million affected individuals in 2015 ^1^. The long-term consequences of T2D account for a substantial proportion of premature death and disability. Increased adiposity, sedentary lifestyle and poor diet are the chief determinants of the global epidemic of T2D and coronary heart disease (CHD), but other risk factors that are amenable to lifestyle changes or drug treatment remain to be identified ^2– 6^.

Observational studies have reported associations between bone mineral density (BMD) and risk of T2D, with studies showing that BMD is higher in individuals with diabetes than in individuals free from diabetes ^7– 11^. Paradoxically, while individuals with T2D have higher BMD, as measured by dual energy X-ray absorptiometry (DXA), they also have higher risk of fracture, compared with non-diabetic individuals ^9, 12^. However, the results of previous studies of the associations of BMD and risk of CHD have been conflicting, with some reporting positive, negative or null associations ^13– 16^. Moreover, the causal relevance of BMD for both T2D and CHD cannot be fully addressed by traditional observational studies, which are typically constrained by residual confounding and reverse causality bias.

Bone not only serves as a scaffold for other organs, but is also an endocrine organ involved in the regulation of glucose and energy metabolism ^17^. The biological process of bone remodelling, by which bone tissue is constantly broken down by osteoclasts and regenerated by osteoblasts, is regulated by several hormones including leptin, adiponectin, glucagon-like peptides 1 and 2, and osteoblast-derived osteocalcin ^18^. Such hormones may also influence risk of cardiometabolic diseases, prompting interest in assessing associations of bone density with T2D and CHD.

Bone structure *in vivo* has largely been assessed using DXA method to measure BMD. Over the past decade, quantitative ultrasound (QUS) methods have been widely used to assess bone quality in large-scale studies, such as the UK Biobank study ^19^, as QUS measurement is quick, easy to use, portable and less expensive than DXA. QUS provides information not only on bone density (correlation coefficients with central DXA BMD [i.e. lumbar spine and hip BMD]: 0.4–0.8), but also provides information on the structure and elastic properties of bone ^20^. Previous genetic studies of heel bone density assessed by QUS reported evidence for some genetic loci common to heel QUS measures and central DXA BMD, but also identified additional genetic variants associated with bone structure that had not previously shown association with central DXA ^21^.

The causal relationship between estimated heel BMD (eBMD) and cardiometabolic traits (particularly T2D and CHD) can be assessed using Mendelian randomisation (MR) approaches. In contrast to conventional epidemiological methods, MR can facilitate robust causal inference by using genetic variants as instruments ^22^. Since genetic variants are randomly allocated at conception, their associations with exposures of interest are not susceptible to reverse causation and should be unaffected by confounding. Recent developments of MR (including two-sample approaches, such as inverse-variance weighted [IVW] MR, MR-Egger, weighted-median MR, weighted mode MR and MR-PRESSO), together with increasing availability of summary GWAS data facilitate investigations of causality and permit a detailed assessment of reliability by testing potential unbalanced horizontal pleiotropic (i.e. when a genetic association with the outcome is mediated via different pathways than the exposure of interest; for further details see Box 1 in Holmes *et al.* ^6^).

**Figure 1.**
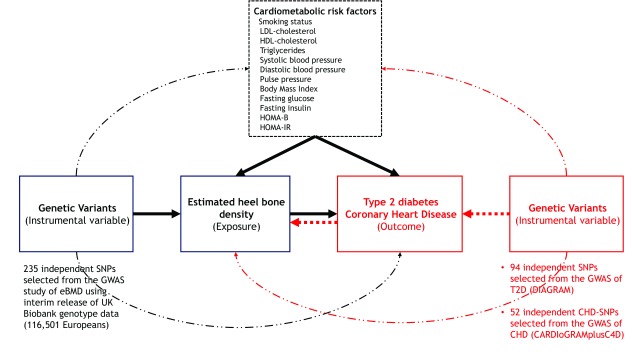
Framework for the Mendelian randomization analysis of estimated heel bone mineral density with risk of type 2 diabetes and coronary heart disease. We used 235 SNPs identified from the GWAS of estimated bone mineral density (eBMD) in UKB as genetic instruments for eBMD and applied them to data from DIAGRAM (T2D) and CARDIoGRAM (CHD) in order to characterise the causal relationships of eBMD with these diseases. We additionally analysed the association of the 235 SNP instrument with 12 cardiometabolic risk factors which may be potential confounders and/or mediators of the eBMD to disease relationship. To assess whether T2D or CHD impact on eBMD, we conducted reverse MR using 94 SNPs identified in published GWAS for T2D, and 52 SNPs identified in published GWAS for CHD. Details of the datasets used are provided in Table 1.

The aims of the present study were: (i) to conduct a GWAS of eBMD in UK Biobank, in which >97% of the participants have eBMD; and (ii) to examine the relationships of eBMD-associated SNPs with T2D and CHD through MR analyses using data from large GWAS consortia in order to ascertain whether there is genetic support for the hypothesis that higher eBMD causes higher risk of T2D and CHD ( Figure 1).

**Table 1.** Characteristics of the study population in UK Biobank and other publically available datasets.

Study	Variable	Descriptive statistics	Web source
**UK Biobank**			www.ukbiobank.ac.uk
	Self-reported European participants, n (female %)	457,395 (54.1%)	
	Age (years), Mean (SD)	56.8 (8.0)	
	Weight (kg), Mean (SD)	78.2 (16.0)	
	Height (cm), Mean (SD)	168.6 (9.3)	
	Systolic blood pressure (mmHg), Mean (SD)	141.9 (20.9)	
	Diastolic blood pressure (mmHg), Mean (SD)	83.4 (11.5)	
	eBMD (g/cm ^2^) , Mean (SD)	0.54 (0.14)	
	Self-reported diabetes, n	22,186	
	Self-reported coronary heart disease, n	26,503	
**CARDIoGRAMplusC4D** **Consortium**			www.cardiogramplusc4d.org
	CHD cases/controls, n	60,801/123,504	
**DIAGRAM Consortium**			www.diagram-consortium.org
	T2D cases/controls, n	26,676/132,532	
**GIANT Consortium**			http://portals.broadinstitute.org/ collaboration/giant/index.php/ Main_Page
	Body mass index (kg/m ^2^), n	322,154	
**GLGC Consortium**			www.lipidgenetics.org
	LDL-cholesterol (mmol/L), n	173,058	
	HDL-cholesterol (mmol/L), n	187,137	
	Triglycerides (mmol/L), n	177,827	
**MAGIC Consortium**			www.magicinvestigators.org
	ln-Fasting insulin (pmol/L), n	108,557	
	Fasting glucose (mmol/L), n	133,010	
	HOMA-B, n	46,186	
	HOMA-IR, n	46,186	
**Tobacco and Genetics** **Consortium**			www.med.unc.edu/pgc
	Smoking status (ever vs. never users)	74,053	

## Methods

### Study population

The study design consisted of two components. First, a genome wide association study (GWAS) for eBMD was performed to identify SNPs associated with estimated heel bone mineral density (eBMD) in European populations, using data from the interim release of ~150,000 UK biobank participants ( http://www.ukbiobank.ac.uk), described in detail elsewhere ^19^. The UK biobank is a prospective study of 502,655 community-dwelling people aged between 37 and 73 years recruited in the United Kingdom between 2006 and 2010. Self-reported baseline data were collected by questionnaire, and anthropometric assessments were performed. At baseline, 457,395 participants self-reported that they were European, and among these, there were 22,186 self-reported diabetes cases and 26,503 self-reported CHD cases. Over 97% of the participants had at least one foot measured for BMD based on an ultrasound measurement of the calcaneus, using a Sahara Clinical Bone Sonometer (Hologic, Inc., Bedford, USA). For the present study, 116,501 individuals of European ancestry with GWAS data and heel ultrasound measurements were available after quality control. Genotyping, imputation and quality control procedures are provided by UK Biobank ( http://biobank.ctsu.ox.ac.uk/). For this study, we included only variants with an imputation r ^2^ ≥ 0.4, MAF ≥ 0.001, missingness <0.1 and with a Hardy–Weinberg equilibrium *p*>1×10 ^-6^. The GWAS study of eBMD in the present study identified 235 independent SNPs at 197 separate loci (defined as r ^2^ < 0.05 and +/-500 KB) associated with eBMD at *p*< 5×10 ^-8 ^ ( Supplementary Table 1 and Supplementary Table 2). The estimates of the association of the 235 SNPs with eBMD were used to construct a weighted eBMD allele score for two-sample MR analysis ( Supplementary Table 3).

For each eBMD-associated SNP, we retrieved GWAS summary statistics from the largest 1000 Genomes-based GWAS studies to date of both T2D (DIAGRAM: 26,676 T2D cases and 132,532 controls) ^23^ and CHD (CARDIoGRAMplusC4D: 60,801 CHD cases and 123,504 controls) ^24^, and other conventional cardiovascular risk factors, in populations of Europeans ancestry. Of the 235 eBMD-associated SNPs, three were not present in the T2D (DIAGRAM) or CHD (CARDIoGRAMplusC4D) GWAS consortia. Data on CHD were contributed by CARDIoGRAMplusC4D investigators and downloaded from www.cardiogramplusc4d.org. Details of the study populations included in the analysis are provided in Table 1.

### Statistical analysis

Logistic regression was used to estimate the traditional observational estimates of eBMD with prevalent diabetes and CHD using cross-sectional data from the UK Biobank with adjustment for age, age squared, sex, weight, height, research centre and smoking status. For genome-wide association, we used BOLT-LMM (Version 2.2) to perform linear mixed models, which adjusted for population structure and relatedness between individuals ^25^. For men and women separately, eBMD was regressed on age, age-squared, height, weight, genotyping array version and assessment centre, and the residuals were transformed by the rank-inverse standard normal function. The normalized residuals were subsequently pooled together (between men and women) for genome-wide association analyses. Conditional analyses were performed to identify the presence of multiple signals within the locus from the genotype-phenotype analyses.

Inverse-variance weighted (IVW) MR analyses were performed by regression of the SNP-outcome (T2D or CHD) associations on the SNP-eBMD associations. Sensitivity analyses were used to investigate the potential presence of directional (unbalanced horizontal) pleiotropic effects: (i) MR-Egger provides a statistical test for presence of pleiotropic effects due to aggregation of invalid genetic instruments, assuming absence of dose-response confounding of SNPs through pleiotropic pathways ^26^; (ii) weighted median MR should provide a valid causal effect estimate if more than 50% of the information arises from valid genetic instrumental variables ^27^; (iii) weighted mode MR produces robust causal effects when the largest number of similar individual-instrument causal effect estimates arise from valid instruments, even if the majority of instruments are invalid ^28^; (iv) MR-PRESSO detects the presence of variant effect sizes that are outliers and corrects pleiotropy via outlier removal ^29^. MR analysis was further applied to investigate the causal associations of eBMD on 12 established cardiovascular and metabolic risk factors (i.e. smoking status, LDL-cholesterol, HDL-cholesterol, triglycerides, systolic blood pressure, diastolic blood pressure, pulse pressure, body mass index, fasting glucose, fasting insulin, HOMA-B, HOMA-IR). As a positive control, one sample MR was employed to investigate the causal association of eBMD and risk of any fracture within the past 5 years using the individual-level data from UK Biobank after using 20-fold cross-validation to generate valid weights ^30^. Finally, to test whether genetic liability to T2D or CHD might be causally related to eBMD, we performed MR in the opposite direction (i.e., bidirectional MR), testing the effects of 94 T2D-associated and 52 CHD-associated SNPs on eBMD. MR analysis was performed using the TwoSampleMR package for R (version 3.2.2).

## Results

### Observational associations of eBMD with risk of T2D and CHD

**Figure 2.**
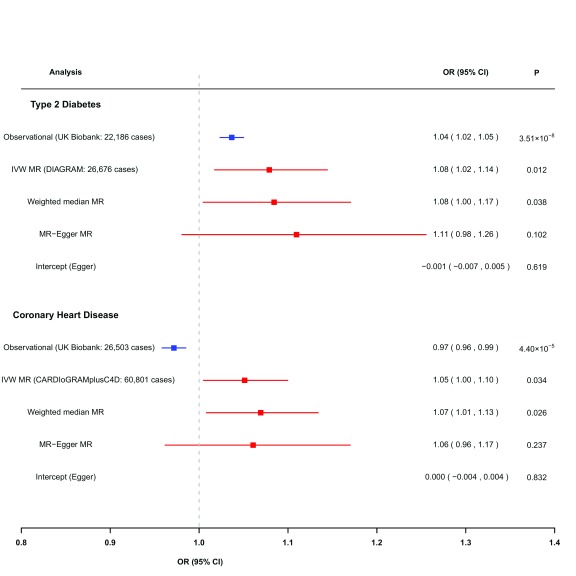
Comparison of observational (blue) and causal (derived from Mendelian randomization using 232 SNPs as genetic instruments; red) estimates for risk of type 2 diabetes and coronary heart disease, per 1-SD (equivalent to 0.14 g/cm ^2^) higher eBMD. Observational analyses are adjusted for age, age squared, sex, weight, height, research centre and smoking status. Mendelian randomization estimates are derived from two-sample analyses. IVW: inverse variance weighted.

Analysis of the observational association of eBMD and risks of diabetes in UK Biobank indicated that a one-SD higher eBMD (equivalent to 0.14 g/cm ^2^) was associated with a 4% higher risk of diabetes (odds ratio [OR] 1.04; 95% confidence interval [CI]: 1.02 to 1.05, *p*<0.001, Figure 2) and a 3% lower risk of CHD (OR 0.97; 95% CI: 0.96 to 0.99, *p*<0.001) after adjusting for age, age squared, sex, weight, height and research centre.

### Identification of SNPs associated with eBMD

**Figure 3.**
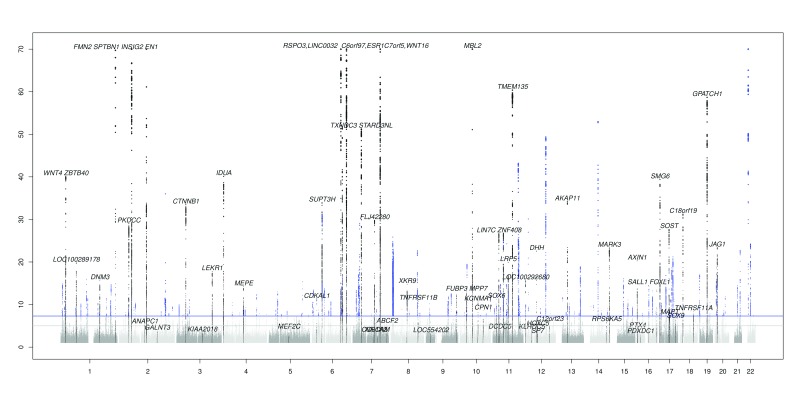
Manhattan plot of the results of GWAS of eBMD (Scale of -log10 ( *P* value) range from 0–70 only). Novel loci are highlighted in blue and known loci are in black and labelled with gene name.

The GWAS of eBMD in 116,501 individuals of European ancestry from UK Biobank in this study identified 235 conditionally independent association signals that reached genome-wide significance ( *p*< 5×10 ^-8^) at 197 loci with effect sizes ranging from 0.02 to 0.41 SDs per eBMD increasing allele ( Figure 3, Supplementary Table 1 and Supplementary Table 2, Supplementary Figure 1). For those 80 lead SNPs reported by previous GWAS studies of BMD in multiple skeletal sites measured by different methods (including DXA and QUS), 60 SNPs were replicated at *p*<0.05 and 40 SNPs reached genome-wide significance ( Supplementary Table 4). The proportion of variance of eBMD explained by all of the 235 SNPs was 15.9% using the method described by Shim *et al.* ^31^. The equivalent figure obtained using the weighted genetic risk score with 20-fold cross was 13.9%. The effect estimates of 30 DXA BMD (i.e. lumbar spine BMD and femoral neck BMD) related SNPs were highly correlated with the estimates on eBMD ( Supplementary Figure 2). There was no evidence of heterogeneity of the effect estimates for these SNPs after stratification by smoking status or array subtype, diminishing the possibility of confounding arising from the smoking-enriched subset of UKBB that was genotyped on a slightly earlier version of the UKBB axiom array ( Supplementary Table 5). The eBMD genetic risk score (GRS) was constructed using 235 SNPs by summing up the number of eBMD-increasing alleles for each SNP multiplied by their effect sizes derived from 20-fold cross validation analysis. The GRS was strongly associated with BMD measured by the DXA method in multiple skeletal sites, with the same direction of effect ( *p*<2.0×10 ^-7^) ( Supplementary Table 6).

### Associations with fracture

**Figure 4.**
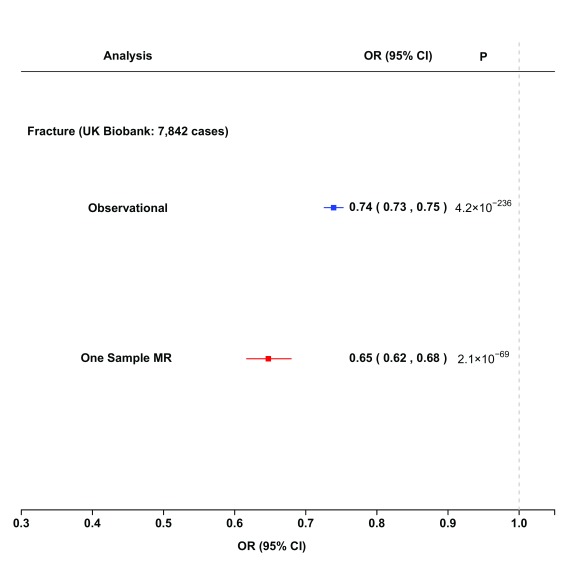
Comparison of observational (blue) and causal (derived from Mendelian randomization, red) estimates for fracture per 1-SD (equivalent to 0.14 g/cm ^2^) higher eBMD. Observational analyses are adjusted for age, age squared, sex, weight, height, research centre and smoking status. Mendelian randomization estimates are derived from one-sample analysis in the UK biobank with weights obtained from 20-fold cross-validation. IVW: inverse variance weighted.

Analysis using the GRS of 235 SNPs as a genetic instrument for eBMD with risk of fracture identified that a 1-SD higher eBMD was associated with over a 30% lower risk of fracture, which was consistent with the estimates derived from observational analyses (observational estimate: OR 0.74; 95% CI: 0.73 to 0.75, *p*=4.2×10 ^-236^ vs. genetic estimate: OR 0.65; 95% CI: 0.62 to 0.68 *p*=2.1×10 ^-69^) ( Figure 4).

### Associations with T2D and CHD

Using conventional IVW MR in 26,676 T2D cases and 132,532 controls in DIAGRAM consortium, a one-SD higher eBMD (equivalent to 0.14 g/cm ^2^) instrumented by 232 SNPs present in both of DIAGRAM and CARDIoGRAMplusC4D GWAS consortia was associated with an 8% (95%CI: 2% to 14%, *p*=0.012) higher risk of T2D and 5% (95%CI: 0% to 10%, *p*=0.034) higher risk of CHD ( Figure 2, Supplementary Figure 3 and Supplementary Figure 4). Sensitivity analyses using weighted median MR, MR-Egger, weighted mode MR and MR-PRESSO demonstrated consistent directions and similar effect estimates to IVW, and provided no evidence of unbalanced pleiotropy ( *p* for pleiotropy from MR-Egger ≥0.62) ( Figure 2, Supplementary Table 7). Restricting the SNPs included in the eBMD genetic instrument to those with more extreme *P*-values (i.e. choosing cut-offs for inclusion that were more extreme than the genome wide significance threshold), or those previously identified in prior BMD GWAS studies, produced consistent MR estimates for both T2D and CHD ( Supplementary Table 8– Supplementary Table 10). For example, using 53 SNPs associated with eBMD at p<5×10 ^-20^, explaining 9% of eBMD variance, a 1-SD genetically instrumented higher eBMD associated with higher risks of both T2D (OR 1.09; 95%CI: 1.02 to 1.17, *p*=0.020) and CHD (OR 1.08; 95%CI: 1.02 to 1.15, *p*=0.013).

For T2D, the MR effect size estimates were consistent with the estimates derived from traditional observational analyses ( *p* for heterogeneity between IVW MR and observational estimate=0.14). For CHD, the MR estimates were directionally opposite to those derived from the observational analysis ( *p* for heterogeneity between IVW MR and observational estimate =0.001 ( Figure 2).

### Associations with cardiovascular and metabolic risk factors

The 235 SNP eBMD genetic instrument was also used to assess the associations of eBMD with 12 established cardiovascular and metabolic risk factors. Genetically-instrumented higher eBMD was nominally associated with insulin resistance phenotypes, including higher levels of HOMA-IR (β= 0.02, 95% CI: 0.00 to 0.04, *p*=0.029) and lower plasma levels of HDL-cholesterol (β= -0.04, 95% CI: -0.07 to -0.01, *p*=0.008), but not with any of the other CHD risk factors ( Table 2). 

**Table 2.** Genetic associations for a 1-standard deviation higher eBMD with selected cardiovascular and metabolic risk factors.

Outcome	Estimate (95% CI) per 1-SD higher eBMD	*P*
Traits recognised to contribute to insulin resistance (units)	(odds ratio ^a^ or β ^b^)	
HDL-cholesterol (mmol/L)	-0.04 (-0.07, -0.01) ^b^	0.008
Triglycerides (mmol/L)	0.01 (-0.02, 0.04) ^b^	0.431
ln-Fasting insulin adjusted for BMI (pmol/L)	0.02 (-0.01, 0.05) ^b^	0.234
HOMA-IR	0.02 (0.00, 0.04) ^b^	0.029
Other traits (units)		
Smoking status (ever vs. never users)	0.99 (0.92, 1.06) ^a^	0.786
LDL-cholesterol (mmol/L)	-0.02 (-0.06, 0.02) ^b^	0.247
Systolic blood pressure (mmHg)	-0.54 (-2.97, 1.89) ^b^	0.662
Diastolic blood pressure (mmHg)	-0.96 (-2.33, 0.42) ^b^	0.172
Pulse pressure (mmHg)	0.42 (-1.33, 2.17) ^b^	0.635
Body Mass Index (kg/m2)	0.01 (-0.01,0.03) ^b^	0.441
Fasting glucose (mmol/L)	0.01 (-0.01, 0.02) ^b^	0.511
HOMA-B	0.01 (0.00, 0.03) ^b^	0.070

### Reverse associations of genetic liability to T2D and CHD with eBMD

Using genetic variants previously identified for T2D (94 SNPs) and CHD (52 SNPs) as instrumental variables, we found no convincing evidence of a causal relationship with eBMD, providing no support for reverse causality of T2D or CHD with eBMD ( Supplementary Table 11 and Supplementary Table 12).

## Discussion

The present study investigated a potential causal role of eBMD, assessed by quantitative ultrasound, in the development of T2D and CHD, using several complementary MR methods based on 235 eBMD-associated SNPs identified through GWAS in the UK Biobank. Conventional (IVW) MR analyses suggested causal effects of eBMD on risk of both T2D and CHD. These results were supported by several sensitivity analyses (MR-Egger, weighted median MR, weighted mode MR and MR-PRESSO) that make it unlikely that gross pleiotropic bias accounts for the associations we report. While the results of the observational and genetic estimates of eBMD with risk of T2D were consistent with each other, the genetic estimates with CHD differed from those in the observational analysis with CHD. The reasons for the discrepant results between the genetic and observational associations for CHD are unclear and warrant further investigation; scrutiny of the data suggests that there is an underlying causal relationship between eBMD and CHD, which is likely to be positive (i.e. higher eBMD causes higher risk of CHD). Taken together, the results of the present study suggest that estimated heel bone density has a modest causal association with risks of both T2D and CHD.

The findings of the present study are supported by biological and epidemiological studies that show associations of bone metabolism with insulin resistance, which may mediate risks of T2D and CHD ^17, 32– 35^ . In the past decade, bone tissue has emerged as an endocrine organ regulating a growing number of physiological processes including glucose homeostasis ^11, 34, 36– 38^, which is achieved through the secretion of osteocalcin, an osteoblast-derived hormone synthesized during bone formation ^18^. Observational studies have shown that lower circulating osteocalcin levels are associated with higher bone mineral density, impaired glucose tolerance and insulin resistance ^32, 39– 43^. Consistent with these results, we found that genetic elevation of eBMD was associated with insulin resistance phenotypes (e.g. HDL-cholesterol and HOMA-IR). Studies have found that genetic predisposition to insulin resistance confers higher risk of cardiovascular and metabolic disease, including T2D and CHD ^35^. Taken together, these studies and the present study suggest that the mechanism through which elevated eBMD is associated with higher risk of T2D and CHD may, at least in part, be mediated by increased insulin resistance.

The present study has several strengths. The discovery GWAS provided an abundant number of SNPs with which to generate a genetic instrument for eBMD, explaining a high (13.9%) proportion of variance of eBMD. The two-sample MR design allowed us then to apply these 235 SNPs to very large numbers of cases of CHD and T2D (from the largest GWAS studies conducted to date), maximising the statistical power and precision of estimates that we report. We tested the causal relationship between estimated heel bone mineral density with both T2D and CHD using recent state-of-the-art Mendelian randomization approaches yielding consistent effect estimates. Moreover, similar estimates were obtained using more stringent *p*-value thresholds to select SNPs entering our genetic instrument for eBMD and use of SNPs based solely on previously published studies. We found no evidence to support the presence of unbalanced horizontal pleiotropy, which can lead to violations of the instrumental variable assumptions ^6^. In addition, the SNP-exposure and SNP-outcome estimates were obtained from mostly European studies; therefore, population stratification bias is unlikely to affect the results of the present study. Furthermore, we used a bi-directional MR approach to investigate the causal directions eBMD and T2D and CHD, observing evidence for eBMD increasing the risk of T2D and CHD but not vice versa.

However, the present study also had several limitations. For observational analyses, we used cross-sectional data from the UKB, which is likely to be subject to various biases. In the cross-sectional study, individuals with CHD are likely to have lower physical activity resulting in lower eBMD ^44^, which would bias (through reverse causality) the association of eBMD with CHD and might explain the inverse association. However, the lack of a negative association of CHD SNPs with eBMD argues against the presence of CHD causally impacting on eBMD, and thus the discrepancy between the observational analysis and MR findings warrants further investigation. The causal estimates in this study could be susceptible to "winner's curse" as our SNP-eBMD associations were obtained from a discovery GWAS, with no replication cohort available. However, in the setting of two-sample MR where the SNP-exposure and SNP-outcome datasets do not overlap, the impact of "winner's curse" (leading to inflated estimates of SNP-eBMD and inclusion of potential false positive SNPs in the genetic instrument) would have a net effect to diminish the magnitude of MR estimate (i.e. any winner’s curse in our MR analysis would result in a more conservative causal estimate). More reliable estimates of SNP-eBMD associations derived from replication of our findings in other large-scale general population cohorts with measures of heel eBMD would enable us to rectify these issues. However, using different sets of SNPs based on various GWAS significance threshold (ranging from 5×10 ^-8^ to 5×10 ^-20^) showed consistent results arguing against potential winner’s curse leading to a major bias in the causal estimates ( Supplementary Table 9). With our genetic instrument explaining 13.9% of the variance of eBMD, weak instrument bias is very unlikely to affect our results; despite this, a major advantage of the two-sample MR design (where the SNP to exposure and SNP to outcome datasets are non-overlapping) is that any bias derived from potential weak instruments should lead to an attenuation of the effect estimate towards the null ^45^. Finally, as BMD is a quantification of multiple physiological pathways (an analogy might be made, for example, to height ^46^), it is unclear which one or more of these pathways is responsible for the causal associations with CHD and T2D that we report. Further studies could investigate individual traits (e.g. osteocalcin, glucagon-like peptides) that regulate BMD to gain a more comprehensive understanding of the underlying mechanisms underpinning these relationships.

It needs to be borne in mind in interpreting these results that the precise underlying physical determinants of eBMD are unclear. Previous studies have reported modest correlation coefficients between eBMD and BMD measured by DXA, ranging from 0.4–0.8 ^20^. As a positive control, we observed a strong casual association of eBMD with risk of fracture, which helps to validate eBMD as a reliable marker of bone health. The replication of SNPs reported by previous GWAS study of BMD in multiple skeletal sites measured by DXA suggests that the eBMD only partially reflects the same bone properties as BMD does. In addition to bone density, measures of eBMD may also reflect other properties of bone, such as the structural and elastic properties, which cannot be assessed by DXA ^20^. Recently, MR studies of the effect of calcium on coronary artery disease have reported positive associations of higher serum calcium and increased risk of CHD ^47, 48^, and a meta-analysis of randomized controlled trials suggest that increasing calcium intake results in modest increases in BMD ^49^. Taken together, this suggests that the causal relationship between higher serum calcium levels and increased risk of CHD may be mediated, at least in part, by elevated BMD. Alternatively, there may be causal pathways to CHD and T2D that result in higher eBMD, meaning that our findings are a marker of such a pathway (as opposed to being a causal mediator in the development of cardiometabolic disease). Dissecting which of the scenarios is present is challenging with existing methodologies. That said, the general consistency of our findings to: (i) various MR sensitivity analyses, (ii) using very strict GWAS *p*-value thresholds (up to *p*<5×10 ^-20^), (iii) using genetic instruments for alternative measures of BMD identified from prior studies, and (iv) the lack of reverse causality from bidirectional MR provides a framework in which, on balance, it is likely that a positive causal relationship exists between eBMD and cardiometabolic disease.

The findings of the present study suggest that higher bone density, measured by eBMD, may have an adverse effect on risk of cardiometabolic diseases, which may well have implications for patient care. Current drugs that are widely used to treat osteopaenia include bisphosphonates, which inhibit osteoclasts, reduce bone turnover and mildly increase BMD ^50^. A meta-analysis of 58 randomized trials of bisphosphates, reported that bisphosphates administered for 2–3 years had no effects on cardiovascular disease ^51^. While we recognise that our MR of the eBMD phenotype does not have direct relevance to any individual drug target (indeed, alternative frameworks are used for MRs of drug-targets) ^52^, the present study raises questions about the need for vigilance of the long-term cardiovascular consequences of drugs that alter bone density.

## Conclusions

In conclusion, Mendelian randomization provides evidence of a modest casual effect of elevated bone mineral density (assessed by quantitative ultrasound of heel) on risk of both T2D and CHD, which may be partially mediated by insulin resistance. The findings of this study add to the growing evidence-base suggesting a possible role of bone endocrine function in the pathogenesis of both type 2 diabetes and coronary heart disease.

## Data availability

The data referenced by this article are under copyright with the following copyright statement: Copyright: © 2017 Gan W et al.

The genetic and phenotypic UK Biobank data are available upon application to the UK Biobank ( https://www.ukbiobank.ac.uk/) to all *bona fide* researchers. The genome-wide association summary statistics for eBMD in 116,501 individuals from the UK Biobank study are available online ( http://mccarthy.well.ox.ac.uk/publications/2017/Gan_UKBB_INTERIM_eBMD_GWAS/) or via the UK Biobank’s Data Showcase ( http://biobank.ctsu.ox.ac.uk/crystal/), which can be accessed by researchers upon application.

## Supplementary material

Supplementary File 1: Association results for eBMD GWAS study, and results of sensitivity analyses using multiple Mendelian randomization approaches.
