# Supplementary material for: Bone mineral density and risk of type 2 diabetes and coronary heart disease: A Mendelian randomization study
Source: Wellcome Open Res. 2017 Aug 22;2:68. [Version 1] doi: 10.12688/wellcomeopenres.12288.1 (PMC5606062; doi:10.12688/wellcomeopenres.12288.1)
Supplement: Supplementary file 1 [file wellcomeopenres-2-13302-s0000.tgz › 7c8bc752-1c6b-492b-bed9-808ebf2c7edf.docx]

**Supplementary Table 1.** Significant loci for eBMD at *P* < 5×10^-8^ identified in GWAS study of 116,501 individuals from UK Biobank. The allelic effect size (β) on eBMD were standardized by rank-based inverse normal transformation. Signals were identified by GWAS analysis (Main signal) and conditional analysis (Secondary signal), reported in the “Analysis” column. Previous reported loci (in the “Known signal” column) were identified if the lead eBMD SNP was located within ±500kb of the reported lead variants and with LD r^2^>0.2.

| **SNP** | **Chr.** | **Position (bp)** | **Genes** | **Alleles** | | **EAF** | **Info Score** | **β** | **SE** | ***P* value** | **Analysis** | **Known signal** |
| --- | --- | --- | --- | --- | --- | --- | --- | --- | --- | --- | --- | --- |
|  |  |  | **Nearest** | **E** | **O** |  |  |  |  |  |  |  |
| rs139603701 | 1 | 2,904,634 | *ACTRT2* | A | G | 0.982 | 0.92 | 0.090 | 0.016 | 2.20E-09 | Main signal | No |
| rs2708632 | 1 | 8,464,509 | *RERE* | T | C | 0.343 | 1.00 | -0.032 | 0.004 | 2.40E-15 | Main signal | No |
| rs6429787 | 1 | 16,230,657 | *SPEN* | T | C | 0.892 | 1.00 | -0.043 | 0.006 | 1.50E-11 | Main signal | No |
| rs12751610 | 1 | 22,701,761 | *ZBTB40* | T | C | 0.825 | 1.00 | -0.068 | 0.005 | 8.30E-41 | Main signal | Yes |
| rs7519889 | 1 | 22,472,506 | *WNT4* | G | A | 0.796 | 0.99 | 0.044 | 0.005 | 1.10E-20 | Secondary signal | Yes |
| rs4654800 | 1 | 22,733,877 | *ZBTB40* | A | G | 0.510 | 1.00 | 0.048 | 0.004 | 9.50E-36 | Third signal | Yes |
| rs4589135 | 1 | 27,041,714 | *ARID1A* | T | C | 0.618 | 0.99 | 0.025 | 0.004 | 1.50E-09 | Main signal | No |
| rs10889644 | 1 | 67,145,322 | *SGIP1* | C | A | 0.437 | 0.99 | 0.025 | 0.004 | 3.80E-10 | Main signal | No |
| rs11209240 | 1 | 68,725,907 | *WLS* | A | C | 0.839 | 0.98 | -0.048 | 0.005 | 1.60E-20 | Main signal | Yes |
| rs4397637 | 1 | 68,603,131 | *LOC100289178* | G | A | 0.813 | 0.97 | -0.044 | 0.005 | 2.40E-16 | Secondary signal | No |
| rs10922492 | 1 | 89,304,059 | *PKN2* | T | A | 0.615 | 0.99 | -0.026 | 0.004 | 7.10E-11 | Main signal | No |
| rs3790608 | 1 | 113,055,023 | *WNT2B* | G | A | 0.849 | 0.99 | -0.046 | 0.006 | 1.10E-16 | Main signal | No |
| rs10923715 | 1 | 119,535,334 | *TBX15* | T | A | 0.551 | 0.99 | -0.027 | 0.004 | 3.60E-12 | Main signal | No |
| rs7556434 | 1 | 163,883,114 | *NUF2* | C | A | 0.532 | 0.99 | 0.025 | 0.004 | 5.30E-11 | Main signal | No |
| rs2421491 | 1 | 170,693,914 | *PRRX1* | T | C | 0.742 | 0.99 | -0.025 | 0.005 | 7.90E-09 | Main signal | No |
| rs484686 | 1 | 172,152,202 | *DNM3* | A | G | 0.497 | 0.99 | -0.030 | 0.004 | 2.20E-16 | Main signal | Yes |
| rs6684083 | 1 | 200,673,094 | *DDX59* | A | G | 0.389 | 1.00 | 0.027 | 0.004 | 3.90E-12 | Main signal | No |
| rs17514738 | 1 | 218,988,754 | *LOC643723* | T | C | 0.603 | 1.00 | 0.024 | 0.004 | 1.30E-08 | Main signal | No |
| rs6663745 | 1 | 220,097,906 | *SLC30A10* | T | G | 0.213 | 0.99 | 0.032 | 0.005 | 1.20E-11 | Main signal | No |
| rs4846574 | 1 | 219,846,938 | *RNU5F* | C | T | 0.419 | 0.99 | -0.025 | 0.004 | 4.10E-10 | Secondary signal | No |
| rs7527300 | 1 | 221,477,744 | *C1orf140* | C | T | 0.592 | 0.99 | 0.032 | 0.004 | 2.00E-15 | Main signal | No |
| rs1414660 | 1 | 240,586,695 | *FMN2* | C | T | 0.808 | 1.00 | -0.088 | 0.005 | 1.90E-70 | Main signal | Yes |
| rs10192375 | 2 | 28,892,116 | *PLB1* | A | G | 0.510 | 0.99 | -0.023 | 0.004 | 7.30E-09 | Main signal | No |
| rs10490046 | 2 | 40,630,678 | *SLC8A1* | A | C | 0.783 | 0.99 | 0.032 | 0.005 | 3.30E-12 | Main signal | No |
| rs7576782 | 2 | 42,218,378 | *LOC400950* | C | A | 0.813 | 0.99 | -0.056 | 0.005 | 1.00E-29 | Main signal | Yes |
| rs4305309 | 2 | 54,683,711 | *SPTBN1* | T | C | 0.358 | 0.99 | 0.079 | 0.004 | 1.10E-87 | Main signal | Yes |
| rs3106204 | 2 | 54,863,352 | *SPTBN1* | T | G | 0.296 | 1.00 | -0.044 | 0.004 | 2.00E-25 | Secondary signal | No |
| rs2302643 | 2 | 64,881,229 | *SERTAD2* | G | A | 0.554 | 0.98 | 0.023 | 0.004 | 4.90E-10 | Main signal | No |
| rs7578166 | 2 | 71,630,041 | *ZNF638* | A | C | 0.389 | 1.00 | -0.028 | 0.004 | 4.40E-11 | Main signal | No |
| rs115242848 | 2 | 119,507,607 | *EN1* | C | T | 0.992 | 0.92 | -0.411 | 0.023 | 1.30E-73 | Main signal | Yes |
| rs4073566 | 2 | 119,161,638 | *INSIG2* | C | A | 0.225 | 0.98 | 0.067 | 0.005 | 2.20E-45 | Secondary signal | Yes |
| rs62159864 | 2 | 119,590,951 | *EN1* | T | A | 0.728 | 0.99 | -0.054 | 0.004 | 1.50E-34 | Third signal | No |
| rs9973853 | 2 | 183,738,444 | *FRZB* | G | A | 0.745 | 0.99 | 0.027 | 0.005 | 5.30E-09 | Main signal | No |
| rs4675694 | 2 | 200,450,012 | *FLJ32063* | C | G | 0.173 | 0.97 | -0.037 | 0.005 | 2.90E-12 | Main signal | No |
| rs10931982 | 2 | 202,832,130 | *FZD7* | T | C | 0.226 | 1.00 | -0.057 | 0.005 | 1.00E-36 | Main signal | No |
| rs35593225 | 2 | 202,792,094 | *CDK15* | C | T | 0.882 | 1.00 | -0.043 | 0.006 | 9.10E-12 | Secondary signal | No |
| rs6741726 | 2 | 203,965,530 | *NBEAL1* | A | G | 0.032 | 1.00 | 0.076 | 0.011 | 4.80E-12 | Main signal | No |
| rs11681853 | 2 | 218,063,297 | *DIRC3* | C | G | 0.866 | 0.94 | -0.034 | 0.006 | 1.20E-08 | Main signal | No |
| rs2675952 | 2 | 233,790,522 | *NGEF* | T | A | 0.566 | 0.98 | -0.024 | 0.004 | 3.40E-10 | Main signal | No |
| rs2606737 | 3 | 11,398,654 | *ATG7* | A | G | 0.184 | 0.99 | 0.034 | 0.005 | 9.40E-11 | Main signal | No |
| rs1560633 | 3 | 25,548,555 | *RARB* | T | C | 0.362 | 0.97 | -0.024 | 0.004 | 3.80E-08 | Main signal | No |
| rs1599770 | 3 | 32,924,374 | *TRIM71* | A | C | 0.527 | 0.96 | 0.021 | 0.004 | 3.30E-08 | Main signal | No |
| rs370387 | 3 | 41,123,984 | *CTNNB1* | G | A | 0.442 | 1.00 | -0.047 | 0.004 | 3.50E-34 | Main signal | Yes |
| rs2526385 | 3 | 50,181,135 | *SEMA3F* | T | G | 0.195 | 1.00 | 0.030 | 0.005 | 7.20E-10 | Main signal | No |
| rs4974186 | 3 | 56,263,534 | *ERC2* | G | T | 0.447 | 0.99 | -0.027 | 0.004 | 6.80E-12 | Main signal | No |
| rs171080 | 3 | 156,555,500 | *LEKR1* | A | G | 0.879 | 1.00 | 0.049 | 0.006 | 4.20E-18 | Main signal | Yes |
| rs79664023 | 4 | 1,004,863 | *FGFRL1* | T | G | 0.856 | 0.98 | 0.076 | 0.006 | 7.70E-41 | Main signal | Yes |
| rs111632154 | 4 | 1,012,300 | *FGFRL1* | T | C | 0.951 | 0.98 | 0.082 | 0.009 | 4.00E-20 | Secondary signal | No |
| rs76865393 | 4 | 998,777 | *IDUA* | C | T | 0.937 | 0.99 | -0.084 | 0.008 | 2.30E-24 | Third signal | No |
| rs1386625 | 4 | 38,361,120 | *TBC1D1* | A | G | 0.098 | 0.97 | 0.044 | 0.007 | 3.90E-11 | Main signal | No |
| rs11729023 | 4 | 71,991,184 | *SLC4A4* | C | T | 0.878 | 0.98 | -0.036 | 0.006 | 9.70E-09 | Main signal | No |
| rs17010961 | 4 | 86,723,103 | *ARHGAP24* | T | A | 0.859 | 0.98 | -0.035 | 0.006 | 2.20E-09 | Main signal | No |
| rs1471251 | 4 | 87,976,359 | *AFF1* | A | T | 0.600 | 1.00 | -0.021 | 0.004 | 1.30E-08 | Main signal | No |
| rs11934731 | 4 | 88,831,249 | *HSP90AB3P* | G | A | 0.321 | 0.99 | 0.032 | 0.004 | 6.30E-15 | Main signal | Yes |
| rs13137552 | 4 | 88,715,324 | *IBSP* | T | C | 0.494 | 0.98 | 0.031 | 0.004 | 1.50E-14 | Secondary signal | No |
| rs2865339 | 4 | 95,099,605 | *SMARCAD1* | A | G | 0.531 | 0.97 | -0.023 | 0.004 | 1.70E-09 | Main signal | No |
| rs62315844 | 4 | 98,446,007 | *C4orf37* | T | C | 0.996 | 0.88 | -0.206 | 0.034 | 4.60E-10 | Main signal | No |
| rs6839437 | 4 | 146,174,631 | *OTUD4* | T | C | 0.164 | 1.00 | -0.039 | 0.005 | 4.40E-13 | Main signal | No |
| rs35428756 | 4 | 157,511,450 | *PDGFC* | GT | G | 0.608 | 0.99 | 0.023 | 0.004 | 1.50E-08 | Main signal | No |
| rs78014439 | 4 | 187,640,036 | *FAT1* | C | G | 0.930 | 0.99 | -0.043 | 0.008 | 2.20E-08 | Main signal | No |
| rs6870556 | 5 | 31,134,837 | *CDH6* | G | A | 0.376 | 0.99 | 0.024 | 0.004 | 2.00E-09 | Main signal | No |
| rs1428968 | 5 | 36,646,946 | *SLC1A3* | C | T | 0.821 | 0.99 | -0.036 | 0.005 | 2.00E-11 | Main signal | No |
| rs2542710 | 5 | 39,382,261 | *DAB2* | A | G | 0.513 | 0.99 | 0.028 | 0.004 | 1.10E-13 | Main signal | No |
| rs28744551 | 5 | 54,844,572 | *PPAP2A* | C | G | 0.907 | 0.99 | -0.039 | 0.007 | 1.80E-08 | Main signal | No |
| rs9986117 | 5 | 80,270,678 | *RASGRF2* | C | G | 0.686 | 0.99 | -0.025 | 0.004 | 3.50E-09 | Main signal | No |
| rs1010109 | 5 | 142,554,162 | *ARHGAP26* | A | G | 0.845 | 0.97 | 0.032 | 0.006 | 1.50E-08 | Main signal | No |
| rs368510 | 5 | 148,787,469 | *LOC728264* | G | A | 0.668 | 1.00 | -0.034 | 0.004 | 4.50E-16 | Main signal | No |
| rs393952 | 5 | 173,018,851 | *LOC285593* | G | A | 0.479 | 1.00 | 0.021 | 0.004 | 4.70E-08 | Main signal | No |
| rs4959677 | 6 | 2,500,820 | *C6orf195* | G | C | 0.510 | 0.99 | 0.026 | 0.004 | 3.10E-11 | Main signal | No |
| rs525678 | 6 | 7,058,857 | *RREB1* | A | G | 0.037 | 0.97 | 0.077 | 0.011 | 3.70E-14 | Main signal | No |
| rs9379084 | 6 | 7,231,843 | *RREB1* | G | A | 0.883 | 0.94 | 0.049 | 0.006 | 5.00E-14 | Secondary signal | No |
| rs74971894 | 6 | 21,888,517 | *FLJ22536* | A | G | 0.885 | 0.99 | 0.043 | 0.006 | 3.00E-12 | Main signal | No |
| rs9260620 | 6 | 29,923,091 | *HLA-A* | T | G | 0.668 | 0.99 | -0.025 | 0.004 | 1.90E-09 | Main signal | No |
| rs113166754 | 6 | 44,677,173 | *SUPT3H* | C | T | 0.938 | 0.99 | 0.102 | 0.008 | 1.50E-34 | Main signal | Yes |
| rs199815053 | 6 | 44,724,093 | *SUPT3H* | C | T | 0.465 | 0.99 | 0.040 | 0.004 | 7.60E-25 | Secondary signal | No |
| rs72868817 | 6 | 55,608,235 | *BMP5* | A | G | 0.932 | 0.99 | -0.059 | 0.008 | 4.80E-14 | Main signal | No |
| rs150445982 | 6 | 55,676,621 | *BMP5* | C | T | 0.975 | 0.84 | -0.099 | 0.014 | 3.50E-13 | Secondary signal | No |
| rs10943125 | 6 | 74,477,152 | *CD109* | C | T | 0.527 | 0.99 | 0.022 | 0.004 | 3.00E-09 | Main signal | No |
| rs4526167 | 6 | 83,739,225 | *UBE2CBP* | A | C | 0.301 | 0.99 | 0.029 | 0.004 | 2.10E-11 | Main signal | No |
| rs7763784 | 6 | 127,489,818 | *RSPO3* | C | T | 0.520 | 1.00 | -0.083 | 0.004 | 3.40E-100 | Main signal | Yes |
| rs7760760 | 6 | 130,379,852 | *L3MBTL3* | A | G | 0.318 | 0.98 | -0.026 | 0.004 | 6.30E-10 | Main signal | No |
| rs9483206 | 6 | 131,367,399 | *EPB41L2* | T | C | 0.623 | 1.00 | 0.031 | 0.004 | 6.30E-15 | Main signal | No |
| rs3777787 | 6 | 133,577,921 | *EYA4* | C | A | 0.459 | 0.99 | 0.059 | 0.004 | 2.20E-52 | Main signal | Yes |
| rs547545 | 6 | 133,812,022 | *EYA4* | T | A | 0.540 | 0.99 | -0.036 | 0.004 | 7.50E-19 | Secondary signal | No |
| rs3012465 | 6 | 133,350,936 | *EYA4* | A | G | 0.347 | 0.99 | 0.036 | 0.004 | 1.20E-19 | Third signal | No |
| rs12209685 | 6 | 136,228,675 | *PDE7B* | C | T | 0.620 | 0.98 | -0.027 | 0.004 | 1.90E-10 | Main signal | No |
| rs1891002 | 6 | 151,900,047 | *C6orf97* | T | A | 0.714 | 1.00 | 0.100 | 0.004 | 4.40E-119 | Main signal | Yes |
| rs2941740 | 6 | 152,009,638 | *ESR1* | A | G | 0.579 | 1.00 | -0.077 | 0.004 | 8.10E-87 | Secondary signal | Yes |
| rs1890010 | 6 | 152,085,275 | *ESR1* | C | T | 0.290 | 0.99 | -0.051 | 0.004 | 1.50E-32 | Third signal | No |
| rs6903009 | 6 | 155,352,813 | *TIAM2* | A | G | 0.646 | 0.98 | -0.022 | 0.004 | 1.40E-08 | Main signal | No |
| rs73029263 | 6 | 164,113,762 | *QKI* | A | G | 0.869 | 0.99 | 0.030 | 0.006 | 2.80E-08 | Main signal | No |
| rs2189446 | 7 | 15,632,606 | *MEOX2* | T | C | 0.315 | 0.99 | -0.033 | 0.004 | 4.70E-14 | Main signal | No |
| rs3095208 | 7 | 20,331,713 | *ITGB8* | T | C | 0.738 | 0.99 | -0.026 | 0.005 | 6.20E-10 | Main signal | No |
| rs85 | 7 | 25,702,026 | *MIR148A* | T | C | 0.198 | 1.00 | -0.043 | 0.005 | 9.30E-18 | Main signal | No |
| rs62454420 | 7 | 27,191,804 | *LOC100133311* | A | G | 0.930 | 0.98 | -0.049 | 0.008 | 2.00E-09 | Main signal | No |
| rs17501090 | 7 | 27,221,454 | *HOXA11* | C | A | 0.977 | 0.95 | 0.082 | 0.013 | 6.70E-09 | Secondary signal | No |
| rs10244184 | 7 | 27,932,076 | *JAZF1* | T | C | 0.744 | 0.99 | 0.042 | 0.005 | 9.00E-19 | Main signal | No |
| rs757980 | 7 | 28,725,536 | *CREB5* | G | A | 0.251 | 0.96 | 0.041 | 0.005 | 6.60E-19 | Main signal | No |
| rs10236571 | 7 | 30,956,223 | *AQP1* | A | G | 0.764 | 0.99 | 0.052 | 0.005 | 2.20E-29 | Main signal | No |
| rs1717747 | 7 | 38,097,903 | *EPDR1* | A | G | 0.816 | 1.00 | -0.076 | 0.005 | 5.50E-52 | Main signal | Yes |
| rs1052974 | 7 | 37,946,540 | *SFRP4* | C | A | 0.814 | 0.99 | -0.054 | 0.005 | 3.80E-27 | Secondary signal | Yes |
| rs1721382 | 7 | 38,104,983 | *STARD3NL* | C | A | 0.348 | 1.00 | -0.055 | 0.004 | 3.30E-41 | Third signal | Yes |
| rs643044 | 7 | 83,302,264 | *SEMA3E* | G | A | 0.685 | 0.99 | 0.024 | 0.004 | 1.70E-08 | Main signal | No |
| rs42038 | 7 | 92,243,719 | *CDK6* | C | T | 0.702 | 0.99 | 0.024 | 0.004 | 4.80E-08 | Main signal | No |
| rs7781370 | 7 | 96,133,531 | *FLJ42280* | T | C | 0.336 | 1.00 | -0.047 | 0.004 | 1.60E-30 | Main signal | Yes |
| rs212417 | 7 | 105,494,963 | *ATXN7L1* | G | A | 0.328 | 0.99 | 0.032 | 0.004 | 9.10E-14 | Main signal | No |
| rs2707518 | 7 | 120,954,908 | *WNT16* | G | T | 0.607 | 0.98 | -0.170 | 0.004 | 0.00E+00 | Main signal | Yes |
| rs3779381 | 7 | 120,966,790 | *WNT16* | A | G | 0.741 | 1.00 | -0.178 | 0.005 | 0.00E+00 | Secondary signal | No |
| rs62621812 | 7 | 127,015,083 | *ZNF800* | G | A | 0.978 | 1.00 | 0.092 | 0.014 | 2.80E-13 | Main signal | No |
| rs2929308 | 8 | 9,084,121 | *PPP1R3B* | T | A | 0.493 | 1.00 | 0.041 | 0.004 | 1.40E-26 | Main signal | No |
| rs4240624 | 8 | 9,184,231 | *LOC157273* | G | A | 0.093 | 1.00 | 0.058 | 0.007 | 1.10E-16 | Secondary signal | No |
| rs6471752 | 8 | 49,309,167 | *EFCAB1* | C | T | 0.848 | 0.99 | 0.033 | 0.006 | 1.20E-09 | Main signal | No |
| rs7003794 | 8 | 71,789,146 | *XKR9* | C | A | 0.394 | 0.99 | 0.030 | 0.004 | 6.20E-14 | Main signal | Yes |
| rs114847962 | 8 | 72,217,903 | *EYA1* | A | T | 0.739 | 0.99 | 0.031 | 0.005 | 1.30E-12 | Secondary signal | No |
| rs6999925 | 8 | 80,786,648 | *MRPS28* | A | G | 0.376 | 0.99 | 0.024 | 0.004 | 3.20E-09 | Main signal | No |
| rs446027 | 8 | 81,452,333 | *ZBTB10* | A | G | 0.686 | 1.00 | 0.023 | 0.004 | 5.00E-08 | Main signal | No |
| rs2737252 | 8 | 116,663,898 | *TRPS1* | G | A | 0.715 | 0.99 | -0.042 | 0.004 | 1.80E-23 | Main signal | No |
| rs2205270 | 8 | 117,158,377 | *TRPS1* | C | T | 0.135 | 0.96 | 0.039 | 0.006 | 4.00E-10 | Secondary signal | No |
| rs117108011 | 8 | 119,901,442 | *TNFRSF11B* | A | G | 0.984 | 0.91 | -0.109 | 0.016 | 1.50E-11 | Main signal | Yes |
| rs1487241 | 8 | 128,021,488 | *POU5F1B* | A | T | 0.321 | 0.99 | 0.025 | 0.004 | 9.80E-09 | Main signal | No |
| rs6475068 | 9 | 16,693,216 | *BNC2* | G | C | 0.097 | 0.99 | -0.043 | 0.007 | 6.70E-11 | Main signal | No |
| rs138945742 | 9 | 18,645,278 | *ADAMTSL1* | G | T | 0.963 | 0.98 | 0.057 | 0.011 | 2.70E-08 | Main signal | No |
| rs10992867 | 9 | 96,461,013 | *PHF2* | G | A | 0.747 | 0.99 | -0.030 | 0.005 | 1.80E-11 | Main signal | No |
| rs1057713 | 9 | 96,714,161 | *BARX1* | A | G | 0.604 | 0.99 | -0.020 | 0.004 | 2.70E-08 | Secondary signal | No |
| rs1877456 | 9 | 98,275,097 | *PTCH1* | C | G | 0.777 | 0.98 | 0.032 | 0.005 | 2.10E-12 | Main signal | No |
| rs10979249 | 9 | 110,907,520 | *KLF4* | G | T | 0.773 | 0.98 | 0.033 | 0.005 | 2.80E-13 | Main signal | No |
| rs3802342 | 9 | 133,473,303 | *FUBP3* | C | G | 0.648 | 0.99 | 0.030 | 0.004 | 5.30E-13 | Main signal | Yes |
| rs2519093 | 9 | 136,141,870 | *ABO* | C | T | 0.815 | 0.99 | 0.029 | 0.005 | 5.50E-09 | Main signal | No |
| rs74119759 | 10 | 20,143,109 | *PLXDC2* | C | T | 0.841 | 0.96 | 0.034 | 0.006 | 6.10E-09 | Main signal | No |
| rs6482632 | 10 | 27,890,672 | *RAB18* | T | C | 0.493 | 1.00 | 0.024 | 0.004 | 2.80E-09 | Main signal | No |
| rs12354769 | 10 | 28,531,051 | *MPP7* | T | C | 0.860 | 0.99 | -0.041 | 0.006 | 1.10E-13 | Main signal | Yes |
| rs7919498 | 10 | 31,047,997 | *ZNF438* | C | T | 0.484 | 1.00 | -0.035 | 0.004 | 3.40E-20 | Main signal | No |
| rs10824760 | 10 | 54,425,325 | *MBL2* | C | T | 0.108 | 0.98 | -0.138 | 0.006 | 2.50E-109 | Main signal | Yes |
| rs17662822 | 10 | 54,412,481 | *MBL2* | C | A | 0.674 | 0.99 | 0.041 | 0.004 | 5.90E-22 | Secondary signal | No |
| rs1159798 | 10 | 54,412,493 | *MBL2* | A | C | 0.222 | 1.00 | 0.072 | 0.005 | 8.10E-52 | Third signal | No |
| rs1877998 | 10 | 79,411,740 | *KCNMA1* | G | A | 0.818 | 1.00 | 0.034 | 0.005 | 2.20E-11 | Main signal | Yes |
| rs17173698 | 10 | 89,468,953 | *PAPSS2* | G | A | 0.974 | 1.00 | -0.074 | 0.013 | 9.00E-10 | Main signal | No |
| rs603424 | 10 | 102,075,479 | *PKD2L1* | G | A | 0.830 | 1.00 | 0.032 | 0.005 | 2.80E-09 | Main signal | Yes |
| rs10885447 | 10 | 115,166,770 | *HABP2* | G | A | 0.789 | 0.98 | -0.030 | 0.005 | 6.00E-10 | Main signal | No |
| rs11196170 | 10 | 114,722,621 | *TCF7L2* | G | A | 0.790 | 0.99 | 0.026 | 0.005 | 3.90E-08 | Secondary signal | No |
| rs80226362 | 10 | 134,413,500 | *INPP5A* | G | T | 0.816 | 1.00 | -0.037 | 0.005 | 1.50E-13 | Main signal | No |
| rs2653559 | 11 | 8,900,394 | *ST5* | C | T | 0.836 | 0.99 | 0.031 | 0.005 | 1.20E-09 | Main signal | No |
| rs61880664 | 11 | 15,343,532 | *INSC* | A | G | 0.898 | 0.98 | -0.044 | 0.007 | 5.10E-12 | Main signal | No |
| rs78152188 | 11 | 16,635,424 | *C11orf58* | C | G | 0.712 | 0.99 | -0.032 | 0.004 | 6.80E-13 | Main signal | No |
| rs11029901 | 11 | 27,287,108 | *CCDC34* | A | G | 0.359 | 0.99 | 0.044 | 0.004 | 2.10E-27 | Main signal | No |
| rs117941208 | 11 | 27,346,727 | *CCDC34* | C | T | 0.889 | 0.97 | 0.043 | 0.006 | 7.70E-12 | Secondary signal | No |
| rs2098878 | 11 | 35,096,310 | *CD44* | A | G | 0.461 | 1.00 | -0.031 | 0.004 | 2.10E-15 | Main signal | No |
| rs4579897 | 11 | 47,097,364 | *C11orf49* | G | T | 0.325 | 0.99 | 0.046 | 0.004 | 2.00E-27 | Main signal | Yes |
| rs1530914 | 11 | 60,028,940 | *MS4A4A* | C | T | 0.401 | 1.00 | -0.032 | 0.004 | 1.90E-15 | Main signal | No |
| rs174574 | 11 | 61,600,342 | *FADS2* | A | C | 0.354 | 1.00 | 0.024 | 0.004 | 1.10E-08 | Main signal | No |
| rs117111740 | 11 | 62,201,239 | *AHNAK* | T | C | 0.973 | 0.96 | 0.133 | 0.012 | 5.40E-25 | Main signal | No |
| rs4930295 | 11 | 65,390,554 | *PCNXL3* | C | G | 0.777 | 1.00 | 0.030 | 0.005 | 1.40E-10 | Main signal | No |
| rs4988321 | 11 | 68,174,189 | *LRP5* | G | A | 0.949 | 1.00 | 0.080 | 0.009 | 1.20E-20 | Main signal | Yes |
| rs61887821 | 11 | 68,099,622 | *LRP5* | G | A | 0.994 | 0.95 | 0.189 | 0.027 | 1.90E-13 | Secondary signal | No |
| rs1060435 | 11 | 68,855,595 | *TPCN2* | A | G | 0.593 | 1.00 | -0.030 | 0.004 | 3.40E-16 | Main signal | No |
| rs649693 | 11 | 86,869,577 | *TMEM135* | T | C | 0.692 | 0.99 | 0.070 | 0.004 | 4.20E-61 | Main signal | Yes |
| rs149504726 | 11 | 86,653,988 | *FZD4* | G | A | 0.994 | 0.85 | -0.190 | 0.027 | 7.10E-13 | Secondary signal | No |
| rs7124639 | 11 | 112,450,073 | *C11orf34* | A | G | 0.401 | 1.00 | 0.054 | 0.004 | 6.50E-44 | Main signal | No |
| rs10891479 | 11 | 112,826,303 | *NCAM1* | G | A | 0.818 | 0.93 | 0.034 | 0.005 | 8.50E-11 | Secondary signal | No |
| rs7125361 | 11 | 115,080,042 | *CADM1* | C | G | 0.579 | 0.99 | -0.042 | 0.004 | 1.70E-26 | Main signal | No |
| rs2846901 | 11 | 115,483,879 | *CADM1* | A | G | 0.147 | 0.99 | 0.033 | 0.006 | 2.60E-09 | Secondary signal | No |
| rs10790255 | 11 | 118,515,579 | *PHLDB1* | G | T | 0.247 | 0.99 | -0.031 | 0.005 | 2.80E-11 | Main signal | No |
| rs1622638 | 11 | 121,800,971 | *MIR100HG* | G | A | 0.607 | 1.00 | -0.022 | 0.004 | 6.10E-09 | Main signal | No |
| rs4980826 | 12 | 578,349 | *B4GALNT3* | C | A | 0.605 | 1.00 | -0.025 | 0.004 | 2.80E-10 | Main signal | No |
| rs9668575 | 12 | 1,636,381 | *LOC100292680* | A | G | 0.745 | 0.95 | -0.037 | 0.005 | 2.20E-16 | Main signal | Yes |
| rs7959604 | 12 | 1,637,129 | *LOC100292680* | C | G | 0.925 | 0.99 | 0.058 | 0.008 | 3.70E-15 | Secondary signal | No |
| rs56151937 | 12 | 2,502,996 | *CACNA1C* | T | G | 0.847 | 0.99 | -0.036 | 0.006 | 1.10E-10 | Main signal | No |
| rs117481343 | 12 | 13,328,208 | *EMP1* | C | T | 0.970 | 0.93 | -0.135 | 0.012 | 8.00E-31 | Main signal | No |
| rs118115924 | 12 | 49,379,537 | *WNT1* | G | T | 0.988 | 1.00 | 0.176 | 0.018 | 2.80E-23 | Main signal | No |
| rs10875906 | 12 | 49,385,679 | *DDN* | C | T | 0.721 | 0.97 | -0.030 | 0.005 | 9.50E-11 | Secondary signal | No |
| rs11832031 | 12 | 65,952,149 | *MSRB3* | T | A | 0.232 | 0.98 | 0.027 | 0.005 | 8.20E-10 | Main signal | No |
| rs10858944 | 12 | 90,419,192 | *LOC338758* | G | A | 0.604 | 1.00 | -0.060 | 0.004 | 4.30E-50 | Main signal | No |
| rs10859561 | 12 | 94,107,712 | *CRADD* | C | T | 0.487 | 0.99 | -0.023 | 0.004 | 4.20E-08 | Main signal | No |
| rs112073168 | 12 | 108,985,976 | *TMEM119* | G | A | 0.972 | 0.96 | 0.065 | 0.012 | 1.40E-08 | Main signal | No |
| rs8002850 | 13 | 22,811,940 | *FGF9* | G | A | 0.664 | 1.00 | 0.027 | 0.004 | 2.30E-12 | Main signal | No |
| rs1328042 | 13 | 37,466,947 | *SMAD9* | G | A | 0.760 | 1.00 | -0.026 | 0.005 | 6.80E-09 | Main signal | No |
| rs8001611 | 13 | 42,965,694 | *AKAP11* | C | T | 0.488 | 1.00 | -0.047 | 0.004 | 6.80E-35 | Main signal | Yes |
| rs770379 | 13 | 51,139,771 | *DLEU7* | A | G | 0.457 | 1.00 | 0.027 | 0.004 | 6.80E-11 | Main signal | No |
| rs3812849 | 13 | 74,701,736 | *KLF12* | A | C | 0.739 | 0.99 | 0.027 | 0.005 | 3.80E-09 | Main signal | No |
| rs7998877 | 13 | 94,428,340 | *GPC6* | G | T | 0.040 | 0.98 | -0.053 | 0.010 | 1.40E-08 | Main signal | No |
| rs2008411 | 13 | 99,587,929 | *DOCK9* | C | T | 0.302 | 1.00 | 0.039 | 0.004 | 1.30E-19 | Main signal | No |
| rs1042704 | 14 | 23,312,594 | *MMP14* | G | A | 0.784 | 1.00 | 0.031 | 0.005 | 3.10E-10 | Main signal | No |
| rs10145299 | 14 | 35,215,602 | *BAZ1A* | T | C | 0.508 | 0.97 | -0.025 | 0.004 | 1.40E-10 | Main signal | No |
| rs4444235 | 14 | 54,410,919 | *BMP4* | T | C | 0.538 | 1.00 | -0.054 | 0.004 | 1.80E-43 | Main signal | No |
| rs2761887 | 14 | 54,425,052 | *BMP4* | C | A | 0.452 | 1.00 | -0.053 | 0.004 | 3.60E-40 | Secondary signal | No |
| rs2588829 | 14 | 68,612,920 | *RAD51L1* | G | A | 0.165 | 1.00 | -0.028 | 0.005 | 1.10E-08 | Main signal | No |
| rs56375669 | 14 | 95,629,615 | *FLJ45244* | T | C | 0.913 | 0.99 | 0.043 | 0.007 | 6.90E-10 | Main signal | No |
| rs10139279 | 14 | 103,926,454 | *MARK3* | G | A | 0.662 | 0.99 | 0.041 | 0.004 | 4.90E-24 | Main signal | Yes |
| rs11636403 | 15 | 51,548,744 | *CYP19A1* | C | T | 0.542 | 0.97 | -0.035 | 0.004 | 8.40E-17 | Main signal | No |
| rs28587205 | 15 | 67,427,897 | *SMAD3* | A | T | 0.523 | 0.99 | 0.025 | 0.004 | 2.70E-10 | Main signal | No |
| rs2002122 | 15 | 70,594,071 | *TLE3* | T | G | 0.443 | 0.99 | -0.027 | 0.004 | 8.50E-12 | Main signal | No |
| rs11637971 | 15 | 91,079,863 | *CRTC3* | A | C | 0.303 | 1.00 | -0.026 | 0.004 | 5.90E-10 | Main signal | No |
| rs34676698 | 16 | 407,549 | *AXIN1* | G | A | 0.966 | 0.67 | 0.108 | 0.013 | 5.80E-16 | Main signal | Yes |
| rs2301522 | 16 | 359,953 | *AXIN1* | A | G | 0.335 | 0.99 | 0.032 | 0.004 | 2.00E-14 | Secondary signal | No |
| rs6497636 | 16 | 23,053,035 | *USP31* | G | A | 0.322 | 0.96 | -0.025 | 0.004 | 2.40E-08 | Main signal | No |
| rs72805220 | 16 | 51,903,981 | *LOC388276* | C | A | 0.934 | 0.95 | 0.065 | 0.008 | 4.80E-15 | Main signal | No |
| rs1381448 | 16 | 51,723,131 | *LOC388276* | G | A | 0.334 | 1.00 | 0.029 | 0.004 | 4.70E-13 | Secondary signal | No |
| rs17680862 | 16 | 67,322,118 | *PLEKHG4* | G | C | 0.974 | 1.00 | 0.085 | 0.012 | 6.40E-14 | Main signal | No |
| rs8064086 | 16 | 67,013,043 | *CES3* | G | C | 0.865 | 0.99 | -0.035 | 0.006 | 7.20E-10 | Secondary signal | No |
| rs4888151 | 16 | 81,559,009 | *CMIP* | A | C | 0.257 | 0.97 | 0.027 | 0.005 | 5.50E-09 | Main signal | No |
| rs71390846 | 16 | 86,714,715 | *FOXL1* | G | C | 0.810 | 0.98 | 0.041 | 0.005 | 5.60E-16 | Main signal | Yes |
| rs113478686 | 16 | 88,850,897 | *FAM38A* | C | CT | 0.773 | 0.99 | 0.025 | 0.005 | 4.80E-08 | Main signal | No |
| rs2663339 | 17 | 927,516 | *ABR* | A | C | 0.692 | 0.99 | 0.027 | 0.004 | 6.10E-11 | Main signal | No |
| rs8072532 | 17 | 2,045,273 | *SMG6* | G | A | 0.302 | 1.00 | -0.055 | 0.004 | 8.30E-39 | Main signal | Yes |
| rs11078596 | 17 | 1,618,262 | *C17orf91* | C | T | 0.818 | 1.00 | 0.029 | 0.005 | 1.00E-08 | Secondary signal | No |
| rs74439044 | 17 | 7,781,019 | *CHD3* | T | C | 0.904 | 0.99 | 0.044 | 0.007 | 4.70E-11 | Main signal | No |
| rs117573122 | 17 | 7,388,716 | *POLR2A* | G | C | 0.993 | 0.86 | -0.140 | 0.026 | 2.40E-09 | Secondary signal | No |
| rs77152265 | 17 | 9,138,318 | *NTN1* | T | C | 0.841 | 0.96 | 0.035 | 0.006 | 5.10E-10 | Main signal | No |
| rs3760456 | 17 | 27,948,844 | *CORO6* | C | T | 0.559 | 1.00 | 0.034 | 0.004 | 1.60E-17 | Main signal | No |
| rs7215205 | 17 | 29,818,258 | *RAB11FIP4* | T | C | 0.367 | 0.98 | 0.024 | 0.004 | 4.40E-09 | Main signal | No |
| rs143043662 | 17 | 39,913,771 | *JUP* | C | T | 0.986 | 1.00 | -0.092 | 0.017 | 9.30E-09 | Main signal | No |
| rs7209826 | 17 | 41,796,406 | *SOST* | A | G | 0.619 | 0.97 | -0.044 | 0.004 | 2.10E-28 | Main signal | Yes |
| rs76410205 | 17 | 41,807,508 | *SOST* | C | T | 0.916 | 0.99 | -0.066 | 0.007 | 2.30E-21 | Secondary signal | No |
| rs2732581 | 17 | 44,326,619 | *KIAA1267* | A | G | 0.762 | 0.97 | 0.023 | 0.005 | 3.10E-08 | Main signal | Yes |
| rs2696264 | 17 | 48,333,349 | *TMEM92* | G | A | 0.771 | 1.00 | -0.031 | 0.005 | 7.00E-12 | Main signal | No |
| rs72829754 | 17 | 54,233,702 | *ANKFN1* | G | A | 0.602 | 0.99 | -0.036 | 0.004 | 2.40E-19 | Main signal | No |
| rs1036902 | 17 | 58,950,791 | *BCAS3* | T | C | 0.853 | 1.00 | -0.051 | 0.006 | 5.10E-21 | Main signal | No |
| rs11869530 | 17 | 63,549,979 | *AXIN2* | G | A | 0.363 | 1.00 | -0.028 | 0.004 | 5.60E-12 | Main signal | No |
| rs73997493 | 17 | 75,319,800 | *SEPT9* | C | T | 0.913 | 0.99 | 0.047 | 0.007 | 2.20E-10 | Main signal | No |
| rs12150031 | 17 | 79,420,224 | *BAHCC1* | C | G | 0.610 | 1.00 | 0.024 | 0.004 | 2.20E-08 | Main signal | No |
| rs11875132 | 18 | 10,348,334 | *APCDD1* | T | C | 0.565 | 0.98 | 0.025 | 0.004 | 3.80E-10 | Main signal | No |
| rs4430817 | 18 | 13,682,666 | *C18orf19* | G | C | 0.643 | 1.00 | 0.049 | 0.004 | 5.40E-32 | Main signal | Yes |
| rs12967019 | 18 | 46,474,178 | *SMAD7* | T | C | 0.509 | 0.95 | -0.023 | 0.004 | 3.20E-09 | Main signal | No |
| rs2957137 | 18 | 60,066,888 | *TNFRSF11A* | A | T | 0.360 | 0.98 | -0.023 | 0.004 | 4.10E-09 | Main signal | Yes |
| rs657693 | 18 | 77,162,462 | *NFATC1* | A | G | 0.637 | 1.00 | -0.022 | 0.004 | 4.10E-08 | Main signal | No |
| rs11084888 | 19 | 1,162,376 | *SBNO2* | G | A | 0.689 | 0.99 | 0.037 | 0.004 | 1.10E-17 | Main signal | No |
| rs8108787 | 19 | 817,980 | *LPPR3* | T | C | 0.738 | 0.97 | 0.033 | 0.005 | 9.30E-12 | Secondary signal | No |
| rs12609327 | 19 | 2,183,000 | *DOT1L* | A | C | 0.500 | 0.99 | 0.024 | 0.004 | 1.30E-09 | Main signal | No |
| rs60507951 | 19 | 33,544,187 | *RHPN2* | G | A | 0.908 | 0.99 | -0.111 | 0.007 | 2.10E-59 | Main signal | Yes |
| rs28364580 | 19 | 41,724,885 | *AXL* | G | A | 0.752 | 0.96 | 0.026 | 0.005 | 1.80E-09 | Main signal | No |
| rs3170167 | 19 | 45,976,718 | *FOSB* | T | C | 0.850 | 0.99 | 0.036 | 0.006 | 2.10E-10 | Main signal | No |
| rs71338564 | 20 | 6,405,944 | *FERMT1* | G | A | 0.647 | 0.98 | -0.035 | 0.004 | 3.60E-16 | Main signal | No |
| rs6117854 | 20 | 7,551,554 | *HAO1* | G | A | 0.671 | 0.98 | 0.038 | 0.004 | 2.50E-19 | Main signal | Yes |
| rs6040286 | 20 | 10,985,143 | *LOC339593* | T | C | 0.438 | 1.00 | -0.041 | 0.004 | 4.40E-24 | Main signal | Yes |
| rs35308216 | 20 | 10,629,525 | *JAG1* | T | C | 0.920 | 1.00 | 0.065 | 0.007 | 9.90E-20 | Secondary signal | Yes |
| rs17457340 | 20 | 10,640,042 | *JAG1* | T | C | 0.922 | 0.99 | 0.068 | 0.007 | 3.60E-21 | Third signal | No |
| rs34778574 | 20 | 11,150,594 | *LOC339593* | T | C | 0.962 | 1.00 | 0.073 | 0.011 | 3.70E-12 | Fourth signal | No |
| rs13044413 | 20 | 33,020,957 | *ITCH* | A | G | 0.560 | 1.00 | -0.023 | 0.004 | 3.70E-08 | Main signal | No |
| rs6016547 | 20 | 39,962,991 | *LPIN3* | A | C | 0.836 | 0.99 | 0.029 | 0.005 | 2.70E-09 | Main signal | No |
| rs1150442 | 20 | 45,603,836 | *EYA2* | T | C | 0.256 | 0.98 | -0.032 | 0.005 | 3.10E-12 | Main signal | No |
| rs2830907 | 21 | 28,770,608 | *NCRNA00113* | G | A | 0.537 | 1.00 | -0.031 | 0.004 | 3.90E-15 | Main signal | No |
| rs9982895 | 21 | 40,343,087 | *ETS2* | T | C | 0.273 | 0.99 | 0.045 | 0.004 | 1.80E-23 | Main signal | No |
| rs465004 | 21 | 40,021,851 | *ERG* | T | C | 0.237 | 0.98 | -0.035 | 0.005 | 1.60E-13 | Secondary signal | No |
| rs9606139 | 22 | 19,679,303 | *SEPT5* | G | A | 0.890 | 0.95 | 0.117 | 0.007 | 3.40E-77 | Main signal | No |
| rs2049939 | 22 | 29,483,920 | *KREMEN1* | G | C | 0.350 | 1.00 | 0.042 | 0.004 | 5.90E-25 | Main signal | No |

Notes: E, Effect allele; O, other allele; EAF, effect allele frequency

**Supplementary Table 2:** Secondary signals at *p* < 5×10^-8^ for eBMD identified by conditional analysis. SNPs in LD r^2^>0.05 with lead SNPs are highlighted in red. LD r^2^ was calculated based on EUR in 1000 Genomes Project. β is the effect size on eBMD, which is standardized by rank-based inverse normal transformation, per effect allele based on the additive genetic model.

| **SNP** | **Chr.** | **Position (bp)** | **Genes** | **Alleles** | | **Effect allele frequency** | **β** | **SE** | ***P* value** | **r^2^ with lead SNP in main signal** | **r^2^ with lead SNP in secondary signal** |
| --- | --- | --- | --- | --- | --- | --- | --- | --- | --- | --- | --- |
|  |  |  | **Nearest** | **Effect** | **Other** |  |  |  |  |  |  |
| **Secondary signals reaching GWS by conditional analysis** | | | | |  |  |  |  |  |  |  |
| rs7519889 | 1 | 22,472,506 | *WNT4* | G | A | 0.796 | 0.044538 | 0.00493503 | 2.9E-21 | 0.0067 | - |
| rs4397637 | 1 | 68,603,131 | *LOC100289178* | G | A | 0.813 | -0.04646 | 0.00517295 | 1.1E-18 | 0.0062 | - |
| rs4846574 | 1 | 219,846,938 | *RNU5F* | C | T | 0.419 | -0.02655 | 0.00403566 | 1.3E-11 | 0.0114 | - |
| rs3106204 | 2 | 54,863,352 | *SPTBN1* | T | G | 0.296 | -0.04896 | 0.00435788 | 4.7E-31 | 0.0004 | - |
| rs4073566 | 2 | 119,161,638 | *INSIG2* | C | A | 0.225 | 0.061537 | 0.00479999 | 6.2E-39 | 0.0048 | - |
| rs35593225 | 2 | 202,792,094 | *CDK15* | C | T | 0.882 | -0.04231 | 0.00615599 | 1.1E-11 | 0.0015 | - |
| rs111632154 | 4 | 1,012,300 | *FGFRL1* | T | C | 0.951 | 0.095048 | 0.00930319 | 4.8E-26 | 0.0093 | - |
| rs13137552 | 4 | 88,715,324 | *IBSP* | T | C | 0.494 | 0.031369 | 0.00402824 | 1.2E-14 | 0.0004 | - |
| rs9379084 | 6 | 7,231,843 | *RREB1* | G | A | 0.883 | 0.046889 | 0.00638596 | 7.8E-13 | 0.0063 | - |
| rs199815053 | 6 | 44,724,093 | *SUPT3H* | C | T | 0.465 | 0.02987 | 0.0040963 | 8.8E-14 | 0.0586 | - |
| rs150445982 | 6 | 55,676,621 | *BMP5* | C | T | 0.975 | -0.10501 | 0.0138493 | 9.1E-15 | 0.0009 | - |
| rs547545 | 6 | 133,812,022 | *EYA4* | T | A | 0.540 | -0.02852 | 0.00402471 | 4.3E-12 | 0.0122 | - |
| rs2941740 | 6 | 152,009,638 | *ESR1* | A | G | 0.579 | -0.06941 | 0.00403647 | 4.8E-70 | 0.0041 | - |
| rs17501090 | 7 | 27,221,454 | *HOXA11* | C | A | 0.977 | 0.080129 | 0.0134803 | 1.8E-08 | 0 | - |
| rs1052974 | 7 | 37,946,540 | *SFRP4* | C | A | 0.814 | -0.05196 | 0.00511364 | 1.7E-25 | 0 | - |
| rs3779381 | 7 | 120,966,790 | *WNT16* | A | G | 0.741 | -0.08959 | 0.00647242 | 3.2E-45 | 0.5224 | - |
| rs4240624 | 8 | 9,184,231 | *LOC157273* | G | A | 0.093 | 0.040716 | 0.00719009 | 4.7E-08 | 0.0656 | - |
| rs114847962 | 8 | 72,217,903 | *EYA1* | A | T | 0.739 | 0.030642 | 0.00456537 | 1E-12 | 0.0001 | - |
| rs2205270 | 8 | 117,158,377 | *TRPS1* | C | T | 0.135 | 0.043068 | 0.00597462 | 2E-12 | 0.0026 | - |
| rs1057713 | 9 | 96,714,161 | *BARX1* | A | G | 0.604 | -0.02088 | 0.00408361 | 1.2E-08 | 0 | - |
| rs17662822 | 10 | 54,412,481 | *MBL2* | C | A | 0.674 | 0.063733 | 0.00437969 | 5.4E-50 | 0.0444 | - |
| rs11196170 | 10 | 114,722,621 | *TCF7L2* | G | A | 0.790 | 0.027014 | 0.00489905 | 8.4E-09 | 0.0001 | - |
| rs117941208 | 11 | 27,346,727 | *CCDC34* | C | T | 0.889 | 0.036324 | 0.00644691 | 9.2E-09 | 0.0144 | - |
| rs61887821 | 11 | 68,099,622 | *LRP5* | G | A | 0.994 | 0.197984 | 0.026721 | 9.9E-15 | 0.0001 | - |
| rs149504726 | 11 | 86,653,988 | *FZD4* | G | A | 0.994 | -0.22426 | 0.0268559 | 3E-17 | 0.0039 | - |
| rs10891479 | 11 | 112,826,303 | *NCAM1* | G | A | 0.818 | 0.030793 | 0.00534703 | 3.3E-09 | 0.0041 | - |
| rs2846901 | 11 | 115,483,879 | *CADM1* | A | G | 0.147 | 0.036748 | 0.00562639 | 1.9E-11 | 0.003 | - |
| rs7959604 | 12 | 1,637,129 | *LOC100292680* | C | G | 0.925 | 0.04829 | 0.00771523 | 1E-10 | 0.0336 | - |
| rs10875906 | 12 | 49,385,679 | *DDN* | C | T | 0.721 | -0.03836 | 0.00457504 | 6.9E-17 | 0.0292 | - |
| rs2761887 | 14 | 54,425,052 | *BMP4* | C | A | 0.452 | -0.03135 | 0.00519416 | 4.6E-09 | 0.3314 | - |
| rs2301522 | 16 | 359,953 | *AXIN1* | A | G | 0.335 | 0.026786 | 0.0043001 | 1.2E-10 | 0.0138 | - |
| rs1381448 | 16 | 51,723,131 | *LOC388276* | G | A | 0.334 | 0.025427 | 0.00425971 | 1.3E-10 | 0.0001 | - |
| rs8064086 | 16 | 67,013,043 | *CES3* | G | C | 0.865 | -0.03472 | 0.00586126 | 7.3E-10 | 0.0279 | - |
| rs11078596 | 17 | 1,618,262 | *C17orf91* | C | T | 0.818 | 0.028464 | 0.00514268 | 2.4E-08 | 0.0002 | - |
| rs117573122 | 17 | 7,388,716 | *POLR2A* | G | C | 0.993 | -0.13659 | 0.0259347 | 5E-09 | 0.0007 | - |
| rs76410205 | 17 | 41,807,508 | *SOST* | C | T | 0.916 | -0.04306 | 0.00781524 | 1E-08 | 0.0935 | - |
| rs8108787 | 19 | 817,980 | *LPPR3* | T | C | 0.738 | 0.033753 | 0.00461693 | 2.1E-12 | 0.0013 | - |
| rs35308216 | 20 | 10,629,525 | *JAG1* | T | C | 0.920 | 0.062004 | 0.00737398 | 6.7E-18 | 0.002 | - |
| rs465004 | 21 | 40,021,851 | *ERG* | T | C | 0.237 | -0.0354 | 0.00474888 | 6E-14 | 0.0078 | - |
| **Tertiary signals reaching GWS by conditional analysis** | | | |  |  |  |  |  |  |  |  |
| rs4654800 | 1 | 22,733,877 | *ZBTB40* | A | G | 0.510 | 0.030 | 0.004 | 5.50E-12 | 0.1493 | 0.0038 |
| rs62159864 | 2 | 119,590,951 | *EN1* | T | A | 0.728 | -0.045 | 0.005 | 3.5E-24 | 0.0011 | 0.0011 |
| rs76865393 | 4 | 998,777 | *IDUA* | C | T | 0.937 | -0.068 | 0.008 | 5.2E-16 | 0.0114 | 0.0028 |
| rs3012465 | 6 | 133,350,936 | *NCRNA00326* | A | G | 0.347 | 0.024 | 0.004 | 2.4E-09 | 0.0571 | 0.0069 |
| rs1890010 | 6 | 152,085,275 | *ESR1* | C | T | 0.290 | -0.031 | 0.004 | 1.40E-12 | 0 | 0.0087 |
| rs1721382 | 7 | 38,104,983 | *STARD3NL* | C | A | 0.348 | -0.04493 | 0.00448697 | 2.60E-24 | 0.1066 | 0.0094 |
| rs1159798 | 10 | 54,412,493 | *MBL2* | A | C | 0.222 | 0.046 | 0.005 | 5.9E-19 | 0.0005 | 0.159 |
| rs17457340 | 20 | 10,640,042 | *JAG1* | T | C | 0.922 | 0.044 | 0.008 | 2.1E-09 | 0.018 | 0.05 |
| rs34778574 | 20 | 11,150,594 | *LOC339593* | T | C | 0.962 | 0.073 | 0.011 | 5.5E-12 | 0.0002 | 0.001 |

**Supplementary Table 3.** Characteristics of 235 independent eBMD-related SNPs used for Mendelian randomization. β is the effect size on eBMD, which is standardized by rank-based inverse normal transformation, per effect allele based on the additive genetic model. PVE was estimated by $\frac{2\beta^{2}EAF(1-EAF)}{2\beta^{2}EAF\left( 1-EAF \right)+{(se(\hat{\beta}))}^{2}2N EAF(1-EAF )}$ N is sample size.

| **SNP** | **Genes Nearest** | **Alleles** | | **EAF** | **Info Score** | **β** | **SE** | **Proportion of**  **variance explained (PVE)** |
| --- | --- | --- | --- | --- | --- | --- | --- | --- |
|  |  | **Effect** | **Other** |  |  |  |  |  |
| rs139603701 | *ACTRT2* | G | A | 0.018 | 0.919 | -0.090 | 0.016 | 0.028% |
| rs2708632 | *RERE* | T | C | 0.343 | 0.998 | -0.032 | 0.004 | 0.049% |
| rs6429787 | *SPEN* | T | C | 0.892 | 0.999 | -0.043 | 0.006 | 0.039% |
| rs12751610 | *ZBTB40* | T | C | 0.825 | 0.997 | -0.068 | 0.005 | 0.144% |
| rs7519889 | *WNT4* | A | G | 0.204 | 0.986 | -0.044 | 0.005 | 0.069% |
| rs4589135 | *ARID1A* | C | T | 0.382 | 0.989 | -0.025 | 0.004 | 0.031% |
| rs10889644 | *SGIP1* | A | C | 0.563 | 0.988 | -0.025 | 0.004 | 0.032% |
| rs11209240 | *WLS* | A | C | 0.839 | 0.977 | -0.048 | 0.005 | 0.067% |
| rs4397637 | *LOC100289178* | G | A | 0.813 | 0.971 | -0.044 | 0.005 | 0.061% |
| rs10922492 | *PKN2* | T | A | 0.615 | 0.992 | -0.026 | 0.004 | 0.034% |
| rs3790608 | *WNT2B* | G | A | 0.849 | 0.986 | -0.046 | 0.006 | 0.058% |
| rs10923715 | *TBX15* | T | A | 0.551 | 0.990 | -0.027 | 0.004 | 0.038% |
| rs7556434 | *NUF2* | A | C | 0.468 | 0.993 | -0.025 | 0.004 | 0.035% |
| rs2421491 | *PRRX1* | T | C | 0.742 | 0.993 | -0.025 | 0.005 | 0.026% |
| rs484686 | *DNM3* | A | G | 0.497 | 0.993 | -0.030 | 0.004 | 0.049% |
| rs6684083 | *DDX59* | G | A | 0.611 | 0.998 | -0.027 | 0.004 | 0.038% |
| rs17514738 | *LOC643723* | C | T | 0.397 | 0.995 | -0.024 | 0.004 | 0.029% |
| rs6663745 | *SLC30A10* | G | T | 0.787 | 0.990 | -0.032 | 0.005 | 0.037% |
| rs4846574 | *RNU5F* | C | T | 0.419 | 0.991 | -0.025 | 0.004 | 0.032% |
| rs7527300 | *C1orf140* | T | C | 0.408 | 0.991 | -0.032 | 0.004 | 0.052% |
| rs1414660 | *FMN2* | C | T | 0.808 | 1.000 | -0.088 | 0.005 | 0.264% |
| rs10192375 | *PLB1* | A | G | 0.510 | 0.991 | -0.023 | 0.004 | 0.028% |
| rs10490046 | *SLC8A1* | C | A | 0.217 | 0.993 | -0.032 | 0.005 | 0.038% |
| rs7576782 | *LOC400950* | C | A | 0.813 | 0.991 | -0.056 | 0.005 | 0.104% |
| rs4305309 | *SPTBN1* | C | T | 0.642 | 0.992 | -0.079 | 0.004 | 0.312% |
| rs3106204 | *SPTBN1* | T | G | 0.296 | 0.997 | -0.044 | 0.004 | 0.088% |
| rs2302643 | *SERTAD2* | A | G | 0.446 | 0.980 | -0.023 | 0.004 | 0.027% |
| rs7578166 | *ZNF638* | A | C | 0.389 | 0.997 | -0.028 | 0.004 | 0.040% |
| rs115242848 | *EN1* | C | T | 0.992 | 0.921 | -0.411 | 0.023 | 0.264% |
| rs4073566 | *INSIG2* | A | C | 0.775 | 0.979 | -0.067 | 0.005 | 0.165% |
| rs62159864 | *EN1* | T | A | 0.728 | 0.986 | -0.054 | 0.004 | 0.122% |
| rs9973853 | *FRZB* | A | G | 0.255 | 0.991 | -0.027 | 0.005 | 0.031% |
| rs4675694 | *FLJ32063* | C | G | 0.173 | 0.973 | -0.037 | 0.005 | 0.041% |
| rs10931982 | *FZD7* | T | C | 0.226 | 1.000 | -0.057 | 0.005 | 0.123% |
| rs35593225 | *CDK15* | C | T | 0.882 | 0.997 | -0.043 | 0.006 | 0.041% |
| rs6741726 | *NBEAL1* | G | A | 0.968 | 0.998 | -0.076 | 0.011 | 0.039% |
| rs11681853 | *DIRC3* | C | G | 0.866 | 0.944 | -0.034 | 0.006 | 0.028% |
| rs2675952 | *NGEF* | T | A | 0.566 | 0.981 | -0.024 | 0.004 | 0.032% |
| rs2606737 | *ATG7* | G | A | 0.816 | 0.992 | -0.034 | 0.005 | 0.036% |
| rs1560633 | *RARB* | T | C | 0.362 | 0.974 | -0.024 | 0.004 | 0.028% |
| rs1599770 | *TRIM71* | C | A | 0.473 | 0.961 | -0.021 | 0.004 | 0.024% |
| rs370387 | *CTNNB1* | G | A | 0.442 | 0.997 | -0.047 | 0.004 | 0.115% |
| rs2526385 | *SEMA3F* | G | T | 0.805 | 1.000 | -0.030 | 0.005 | 0.030% |
| rs4974186 | *ERC2* | G | T | 0.447 | 0.988 | -0.027 | 0.004 | 0.037% |
| rs171080 | *LEKR1* | G | A | 0.121 | 0.996 | -0.049 | 0.006 | 0.056% |
| rs79664023 | *FGFRL1* | G | T | 0.144 | 0.979 | -0.076 | 0.006 | 0.150% |
| rs111632154 | *FGFRL1* | C | T | 0.049 | 0.979 | -0.082 | 0.009 | 0.068% |
| rs76865393 | *IDUA* | C | T | 0.937 | 0.989 | -0.084 | 0.008 | 0.090% |
| rs1386625 | *TBC1D1* | G | A | 0.902 | 0.965 | -0.044 | 0.007 | 0.036% |
| rs11729023 | *SLC4A4* | C | T | 0.878 | 0.980 | -0.036 | 0.006 | 0.029% |
| rs17010961 | *ARHGAP24* | T | A | 0.859 | 0.982 | -0.035 | 0.006 | 0.031% |
| rs1471251 | *AFF1* | A | T | 0.600 | 0.997 | -0.021 | 0.004 | 0.023% |
| rs11934731 | *HSP90AB3P* | A | G | 0.679 | 0.995 | -0.032 | 0.004 | 0.048% |
| rs13137552 | *IBSP* | C | T | 0.506 | 0.977 | -0.031 | 0.004 | 0.052% |
| rs2865339 | *SMARCAD1* | A | G | 0.531 | 0.975 | -0.023 | 0.004 | 0.027% |
| rs62315844 | *C4orf37* | T | C | 0.996 | 0.878 | -0.206 | 0.034 | 0.031% |
| rs6839437 | *OTUD4* | T | C | 0.164 | 1.000 | -0.039 | 0.005 | 0.046% |
| rs35428756 | *PDGFC* | G | GT | 0.392 | 0.986 | -0.023 | 0.004 | 0.027% |
| rs78014439 | *FAT1* | C | G | 0.930 | 0.986 | -0.043 | 0.008 | 0.025% |
| rs6870556 | *CDH6* | A | G | 0.624 | 0.993 | -0.024 | 0.004 | 0.029% |
| rs1428968 | *SLC1A3* | C | T | 0.821 | 0.990 | -0.036 | 0.005 | 0.041% |
| rs2542710 | *DAB2* | G | A | 0.487 | 0.991 | -0.028 | 0.004 | 0.042% |
| rs28744551 | *PPAP2A* | C | G | 0.907 | 0.988 | -0.039 | 0.007 | 0.028% |
| rs9986117 | *RASGRF2* | C | G | 0.686 | 0.995 | -0.025 | 0.004 | 0.029% |
| rs1010109 | *ARHGAP26* | G | A | 0.155 | 0.972 | -0.032 | 0.006 | 0.028% |
| rs368510 | *LOC728264* | G | A | 0.668 | 1.000 | -0.034 | 0.004 | 0.054% |
| rs393952 | *LOC285593* | A | G | 0.521 | 0.996 | -0.021 | 0.004 | 0.024% |
| rs4959677 | *C6orf195* | C | G | 0.490 | 0.993 | -0.026 | 0.004 | 0.037% |
| rs525678 | *RREB1* | G | A | 0.963 | 0.970 | -0.077 | 0.011 | 0.045% |
| rs9379084 | *RREB1* | A | G | 0.117 | 0.938 | -0.049 | 0.006 | 0.051% |
| rs74971894 | *FLJ22536* | G | A | 0.115 | 0.992 | -0.043 | 0.006 | 0.042% |
| rs9260620 | *HLA-A* | T | G | 0.668 | 0.989 | -0.025 | 0.004 | 0.031% |
| rs113166754 | *SUPT3H* | T | C | 0.062 | 0.991 | -0.102 | 0.008 | 0.131% |
| rs72868817 | *BMP5* | A | G | 0.932 | 0.993 | -0.059 | 0.008 | 0.049% |
| rs150445982 | *BMP5* | C | T | 0.975 | 0.839 | -0.099 | 0.014 | 0.044% |
| rs10943125 | *CD109* | T | C | 0.473 | 0.988 | -0.022 | 0.004 | 0.027% |
| rs4526167 | *UBE2CBP* | C | A | 0.699 | 0.993 | -0.029 | 0.004 | 0.037% |
| rs7763784 | *RSPO3* | C | T | 0.520 | 0.999 | -0.083 | 0.004 | 0.367% |
| rs7760760 | *L3MBTL3* | A | G | 0.318 | 0.983 | -0.026 | 0.004 | 0.031% |
| rs9483206 | *EPB41L2* | C | T | 0.377 | 0.996 | -0.031 | 0.004 | 0.048% |
| rs3777787 | *EYA4* | A | C | 0.541 | 0.991 | -0.059 | 0.004 | 0.185% |
| rs547545 | *EYA4* | T | A | 0.540 | 0.991 | -0.036 | 0.004 | 0.068% |
| rs12209685 | *PDE7B* | C | T | 0.620 | 0.984 | -0.027 | 0.004 | 0.035% |
| rs1891002 | *C6orf97* | A | T | 0.286 | 0.997 | -0.100 | 0.004 | 0.440% |
| rs2941740 | *ESR1* | A | G | 0.579 | 1.000 | -0.077 | 0.004 | 0.316% |
| rs1890010 | *ESR1* | C | T | 0.290 | 0.992 | -0.051 | 0.004 | 0.118% |
| rs6903009 | *TIAM2* | A | G | 0.646 | 0.977 | -0.022 | 0.004 | 0.024% |
| rs73029263 | *QKI* | G | A | 0.131 | 0.991 | -0.030 | 0.006 | 0.022% |
| rs2189446 | *MEOX2* | T | C | 0.315 | 0.991 | -0.033 | 0.004 | 0.051% |
| rs3095208 | *ITGB8* | T | C | 0.738 | 0.992 | -0.026 | 0.005 | 0.029% |
| rs85 | *MIR148A* | T | C | 0.198 | 0.996 | -0.043 | 0.005 | 0.063% |
| rs62454420 | *LOC100133311* | A | G | 0.930 | 0.984 | -0.049 | 0.008 | 0.033% |
| rs17501090 | *HOXA11* | A | C | 0.023 | 0.950 | -0.082 | 0.013 | 0.032% |
| rs10244184 | *JAZF1* | C | T | 0.256 | 0.987 | -0.042 | 0.005 | 0.071% |
| rs757980 | *CREB5* | A | G | 0.749 | 0.963 | -0.041 | 0.005 | 0.066% |
| rs10236571 | *AQP1* | G | A | 0.236 | 0.992 | -0.052 | 0.005 | 0.107% |
| rs1717747 | *EPDR1* | A | G | 0.816 | 0.999 | -0.076 | 0.005 | 0.188% |
| rs1052974 | *SFRP4* | C | A | 0.814 | 0.995 | -0.054 | 0.005 | 0.096% |
| rs643044 | *SEMA3E* | A | G | 0.315 | 0.986 | -0.024 | 0.004 | 0.026% |
| rs42038 | *CDK6* | T | C | 0.298 | 0.994 | -0.024 | 0.004 | 0.025% |
| rs7781370 | *FLJ42280* | T | C | 0.336 | 1.000 | -0.047 | 0.004 | 0.106% |
| rs212417 | *ATXN7L1* | A | G | 0.672 | 0.991 | -0.032 | 0.004 | 0.047% |
| rs2707518 | *WNT16* | G | T | 0.607 | 0.979 | -0.170 | 0.004 | 1.451% |
| rs62621812 | *ZNF800* | A | G | 0.022 | 1.000 | -0.092 | 0.014 | 0.040% |
| rs2929308 | *PPP1R3B* | A | T | 0.507 | 0.998 | -0.041 | 0.004 | 0.093% |
| rs6471752 | *EFCAB1* | T | C | 0.152 | 0.987 | -0.033 | 0.006 | 0.030% |
| rs7003794 | *XKR9* | A | C | 0.606 | 0.994 | -0.030 | 0.004 | 0.046% |
| rs114847962 | *EYA1* | T | A | 0.261 | 0.991 | -0.031 | 0.005 | 0.038% |
| rs6999925 | *MRPS28* | G | A | 0.624 | 0.989 | -0.024 | 0.004 | 0.028% |
| rs446027 | *ZBTB10* | G | A | 0.314 | 1.000 | -0.023 | 0.004 | 0.025% |
| rs2737252 | *TRPS1* | G | A | 0.715 | 0.993 | -0.042 | 0.004 | 0.078% |
| rs2205270 | *TRPS1* | T | C | 0.865 | 0.956 | -0.039 | 0.006 | 0.036% |
| rs117108011 | *TNFRSF11B* | A | G | 0.984 | 0.913 | -0.109 | 0.016 | 0.037% |
| rs1487241 | *POU5F1B* | T | A | 0.679 | 0.988 | -0.025 | 0.004 | 0.029% |
| rs6475068 | *BNC2* | G | C | 0.097 | 0.990 | -0.043 | 0.007 | 0.034% |
| rs138945742 | *ADAMTSL1* | T | G | 0.037 | 0.982 | -0.057 | 0.011 | 0.025% |
| rs10992867 | *PHF2* | G | A | 0.747 | 0.994 | -0.030 | 0.005 | 0.037% |
| rs1057713 | *BARX1* | A | G | 0.604 | 0.995 | -0.020 | 0.004 | 0.021% |
| rs1877456 | *PTCH1* | G | C | 0.223 | 0.983 | -0.032 | 0.005 | 0.038% |
| rs10979249 | *KLF4* | T | G | 0.227 | 0.985 | -0.033 | 0.005 | 0.042% |
| rs3802342 | *FUBP3* | G | C | 0.352 | 0.994 | -0.030 | 0.004 | 0.045% |
| rs2519093 | *ABO* | T | C | 0.185 | 0.995 | -0.029 | 0.005 | 0.028% |
| rs74119759 | *PLXDC2* | T | C | 0.159 | 0.964 | -0.034 | 0.006 | 0.032% |
| rs6482632 | *RAB18* | C | T | 0.507 | 0.997 | -0.024 | 0.004 | 0.031% |
| rs12354769 | *MPP7* | T | C | 0.860 | 0.985 | -0.041 | 0.006 | 0.043% |
| rs7919498 | *ZNF438* | C | T | 0.484 | 0.995 | -0.035 | 0.004 | 0.066% |
| rs10824760 | *MBL2* | C | T | 0.108 | 0.978 | -0.138 | 0.006 | 0.390% |
| rs17662822 | *MBL2* | A | C | 0.326 | 0.988 | -0.041 | 0.004 | 0.077% |
| rs1877998 | *KCNMA1* | A | G | 0.182 | 0.997 | -0.034 | 0.005 | 0.038% |
| rs17173698 | *PAPSS2* | G | A | 0.974 | 1.000 | -0.074 | 0.013 | 0.029% |
| rs603424 | *PKD2L1* | A | G | 0.170 | 1.000 | -0.032 | 0.005 | 0.031% |
| rs10885447 | *HABP2* | G | A | 0.789 | 0.983 | -0.030 | 0.005 | 0.032% |
| rs11196170 | *TCF7L2* | A | G | 0.210 | 0.989 | -0.026 | 0.005 | 0.024% |
| rs80226362 | *INPP5A* | G | T | 0.816 | 0.998 | -0.037 | 0.005 | 0.045% |
| rs2653559 | *ST5* | T | C | 0.164 | 0.994 | -0.031 | 0.005 | 0.027% |
| rs61880664 | *INSC* | A | G | 0.898 | 0.977 | -0.044 | 0.007 | 0.037% |
| rs78152188 | *C11orf58* | C | G | 0.712 | 0.994 | -0.032 | 0.004 | 0.044% |
| rs11029901 | *CCDC34* | G | A | 0.641 | 0.991 | -0.044 | 0.004 | 0.094% |
| rs117941208 | *CCDC34* | T | C | 0.111 | 0.970 | -0.043 | 0.006 | 0.038% |
| rs2098878 | *CD44* | A | G | 0.461 | 0.997 | -0.031 | 0.004 | 0.052% |
| rs4579897 | *C11orf49* | T | G | 0.675 | 0.993 | -0.046 | 0.004 | 0.100% |
| rs1530914 | *MS4A4A* | C | T | 0.401 | 0.998 | -0.032 | 0.004 | 0.052% |
| rs174574 | *FADS2* | C | A | 0.646 | 0.996 | -0.024 | 0.004 | 0.029% |
| rs117111740 | *AHNAK* | C | T | 0.027 | 0.960 | -0.133 | 0.012 | 0.097% |
| rs4930295 | *PCNXL3* | G | C | 0.223 | 0.998 | -0.030 | 0.005 | 0.033% |
| rs4988321 | *LRP5* | A | G | 0.051 | 1.000 | -0.080 | 0.009 | 0.066% |
| rs61887821 | *LRP5* | A | G | 0.006 | 0.949 | -0.189 | 0.027 | 0.043% |
| rs1060435 | *TPCN2* | A | G | 0.593 | 0.997 | -0.030 | 0.004 | 0.048% |
| rs649693 | *TMEM135* | C | T | 0.308 | 0.988 | -0.070 | 0.004 | 0.222% |
| rs149504726 | *FZD4* | G | A | 0.994 | 0.850 | -0.190 | 0.027 | 0.043% |
| rs7124639 | *C11orf34* | G | A | 0.599 | 0.995 | -0.054 | 0.004 | 0.149% |
| rs10891479 | *NCAM1* | A | G | 0.182 | 0.929 | -0.034 | 0.005 | 0.034% |
| rs7125361 | *CADM1* | C | G | 0.579 | 0.988 | -0.042 | 0.004 | 0.094% |
| rs2846901 | *CADM1* | G | A | 0.853 | 0.995 | -0.033 | 0.006 | 0.029% |
| rs10790255 | *PHLDB1* | G | T | 0.247 | 0.994 | -0.031 | 0.005 | 0.038% |
| rs1622638 | *MIR100HG* | G | A | 0.607 | 0.996 | -0.022 | 0.004 | 0.025% |
| rs4980826 | *B4GALNT3* | C | A | 0.605 | 1.000 | -0.025 | 0.004 | 0.033% |
| rs9668575 | *LOC100292680* | A | G | 0.745 | 0.950 | -0.037 | 0.005 | 0.053% |
| rs7959604 | *LOC100292680* | G | C | 0.075 | 0.994 | -0.058 | 0.008 | 0.050% |
| rs56151937 | *CACNA1C* | T | G | 0.847 | 0.993 | -0.036 | 0.006 | 0.036% |
| rs117481343 | *EMP1* | C | T | 0.970 | 0.931 | -0.135 | 0.012 | 0.106% |
| rs118115924 | *WNT1* | T | G | 0.012 | 1.000 | -0.176 | 0.018 | 0.078% |
| rs10875906 | *DDN* | C | T | 0.721 | 0.967 | -0.030 | 0.005 | 0.037% |
| rs11832031 | *MSRB3* | A | T | 0.768 | 0.979 | -0.027 | 0.005 | 0.028% |
| rs10858944 | *LOC338758* | G | A | 0.604 | 0.996 | -0.060 | 0.004 | 0.185% |
| rs10859561 | *CRADD* | C | T | 0.487 | 0.989 | -0.023 | 0.004 | 0.027% |
| rs112073168 | *TMEM119* | A | G | 0.028 | 0.960 | -0.065 | 0.012 | 0.023% |
| rs8002850 | *FGF9* | A | G | 0.336 | 1.000 | -0.027 | 0.004 | 0.034% |
| rs1328042 | *SMAD9* | G | A | 0.760 | 0.995 | -0.026 | 0.005 | 0.026% |
| rs8001611 | *AKAP11* | C | T | 0.488 | 0.998 | -0.047 | 0.004 | 0.119% |
| rs770379 | *DLEU7* | G | A | 0.543 | 0.996 | -0.027 | 0.004 | 0.039% |
| rs3812849 | *KLF12* | C | A | 0.261 | 0.991 | -0.027 | 0.005 | 0.031% |
| rs7998877 | *GPC6* | G | T | 0.040 | 0.976 | -0.053 | 0.010 | 0.023% |
| rs2008411 | *DOCK9* | T | C | 0.698 | 0.995 | -0.039 | 0.004 | 0.068% |
| rs1042704 | *MMP14* | A | G | 0.216 | 1.000 | -0.031 | 0.005 | 0.036% |
| rs10145299 | *BAZ1A* | T | C | 0.508 | 0.966 | -0.025 | 0.004 | 0.033% |
| rs4444235 | *BMP4* | T | C | 0.538 | 1.000 | -0.054 | 0.004 | 0.157% |
| rs2588829 | *RAD51L1* | G | A | 0.165 | 0.995 | -0.028 | 0.005 | 0.024% |
| rs56375669 | *FLJ45244* | C | T | 0.087 | 0.992 | -0.043 | 0.007 | 0.031% |
| rs10139279 | *MARK3* | A | G | 0.338 | 0.994 | -0.041 | 0.004 | 0.080% |
| rs11636403 | *CYP19A1* | C | T | 0.542 | 0.970 | -0.035 | 0.004 | 0.062% |
| rs28587205 | *SMAD3* | T | A | 0.477 | 0.993 | -0.025 | 0.004 | 0.032% |
| rs2002122 | *TLE3* | T | G | 0.443 | 0.993 | -0.027 | 0.004 | 0.039% |
| rs11637971 | *CRTC3* | A | C | 0.303 | 1.000 | -0.026 | 0.004 | 0.031% |
| rs34676698 | *AXIN1* | A | G | 0.034 | 0.672 | -0.108 | 0.013 | 0.056% |
| rs2301522 | *AXIN1* | G | A | 0.665 | 0.991 | -0.032 | 0.004 | 0.047% |
| rs6497636 | *USP31* | G | A | 0.322 | 0.960 | -0.025 | 0.004 | 0.027% |
| rs72805220 | *LOC388276* | A | C | 0.066 | 0.949 | -0.065 | 0.008 | 0.053% |
| rs1381448 | *LOC388276* | A | G | 0.666 | 0.996 | -0.029 | 0.004 | 0.040% |
| rs17680862 | *PLEKHG4* | C | G | 0.026 | 1.000 | -0.085 | 0.012 | 0.040% |
| rs8064086 | *CES3* | G | C | 0.865 | 0.992 | -0.035 | 0.006 | 0.030% |
| rs4888151 | *CMIP* | C | A | 0.743 | 0.971 | -0.027 | 0.005 | 0.028% |
| rs71390846 | *FOXL1* | C | G | 0.190 | 0.984 | -0.041 | 0.005 | 0.056% |
| rs113478686 | *FAM38A* | CT | C | 0.227 | 0.992 | -0.025 | 0.005 | 0.023% |
| rs2663339 | *ABR* | C | A | 0.308 | 0.989 | -0.027 | 0.004 | 0.035% |
| rs8072532 | *SMG6* | G | A | 0.302 | 0.995 | -0.055 | 0.004 | 0.139% |
| rs11078596 | *C17orf91* | T | C | 0.182 | 1.000 | -0.029 | 0.005 | 0.028% |
| rs74439044 | *CHD3* | C | T | 0.096 | 0.994 | -0.044 | 0.007 | 0.035% |
| rs117573122 | *POLR2A* | G | C | 0.993 | 0.861 | -0.140 | 0.026 | 0.025% |
| rs77152265 | *NTN1* | C | T | 0.159 | 0.964 | -0.035 | 0.006 | 0.034% |
| rs3760456 | *CORO6* | T | C | 0.441 | 0.996 | -0.034 | 0.004 | 0.061% |
| rs7215205 | *RAB11FIP4* | C | T | 0.633 | 0.985 | -0.024 | 0.004 | 0.029% |
| rs143043662 | *JUP* | C | T | 0.986 | 1.000 | -0.092 | 0.017 | 0.024% |
| rs7209826 | *SOST* | A | G | 0.619 | 0.973 | -0.044 | 0.004 | 0.096% |
| rs2732581 | *KIAA1267* | G | A | 0.238 | 0.972 | -0.023 | 0.005 | 0.020% |
| rs2696264 | *TMEM92* | G | A | 0.771 | 1.000 | -0.031 | 0.005 | 0.037% |
| rs72829754 | *ANKFN1* | G | A | 0.602 | 0.986 | -0.036 | 0.004 | 0.067% |
| rs1036902 | *BCAS3* | T | C | 0.853 | 0.997 | -0.051 | 0.006 | 0.070% |
| rs11869530 | *AXIN2* | G | A | 0.363 | 1.000 | -0.028 | 0.004 | 0.039% |
| rs73997493 | *SEPT9* | T | C | 0.087 | 0.988 | -0.047 | 0.007 | 0.037% |
| rs12150031 | *BAHCC1* | G | C | 0.390 | 1.000 | -0.024 | 0.004 | 0.030% |
| rs11875132 | *APCDD1* | C | T | 0.435 | 0.980 | -0.025 | 0.004 | 0.032% |
| rs4430817 | *C18orf19* | C | G | 0.357 | 0.995 | -0.049 | 0.004 | 0.118% |
| rs12967019 | *SMAD7* | T | C | 0.509 | 0.954 | -0.023 | 0.004 | 0.028% |
| rs2957137 | *TNFRSF11A* | A | T | 0.360 | 0.984 | -0.023 | 0.004 | 0.025% |
| rs657693 | *NFATC1* | A | G | 0.637 | 1.000 | -0.022 | 0.004 | 0.025% |
| rs11084888 | *SBNO2* | A | G | 0.311 | 0.993 | -0.037 | 0.004 | 0.064% |
| rs8108787 | *LPPR3* | C | T | 0.262 | 0.965 | -0.033 | 0.005 | 0.043% |
| rs12609327 | *DOT1L* | C | A | 0.500 | 0.992 | -0.024 | 0.004 | 0.032% |
| rs60507951 | *RHPN2* | G | A | 0.908 | 0.987 | -0.111 | 0.007 | 0.220% |
| rs28364580 | *AXL* | A | G | 0.248 | 0.964 | -0.026 | 0.005 | 0.025% |
| rs3170167 | *FOSB* | C | T | 0.150 | 0.988 | -0.036 | 0.006 | 0.035% |
| rs71338564 | *FERMT1* | G | A | 0.647 | 0.977 | -0.035 | 0.004 | 0.058% |
| rs6117854 | *HAO1* | A | G | 0.329 | 0.977 | -0.038 | 0.004 | 0.065% |
| rs6040286 | *LOC339593* | T | C | 0.438 | 0.996 | -0.041 | 0.004 | 0.087% |
| rs35308216 | *JAG1* | C | T | 0.080 | 1.000 | -0.065 | 0.007 | 0.068% |
| rs34778574 | *LOC339593* | C | T | 0.038 | 1.000 | -0.073 | 0.011 | 0.042% |
| rs13044413 | *ITCH* | A | G | 0.560 | 0.997 | -0.023 | 0.004 | 0.028% |
| rs6016547 | *LPIN3* | C | A | 0.164 | 0.988 | -0.029 | 0.005 | 0.024% |
| rs1150442 | *EYA2* | T | C | 0.256 | 0.980 | -0.032 | 0.005 | 0.041% |
| rs2830907 | *NCRNA00113* | G | A | 0.537 | 0.998 | -0.031 | 0.004 | 0.051% |
| rs9982895 | *ETS2* | C | T | 0.727 | 0.993 | -0.045 | 0.004 | 0.086% |
| rs465004 | *ERG* | T | C | 0.237 | 0.983 | -0.035 | 0.005 | 0.046% |
| rs9606139 | *SEPT5* | A | G | 0.110 | 0.947 | -0.117 | 0.007 | 0.274% |
| rs2049939 | *KREMEN1* | C | G | 0.650 | 0.998 | -0.042 | 0.004 | 0.086% |

**Supplementary Table 4:** Association analysis of previously reported genome-wide significant hits for different bone mineral density (BMD) measurements. β is the effect size on DXA BMD (lead trait) ^a^ or eBMD ^b^, per effect allele based on the additive genetic model. Direction “+” means that the SNP shows the same direction of association with the BMD trait in both UKB and the previous study.

| **Gene** | **SNP** | **Chr** | **Position** | **Locus** | **Effect Allele** | **Other Allele** | **Effect allele**  **frequency** | **Previous Studies** | | | |  | **UK Biobank (eBMD)** | | **Direction** |
| --- | --- | --- | --- | --- | --- | --- | --- | --- | --- | --- | --- | --- | --- | --- | --- |
|  |  |  |  |  |  |  |  | **β ^a^** | ***P*** | **Lead Trait** | **Reference (PMID)** |  | **β ^b^** | ***P*** |  |
| ***WNT4*** | rs7521902 | 1 | 22490724 | 1p36.12 | A | C | 0.31 | -0.05 | 9.66E-11 | LSBMD | 22504420 |  | -0.037 | 3.8E-17 | + |
| ***ZBTB40*** | rs6426749 | 1 | 22711473 | 1p36.12 | C | G | 0.17 | 0.11 | 7.39E-57 | FNBMD | 22504420 |  | 0.067 | 3.9E-40 | + |
| ***LOC100289178*** | rs1430742 | 1 | 68635075 | 1p31.3 | T | C | 0.79 | -0.105 | 2.6E-13 | FNBMD | 19801982 |  | -0.027 | 2.8E-08 | + |
|  | rs12407028 | 1 | 68647716 | 1p31.3 | T | C | 0.6 | 0.08 | 3.11E-45 | LSBMD | 22504420 |  | 0.028 | 3.9E-12 | + |
|  | rs17482952 | 1 | 69105068 | 1p31.3 | A | G | 0.93 | 0.08 | 1.31E-11 | FNBMD | 22504420 |  | 0.014 | 0.078 | + |
| ***DNM3*** | rs479336 | 1 | 172199573 | 1q24.3 | T | G | 0.74 | -0.04 | 8.51E-15 | FNBMD | 22504420 |  | -0.027 | 2.2E-09 | + |
| ***FMN2*** | rs9287237 | 1 | 240597214 | 1q43 | T | G | 0.15 | 0.22 | 3.3E-08 | Trabecular vBMD | 23437003 |  | 0.087 | 8.7E-63 | + |
| ***PKDCC*** | rs7584262 | 2 | 42250549 | 2p21 | T | C | 0.23 | 0.04 | 1.27E-09 | FNBMD | 22504420 |  | 0.052 | 9.1E-28 | + |
| ***SPTBN1*** | rs4233949 | 2 | 54659707 | 2p16.2 | C | G | 0.38 | 0.05 | 2.25E-18 | LSBMD | 22504420 |  | 0.078 | 1.3E-87 | + |
|  | rs11898505 | 2 | 54684557 | 2p16.2 | G | A | 0.64 | -0.06 | 3.7E-18 | BUA | 24430505 |  | -0.079 | 5.9E-87 | + |
| ***ANAPC1*** | rs17040773 | 2 | 112500035 | 2q13 | A | C | 0.76 | 0.04 | 1.51E-09 | FNBMD | 22504420 |  | 0.007 | 0.092 | + |
| ***INSIG2*** | rs1878526 | 2 | 119038598 | 2q14.2 | A | G | 0.22 | 0.04 | 1.22E-10 | LSBMD | 22504420 |  | 0.065 | 4.8E-44 | + |
| ***EN1*** | rs11692564 | 2 | 119545994 | 2q14.2 | T | C | 0.016 | 0.22 | 1.7E-14 | LSBMD | 26367794 |  | 0.237 | 2.3E-52 | + |
| ***GALNT3*** | rs1346004 | 2 | 166601046 | 2q24.3 | A | G | 0.5 | -0.06 | 3.87E-30 | LSBMD | 22504420 |  | -0.015 | 0.00026 | + |
| ***CTNNB1*** | rs430727 | 3 | 41128564 | 3p22.1 | T | C | 0.48 | -0.06 | 4.41E-25 | FNBMD | 22504420 |  | -0.045 | 8E-33 | + |
| ***KIAA2018*** | rs1026364 | 3 | 113370010 | 3q13.2 | T | G | 0.37 | 0.03 | 4.08E-10 | FNBMD | 22504420 |  | 0.013 | 9E-04 | + |
| ***LEKR1*** | rs344081 | 3 | 156555984 | 3q25.31 | T | C | 0.87 | 0.06 | 4.46E-12 | LSBMD | 22504420 |  | 0.049 | 5.9E-18 | + |
| ***IDUA*** | rs3755955 | 4 | 994414 | 4p16.3 | A | G | 0.16 | -0.06 | 5.24E-15 | LSBMD | 22504420 |  | -0.071 | 4.8E-37 | + |
| ***MEPE*** | rs6532023 | 4 | 88773849 | 4q22.1 | T | G | 0.34 | 0.06 | 1.23E-27 | LSBMD | 22504420 |  | 0.030 | 3E-14 | + |
| ***MEF2C*** | rs11951031 | 5 | 88138731 | 5q14.3 | A | G | 0.06 | -0.2 | 9E-08 | FNBMD | 23572186 |  | -0.005 | 0.76 | + |
| ***MEF2C*** | rs1366594 | 5 | 88376061 | 5q14.3 | A | C | 0.54 | -0.08 | 4.47E-61 | FNBMD | 22504420 |  | -0.002 | 0.67 | + |
| ***CDKAL1*** | rs9466056 | 6 | 21384613 | 6p22.3 | A | G | 0.38 | -0.04 | 2.73E-13 | FNBMD | 22504420 |  | -0.015 | 5.5E-05 | + |
| ***SUPT3H*** | rs11755164 | 6 | 44639184 | 6p21.1 | T | C | 0.4 | -0.04 | 5.6E-11 | LSBMD | 22504420 |  | -0.024 | 4E-09 | + |
| ***RSPO3*** | rs13204965 | 6 | 127167072 | 6q22.32 | A | C | 0.76 | -0.04 | 8.12E-12 | FNBMD | 22504420 |  | -0.016 | 4.5E-04 | + |
|  | rs7741021 | 6 | 127468274 | 6q22.33 | C | A | 0.47 | 0.06 | 3.5E-23 | BUA | 24430505 |  | 0.082 | 2.1E-99 | + |
| ***LINC00326*** | rs271170 | 6 | 133315804 | 6q23.2 | T | C | 0.33 | -0.11 | 2.7E-12 | Cortical vBMD | 23437003 |  | 0.036 | 3.8E-19 | - |
|  | rs3012465 | 6 | 133350936 | 2q14.2 | G | A | 0.65 | 0.127 | 8.29E-17 | Skull BMD | 24945404 |  | -0.038 | 3.9E-21 | - |
| ***C6orf97*** | rs6909279 | 6 | 151895456 | 6q25.1 | G | C | 0.4 | -0.09 | 1.1E-09 | Cortical vBMD | 23437003 |  | -0.082 | 8.3E-96 | + |
| ***ESR1*** | rs3020331 | 6 | 152008780 | 6q25.1 | T | C | 0.43 | 0.05 | 4.8E-18 | BUA | 24430505 |  | 0.077 | 2.1E-83 | + |
|  | rs2982552 | 6 | 152059563 | 6q25.1 | G | A | 0.46 | -0.05 | 3.8E-15 | BUA | 24430505 |  | -0.066 | 1.1E-64 | + |
| ***TXNDC3*** | rs10226308 | 7 | 37938422 | 7p14.1 | A | G | 0.84 | -0.06 | 6.4E-13 | LSBMD | 22504420 |  | -0.053 | 1.7E-26 | + |
| ***STARD3NL*** | rs6959212 | 7 | 38128326 | 7p14.1 | T | C | 0.32 | -0.07 | 3.76E-38 | LSBMD | 22504420 |  | -0.055 | 1.2E-40 | + |
| ***COL1A2*** | chr7:93879914 | 7 | 93509226 | 7q21.3 | C | G | 0.11 | 1.53 | 1.8E-07 | low BMD | 26235824 |  | NA | NA | NA |
|  | chr7:93887508 | 7 | 93516820 | 7q21.3 | A | G | 0.07 | 2.23 | 1.9E-08 | low BMD | 26235824 |  | NA | NA | NA |
| ***FLJ42280*** | rs4727338 | 7 | 96120675 | 7q21.3 | C | G | 0.67 | 0.08 | 8.1E-48 | FNBMD | 22504420 |  | 0.047 | 3.1E-30 | + |
| ***NRCAM*** | rs6975557 | 7 | 107973297 | 7q31.1 | G | A | 0.16 | 0.16 | 4.8E-08 | FNBMD | 23593202 |  | 0.008 | 0.026 | + |
| ***C7orf58*** | rs13245690 | 7 | 120785064 | 7q31.31 | A | G | 0.65 | -0.05 | 1.65E-11 | LSBMD | 22504420 |  | -0.063 | 1.9E-54 | + |
| ***WNT16*** | rs2908007 | 7 | 120962164 | 7q31.31 | G | A | 0.4 | 0.15 | 5.6E-111 | BUA | 24430505 |  | 0.171 | 0 | + |
|  | rs3801387 | 7 | 120974765 | 7q31.31 | A | G | 0.74 | -0.09 | 3.17E-51 | LSBMD | 22504420 |  | -0.166 | 1.7E-316 | + |
| ***ABCF2*** | rs7812088 | 7 | 150919829 | 7q36.1 | A | G | 0.13 | -0.05 | 7.28E-09 | FNBMD | 22504420 |  | -0.014 | 0.025 | + |
| ***XKR9*** | rs7017914 | 8 | 71591203 | 8q13.3 | A | G | 0.49 | 0.03 | 2.29E-07 | FNBMD | 22504420 |  | 0.026 | 3.9E-12 | + |
| ***TNFRSF11B*** | rs7839059 | 8 | 119976542 | 8q24.12 | A | C | 0.34 | -0.1 | 4.1E-09 | Cortical vBMD | 23437003 |  | -0.010 | 0.034 | + |
|  | rs2062377 | 8 | 120007420 | 8q24.12 | A | T | 0.57 | -0.08 | 3.16E-39 | LSBMD | 22504420 |  | -0.012 | 0.005 | + |
| ***LOC554202*** | rs7035284 | 9 | 21678973 | 9p21.3 | G | A | 0.279 | 0.277 | 1.21E-08 | FNBMD | 26041818 |  | 0.003 | 0.43 | + |
| ***FUBP3*** | rs7851693 | 9 | 133478827 | 9q34.11 | C | G | 0.64 | 0.05 | 3.37E-22 | FNBMD | 22504420 |  | 0.030 | 1.1E-12 | + |
| ***MPP7*** | rs3905706 | 10 | 28479942 | 10p11.23 | T | C | 0.22 | 0.05 | 2.41E-16 | LSBMD | 22504420 |  | -0.026 | 5.4E-08 | - |
| ***MBL2*** | rs1373004 | 10 | 54427825 | 10q21.1 | T | G | 0.13 | -0.06 | 1.56E-12 | LSBMD | 22504420 |  | -0.13711 | 5.8E-108 | + |
| ***KCNMA1*** | rs7071206 | 10 | 79401316 | 10q22.3 | T | C | 0.78 | -0.06 | 5.02E-19 | LSBMD | 22504420 |  | 0.029 | 7.3E-09 | - |
| ***CPN1*** | rs7084921 | 10 | 101813802 | 10q24.2 | T | C | 0.39 | 0.03 | 9.03E-10 | FNBMD | 22504420 |  | 0.009 | 0.016 | + |
| ***SOX6*** | rs7108738 | 11 | 15710084 | 11p15.2 | T | G | 0.83 | -0.08 | 1.08E-32 | FNBMD | 22504420 |  | -0.015 | 0.003 | + |
| ***LIN7C*** | rs10835187 | 11 | 27505677 | 11p14.1 | T | C | 0.55 | -0.03 | 4.9E-08 | LSBMD | 22504420 |  | -0.026 | 6.1E-10 | + |
| ***DCDC5*** | rs163879 | 11 | 30951674 | 11p14.1 | T | C | 0.68 | -0.04 | 2.19E-11 | LSBMD | 22504420 |  | -0.010 | 0.006 | + |
| ***ZNF408*** | rs7932354 | 11 | 46722221 | 11p11.2 | T | C | 0.31 | 0.05 | 5.12E-18 | FNBMD | 22504420 |  | 0.040 | 4.7E-20 | + |
| ***LRP5*** | rs3736228 | 11 | 68201295 | 11q13.2 | T | C | 0.16 | -0.08 | 2.08E-26 | LSBMD | 22504420 |  | -0.040 | 1.9E-14 | + |
| ***TMEM135*** | rs597319 | 11 | 86853997 | 11q14.2 | G | A | 0.31 | -0.06 | 7.6E-18 | BUA | 24430505 |  | -0.066 | 3.1E-55 | + |
| ***LOC100292680*** | rs2887571 | 12 | 1638171 | 12p13.33 | A | G | 0.76 | -0.04 | 5.59E-12 | LSBMD | 22504420 |  | -0.036 | 3.1E-15 | + |
| ***KLHDC5*** | rs7953528 | 12 | 28017159 | 12p11.22 | A | T | 0.18 | 0.05 | 1.87E-12 | FNBMD | 22504420 |  | 0.005 | 0.38 | + |
| ***DHH*** | rs12821008 | 12 | 49474605 | 12q13.12 | T | C | 0.39 | 0.05 | 1.17E-15 | LSBMD | 22504420 |  | 0.019 | 1.1E-05 | + |
| ***SP7*** | rs2016266 | 12 | 53727955 | 12q13.13 | A | G | 0.68 | -0.05 | 2.95E-20 | LSBMD | 22504420 |  | -0.008 | 0.059 | + |
| ***HOXC5*** | rs736825 | 12 | 54417576 | 12q13.13 | C | G | 0.56 | 0.05 | 7.68E-16 | LSBMD | 22504420 |  | 0.005 | 0.23 | + |
| ***C12orf23*** | rs1053051 | 12 | 107367225 | 12q23.3 | T | C | 0.52 | 0.03 | 9.6E-10 | FNBMD | 22504420 |  | 0.016 | 1.5E-04 | + |
| ***AKAP11-TNFSF11*** | rs9533090 | 13 | 42951449 | 13q14.11 | T | C | 0.49 | -0.1 | 4.82E-68 | LSBMD | 22504420 |  | -0.047 | 6.9E-35 | + |
|  | rs1021188 | 13 | 43116133 | 13q14.11 | C | T | 0.17 | -0.15 | 1.4E-12 | Cortical vBMD | 23437003 |  | -0.012 | 7.1E-03 | + |
| ***RPS6KA5*** | rs1286083 | 14 | 91442779 | 14q32.12 | T | C | 0.81 | -0.05 | 2.02E-15 | FNBMD | 22504420 |  | -0.021 | 1.9E-05 | + |
| ***MARK3*** | rs11623869 | 14 | 103883633 | 14q32.32 | T | G | 0.35 | -0.04 | 5.2E-16 | FNBMD | 22504420 |  | -0.040 | 2.8E-23 | + |
| ***AXIN1*** | rs9921222 | 16 | 375782 | 16p13.3 | T | C | 0.48 | -0.04 | 1E-16 | LSBMD | 22504420 |  | -0.028 | 2.2E-12 | + |
| ***PTX4*** | rs13336428 | 16 | 1532463 | 16p13.3 | A | G | 0.43 | -0.04 | 1.49E-16 | FNBMD | 22504420 |  | -0.014 | 6.9E-04 | + |
| ***PDXDC1*** | rs4985155 | 16 | 15129459 | 16p13.11 | A | G | 0.67 | -0.03 | 1.74E-10 | FNBMD | 22504420 |  | -0.003 | 0.5 | + |
| ***CYLD-SALL1*** | rs1564981 | 16 | 50986308 | 16q12.1 | A | G | 0.5 | -0.04 | 1.95E-10 | LSBMD | 22504420 |  | -0.015 | 9.8E-05 | + |
|  | rs1566045 | 16 | 51021803 | 16q12.1 | T | C | 0.8 | -0.06 | 1.94E-22 | FNBMD | 22504420 |  | -0.047 | 1.1E-07 | + |
| ***FOXL1*** | rs10048146 | 16 | 86710660 | 16q24.1 | A | G | 0.8 | 0.05 | 1E-14 | FNBMD | 22504420 |  | 0.038 | 4E-14 | + |
| ***SMG6*** | rs4790881 | 17 | 2068932 | 17p13.3 | A | C | 0.69 | 0.05 | 9.75E-19 | FNBMD | 22504420 |  | 0.055 | 5.8E-39 | + |
| ***SOST-C17orf53*** | rs4792909 | 17 | 41798824 | 17q21.31 | T | G | 0.37 | 0.04 | 1.95E-11 | FNBMD | 22504420 |  | 0.044 | 3.2E-28 | + |
|  | rs227584 | 17 | 42225547 | 17q21.31 | A | C | 0.7 | -0.06 | 2.56E-24 | FNBMD | 22504420 |  | -0.026 | 4.1E-09 | + |
| ***MAPT*** | rs1864325 | 17 | 43977827 | 17q21.31 | T | C | 0.22 | -0.04 | 4.89E-11 | LSBMD | 22504420 |  | -0.021 | 3.7E-07 | + |
| ***SOX9*** | rs7217932 | 17 | 69949016 | 17q24.3 | A | G | 0.46 | 0.03 | 1.92E-11 | FNBMD | 22504420 |  | 0.017 | 1E-05 | + |
| ***C18orf19*** | rs4796995 | 18 | 13708574 | 18p11.21 | A | G | 0.63 | 0.03 | 4.85E-08 | FNBMD | 22504420 |  | 0.049 | 5.8E-32 | + |
| ***TNFRSF11A*** | rs884205 | 18 | 60054857 | 18q21.33 | A | C | 0.27 | -0.05 | 1.58E-17 | LSBMD | 22504420 |  | -0.023 | 4.1E-09 | + |
| ***GPATCH1*** | rs10416265 | 19 | 33605300 | 19q13.11 | G | A | 0.27 | 0.06 | 2.4E-13 | LSBMD | 24430505 |  | 0.058 | 5.2E-40 | + |
| ***JAG1*** | rs3790160 | 20 | 10639988 | 20p12.2 | T | C | 0.5 | 0.05 | 3.07E-19 | LSBMD | 22504420 |  | 0.030 | 3.6E-15 | + |
| ***FAM9B*** | rs5934507 | 23 | 8885247 | Xp22.31 | A | G | 0.73 | -0.06 | 1.18E‐08 | LSBMD | 22504420 |  | NA | NA | NA |

LSBMD, lumbar spine BMD; FNBMD, femoral neck BMD; vBMD, volumetric BMD; BUA, broadband ultrasound attenuation

**Supplementary Table 5.** Comparison of the effect estimates of eBMD related SNPs stratified by two GWAS arrays in UKBB: UK BiLEVE array (n= 40,476) and UK Biobank Array (n= 76,025). β is the effect size on eBMD, which is standardized by rank-based inverse normal transformation, per effect allele based on the additive genetic model.

| **SNP** | **Chr.** | **Position (bp)** | **Genes** | **Alleles** | |  | **UK BiLEVE Array** | |  | **UK Biobank Array** | | ***P* for heterogeneity** |
| --- | --- | --- | --- | --- | --- | --- | --- | --- | --- | --- | --- | --- |
|  |  |  | **Nearest** | **Effect** | **Other** |  | **β** | **SE** |  | **β** | **SE** |  |
| rs139603701 | 1 | 2,904,634 | *ACTRT2* | A | G |  | 0.0646 | 0.0273 |  | 0.1027 | 0.0196 | 2.57E-01 |
| rs2708632 | 1 | 8,464,509 | *RERE* | T | C |  | -0.0318 | 0.0073 |  | -0.0336 | 0.0052 | 8.47E-01 |
| rs6429787 | 1 | 16,230,657 | *SPEN* | T | C |  | -0.0370 | 0.0111 |  | -0.0500 | 0.0080 | 3.46E-01 |
| rs7519889 | 1 | 22,472,506 | *WNT4* | G | A |  | 0.0436 | 0.0087 |  | 0.0461 | 0.0062 | 8.11E-01 |
| rs12751610 | 1 | 22,701,761 | *ZBTB40* | T | C |  | -0.0597 | 0.0091 |  | -0.0713 | 0.0065 | 2.99E-01 |
| rs4589135 | 1 | 27,041,714 | *ARID1A* | T | C |  | 0.0337 | 0.0071 |  | 0.0192 | 0.0051 | 9.86E-02 |
| rs10889644 | 1 | 67,145,322 | *SGIP1* | C | A |  | 0.0249 | 0.0070 |  | 0.0248 | 0.0050 | 1.00E+00 |
| rs4397637 | 1 | 68,603,131 | *LOC100289178* | G | A |  | -0.0335 | 0.0090 |  | -0.0478 | 0.0065 | 1.96E-01 |
| rs11209240 | 1 | 68,725,907 | *WLS* | A | C |  | -0.0642 | 0.0095 |  | -0.0398 | 0.0068 | 3.72E-02 |
| rs10922492 | 1 | 89,304,059 | *PKN2* | T | A |  | -0.0238 | 0.0071 |  | -0.0279 | 0.0051 | 6.45E-01 |
| rs3790608 | 1 | 113,055,023 | *WNT2B* | G | A |  | -0.0553 | 0.0096 |  | -0.0415 | 0.0070 | 2.45E-01 |
| rs10923715 | 1 | 119,535,334 | *TBX15* | T | A |  | -0.0216 | 0.0070 |  | -0.0301 | 0.0050 | 3.23E-01 |
| rs7556434 | 1 | 163,883,114 | *NUF2* | C | A |  | 0.0264 | 0.0070 |  | 0.0242 | 0.0050 | 8.00E-01 |
| rs2421491 | 1 | 170,693,914 | *PRRX1* | T | C |  | -0.0282 | 0.0079 |  | -0.0271 | 0.0057 | 9.12E-01 |
| rs484686 | 1 | 172,152,202 | *DNM3* | A | G |  | -0.0301 | 0.0069 |  | -0.0311 | 0.0050 | 9.07E-01 |
| rs6684083 | 1 | 200,673,094 | *DDX59* | A | G |  | 0.0250 | 0.0071 |  | 0.0285 | 0.0051 | 6.92E-01 |
| rs17514738 | 1 | 218,988,754 | *LOC643723* | T | C |  | 0.0243 | 0.0071 |  | 0.0228 | 0.0051 | 8.60E-01 |
| rs4846574 | 1 | 219,846,938 | *RNU5F* | C | T |  | -0.0221 | 0.0070 |  | -0.0235 | 0.0051 | 8.74E-01 |
| rs6663745 | 1 | 220,097,906 | *SLC30A10* | T | G |  | 0.0208 | 0.0085 |  | 0.0386 | 0.0061 | 8.75E-02 |
| rs7527300 | 1 | 221,477,744 | *C1orf140* | C | T |  | 0.0342 | 0.0071 |  | 0.0300 | 0.0051 | 6.25E-01 |
| rs1414660 | 1 | 240,586,695 | *FMN2* | C | T |  | -0.0914 | 0.0088 |  | -0.0859 | 0.0063 | 6.11E-01 |
| rs10192375 | 2 | 28,892,116 | *PLB1* | A | G |  | -0.0273 | 0.0069 |  | -0.0209 | 0.0050 | 4.54E-01 |
| rs10490046 | 2 | 40,630,678 | *SLC8A1* | A | C |  | 0.0224 | 0.0084 |  | 0.0382 | 0.0060 | 1.26E-01 |
| rs7576782 | 2 | 42,218,378 | *LOC400950* | C | A |  | -0.0551 | 0.0089 |  | -0.0571 | 0.0064 | 8.55E-01 |
| rs4305309 | 2 | 54,683,711 | *SPTBN1* | T | C |  | 0.0884 | 0.0072 |  | 0.0743 | 0.0052 | 1.13E-01 |
| rs3106204 | 2 | 54,863,352 | *SPTBN1* | T | G |  | -0.0466 | 0.0076 |  | -0.0411 | 0.0055 | 5.54E-01 |
| rs2302643 | 2 | 64,881,229 | *SERTAD2* | G | A |  | 0.0192 | 0.0070 |  | 0.0249 | 0.0050 | 5.06E-01 |
| rs7578166 | 2 | 71,630,041 | *ZNF638* | A | C |  | -0.0280 | 0.0071 |  | -0.0288 | 0.0051 | 9.26E-01 |
| rs4073566 | 2 | 119,161,638 | *INSIG2* | C | A |  | 0.0671 | 0.0083 |  | 0.0650 | 0.0060 | 8.41E-01 |
| rs115242848 | 2 | 119,507,607 | *EN1* | C | T |  | -0.4498 | 0.0406 |  | -0.3895 | 0.0292 | 2.27E-01 |
| rs62159864 | 2 | 119,590,951 | *EN1* | T | A |  | -0.0562 | 0.0078 |  | -0.0520 | 0.0056 | 6.63E-01 |
| rs9973853 | 2 | 183,738,444 | *FRZB* | G | A |  | 0.0329 | 0.0079 |  | 0.0246 | 0.0057 | 3.95E-01 |
| rs4675694 | 2 | 200,450,012 | *FLJ32063* | C | G |  | -0.0384 | 0.0093 |  | -0.0348 | 0.0067 | 7.54E-01 |
| rs35593225 | 2 | 202,792,094 | *CDK15* | C | T |  | -0.0436 | 0.0107 |  | -0.0396 | 0.0077 | 7.58E-01 |
| rs10931982 | 2 | 202,832,130 | *FZD7* | T | C |  | -0.0473 | 0.0083 |  | -0.0613 | 0.0059 | 1.68E-01 |
| rs6741726 | 2 | 203,965,530 | *NBEAL1* | A | G |  | 0.0834 | 0.0196 |  | 0.0725 | 0.0141 | 6.52E-01 |
| rs11681853 | 2 | 218,063,297 | *DIRC3* | C | G |  | -0.0334 | 0.0104 |  | -0.0345 | 0.0075 | 9.32E-01 |
| rs2675952 | 2 | 233,790,522 | *NGEF* | T | A |  | -0.0266 | 0.0070 |  | -0.0237 | 0.0051 | 7.37E-01 |
| rs2606737 | 3 | 11,398,654 | *ATG7* | A | G |  | 0.0341 | 0.0089 |  | 0.0319 | 0.0065 | 8.37E-01 |
| rs1560633 | 3 | 25,548,555 | *RARB* | T | C |  | -0.0165 | 0.0073 |  | -0.0259 | 0.0052 | 2.95E-01 |
| rs1599770 | 3 | 32,924,374 | *TRIM71* | A | C |  | 0.0204 | 0.0070 |  | 0.0219 | 0.0051 | 8.62E-01 |
| rs370387 | 3 | 41,123,984 | *CTNNB1* | G | A |  | -0.0432 | 0.0069 |  | -0.0473 | 0.0050 | 6.29E-01 |
| rs2526385 | 3 | 50,181,135 | *SEMA3F* | T | G |  | 0.0243 | 0.0088 |  | 0.0310 | 0.0063 | 5.30E-01 |
| rs4974186 | 3 | 56,263,534 | *ERC2* | G | T |  | -0.0366 | 0.0070 |  | -0.0213 | 0.0050 | 7.64E-02 |
| rs171080 | 3 | 156,555,500 | *LEKR1* | A | G |  | 0.0493 | 0.0106 |  | 0.0499 | 0.0076 | 9.63E-01 |
| rs76865393 | 4 | 998,777 | *IDUA* | C | T |  | -0.0730 | 0.0145 |  | -0.0910 | 0.0102 | 3.09E-01 |
| rs79664023 | 4 | 1,004,863 | *FGFRL1* | T | G |  | 0.0749 | 0.0099 |  | 0.0758 | 0.0071 | 9.46E-01 |
| rs111632154 | 4 | 1,012,300 | *FGFRL1* | T | C |  | 0.1054 | 0.0158 |  | 0.0705 | 0.0117 | 7.53E-02 |
| rs1386625 | 4 | 38,361,120 | *TBC1D1* | A | G |  | 0.0402 | 0.0118 |  | 0.0464 | 0.0085 | 6.72E-01 |
| rs11729023 | 4 | 71,991,184 | *SLC4A4* | C | T |  | -0.0260 | 0.0107 |  | -0.0388 | 0.0077 | 3.32E-01 |
| rs17010961 | 4 | 86,723,103 | *ARHGAP24* | T | A |  | -0.0359 | 0.0100 |  | -0.0365 | 0.0072 | 9.64E-01 |
| rs1471251 | 4 | 87,976,359 | *AFF1* | A | T |  | -0.0150 | 0.0070 |  | -0.0251 | 0.0051 | 2.44E-01 |
| rs13137552 | 4 | 88,715,324 | *IBSP* | T | C |  | 0.0269 | 0.0069 |  | 0.0326 | 0.0050 | 5.08E-01 |
| rs11934731 | 4 | 88,831,249 | *HSP90AB3P* | G | A |  | 0.0319 | 0.0074 |  | 0.0312 | 0.0054 | 9.34E-01 |
| rs2865339 | 4 | 95,099,605 | *SMARCAD1* | A | G |  | -0.0211 | 0.0070 |  | -0.0227 | 0.0051 | 8.47E-01 |
| rs62315844 | 4 | 98,446,007 | *C4orf37* | T | C |  | -0.1918 | 0.0602 |  | -0.2027 | 0.0426 | 8.83E-01 |
| rs6839437 | 4 | 146,174,631 | *OTUD4* | T | C |  | -0.0502 | 0.0093 |  | -0.0357 | 0.0067 | 2.09E-01 |
| rs35428756 | 4 | 157,511,450 | *PDGFC* | GT | G |  | 0.0191 | 0.0071 |  | 0.0254 | 0.0051 | 4.75E-01 |
| rs78014439 | 4 | 187,640,036 | *FAT1* | C | G |  | -0.0297 | 0.0135 |  | -0.0488 | 0.0099 | 2.55E-01 |
| rs6870556 | 5 | 31,134,837 | *CDH6* | G | A |  | 0.0252 | 0.0072 |  | 0.0222 | 0.0051 | 7.35E-01 |
| rs1428968 | 5 | 36,646,946 | *SLC1A3* | C | T |  | -0.0279 | 0.0091 |  | -0.0417 | 0.0065 | 2.17E-01 |
| rs2542710 | 5 | 39,382,261 | *DAB2* | A | G |  | 0.0365 | 0.0070 |  | 0.0229 | 0.0050 | 1.13E-01 |
| rs28744551 | 5 | 54,844,572 | *PPAP2A* | C | G |  | -0.0296 | 0.0119 |  | -0.0426 | 0.0086 | 3.75E-01 |
| rs9986117 | 5 | 80,270,678 | *RASGRF2* | C | G |  | -0.0228 | 0.0075 |  | -0.0279 | 0.0054 | 5.81E-01 |
| rs1010109 | 5 | 142,554,162 | *ARHGAP26* | A | G |  | 0.0269 | 0.0097 |  | 0.0327 | 0.0070 | 6.26E-01 |
| rs368510 | 5 | 148,787,469 | *LOC728264* | G | A |  | -0.0354 | 0.0073 |  | -0.0331 | 0.0053 | 7.98E-01 |
| rs393952 | 5 | 173,018,851 | *LOC285593* | G | A |  | 0.0201 | 0.0069 |  | 0.0225 | 0.0050 | 7.80E-01 |
| rs4959677 | 6 | 2,500,820 | *C6orf195* | G | C |  | 0.0256 | 0.0069 |  | 0.0265 | 0.0050 | 9.12E-01 |
| rs525678 | 6 | 7,058,857 | *RREB1* | A | G |  | 0.0794 | 0.0187 |  | 0.0744 | 0.0133 | 8.24E-01 |
| rs9379084 | 6 | 7,231,843 | *RREB1* | G | A |  | 0.0410 | 0.0110 |  | 0.0516 | 0.0080 | 4.35E-01 |
| rs74971894 | 6 | 21,888,517 | *FLJ22536* | A | G |  | 0.0523 | 0.0109 |  | 0.0391 | 0.0078 | 3.23E-01 |
| rs9260620 | 6 | 29,923,091 | *HLA-A* | T | G |  | -0.0218 | 0.0073 |  | -0.0260 | 0.0053 | 6.46E-01 |
| rs113166754 | 6 | 44,677,173 | *SUPT3H* | C | T |  | 0.1042 | 0.0143 |  | 0.0996 | 0.0103 | 7.95E-01 |
| rs72868817 | 6 | 55,608,235 | *BMP5* | A | G |  | -0.0462 | 0.0137 |  | -0.0673 | 0.0099 | 2.12E-01 |
| rs150445982 | 6 | 55,676,621 | *BMP5* | C | T |  | -0.1175 | 0.0245 |  | -0.0947 | 0.0172 | 4.46E-01 |
| rs10943125 | 6 | 74,477,152 | *CD109* | C | T |  | 0.0133 | 0.0069 |  | 0.0290 | 0.0050 | 6.64E-02 |
| rs4526167 | 6 | 83,739,225 | *UBE2CBP* | A | C |  | 0.0311 | 0.0076 |  | 0.0267 | 0.0054 | 6.33E-01 |
| rs7763784 | 6 | 127,489,818 | *RSPO3* | C | T |  | -0.0797 | 0.0070 |  | -0.0844 | 0.0050 | 5.83E-01 |
| rs7760760 | 6 | 130,379,852 | *L3MBTL3* | A | G |  | -0.0244 | 0.0075 |  | -0.0254 | 0.0054 | 9.15E-01 |
| rs9483206 | 6 | 131,367,399 | *EPB41L2* | T | C |  | 0.0253 | 0.0071 |  | 0.0321 | 0.0051 | 4.40E-01 |
| rs3777787 | 6 | 133,577,921 | *EYA4* | C | A |  | 0.0643 | 0.0069 |  | 0.0571 | 0.0050 | 4.05E-01 |
| rs547545 | 6 | 133,812,022 | *EYA4* | T | A |  | -0.0367 | 0.0069 |  | -0.0342 | 0.0050 | 7.74E-01 |
| rs12209685 | 6 | 136,228,675 | *PDE7B* | C | T |  | -0.0183 | 0.0072 |  | -0.0305 | 0.0052 | 1.70E-01 |
| rs1891002 | 6 | 151,900,047 | *C6orf97* | T | A |  | 0.0962 | 0.0077 |  | 0.1025 | 0.0055 | 5.07E-01 |
| rs2941740 | 6 | 152,009,638 | *ESR1* | A | G |  | -0.0761 | 0.0070 |  | -0.0776 | 0.0050 | 8.66E-01 |
| rs1890010 | 6 | 152,085,275 | *ESR1* | C | T |  | -0.0541 | 0.0076 |  | -0.0502 | 0.0055 | 6.73E-01 |
| rs6903009 | 6 | 155,352,813 | *TIAM2* | A | G |  | -0.0235 | 0.0073 |  | -0.0215 | 0.0052 | 8.26E-01 |
| rs73029263 | 6 | 164,113,762 | *QKI* | A | G |  | 0.0269 | 0.0102 |  | 0.0320 | 0.0074 | 6.85E-01 |
| rs2189446 | 7 | 15,632,606 | *MEOX2* | T | C |  | -0.0231 | 0.0075 |  | -0.0388 | 0.0054 | 8.71E-02 |
| rs3095208 | 7 | 20,331,713 | *ITGB8* | T | C |  | -0.0320 | 0.0078 |  | -0.0239 | 0.0057 | 3.99E-01 |
| rs85 | 7 | 25,702,026 | *MIR148A* | T | C |  | -0.0441 | 0.0086 |  | -0.0410 | 0.0062 | 7.67E-01 |
| rs62454420 | 7 | 27,191,804 | *LOC100133311* | A | G |  | -0.0205 | 0.0137 |  | -0.0659 | 0.0098 | 7.22E-03 |
| rs17501090 | 7 | 27,221,454 | *HOXA11* | C | A |  | 0.0670 | 0.0233 |  | 0.0870 | 0.0169 | 4.87E-01 |
| rs10244184 | 7 | 27,932,076 | *JAZF1* | T | C |  | 0.0436 | 0.0079 |  | 0.0397 | 0.0057 | 6.90E-01 |
| rs757980 | 7 | 28,725,536 | *CREB5* | G | A |  | 0.0448 | 0.0081 |  | 0.0378 | 0.0058 | 4.82E-01 |
| rs10236571 | 7 | 30,956,223 | *AQP1* | A | G |  | 0.0488 | 0.0082 |  | 0.0541 | 0.0059 | 5.96E-01 |
| rs1052974 | 7 | 37,946,540 | *SFRP4* | C | A |  | -0.0504 | 0.0089 |  | -0.0556 | 0.0064 | 6.37E-01 |
| rs1717747 | 7 | 38,097,903 | *EPDR1* | A | G |  | -0.0755 | 0.0089 |  | -0.0744 | 0.0064 | 9.16E-01 |
| rs643044 | 7 | 83,302,264 | *SEMA3E* | G | A |  | 0.0205 | 0.0075 |  | 0.0250 | 0.0054 | 6.25E-01 |
| rs42038 | 7 | 92,243,719 | *CDK6* | C | T |  | 0.0290 | 0.0076 |  | 0.0229 | 0.0054 | 5.16E-01 |
| rs7781370 | 7 | 96,133,531 | *FLJ42280* | T | C |  | -0.0521 | 0.0073 |  | -0.0426 | 0.0052 | 2.86E-01 |
| rs212417 | 7 | 105,494,963 | *ATXN7L1* | G | A |  | 0.0293 | 0.0074 |  | 0.0327 | 0.0053 | 7.03E-01 |
| rs2707518 | 7 | 120,954,908 | *WNT16* | G | T |  | -0.1709 | 0.0072 |  | -0.1711 | 0.0051 | 9.78E-01 |
| rs62621812 | 7 | 127,015,083 | *ZNF800* | G | A |  | 0.0872 | 0.0236 |  | 0.0960 | 0.0170 | 7.64E-01 |
| rs2929308 | 8 | 9,084,121 | *PPP1R3B* | T | A |  | 0.0459 | 0.0069 |  | 0.0392 | 0.0050 | 4.26E-01 |
| rs6471752 | 8 | 49,309,167 | *EFCAB1* | C | T |  | 0.0269 | 0.0096 |  | 0.0360 | 0.0070 | 4.43E-01 |
| rs7003794 | 8 | 71,789,146 | *XKR9* | C | A |  | 0.0301 | 0.0071 |  | 0.0274 | 0.0051 | 7.56E-01 |
| rs114847962 | 8 | 72,217,903 | *EYA1* | A | T |  | 0.0352 | 0.0079 |  | 0.0259 | 0.0057 | 3.38E-01 |
| rs6999925 | 8 | 80,786,648 | *MRPS28* | A | G |  | 0.0220 | 0.0072 |  | 0.0254 | 0.0052 | 7.02E-01 |
| rs446027 | 8 | 81,452,333 | *ZBTB10* | A | G |  | 0.0122 | 0.0074 |  | 0.0291 | 0.0054 | 6.57E-02 |
| rs2737252 | 8 | 116,663,898 | *TRPS1* | G | A |  | -0.0312 | 0.0077 |  | -0.0485 | 0.0055 | 6.74E-02 |
| rs2205270 | 8 | 117,158,377 | *TRPS1* | C | T |  | 0.0389 | 0.0103 |  | 0.0400 | 0.0075 | 9.32E-01 |
| rs117108011 | 8 | 119,901,442 | *TNFRSF11B* | A | G |  | -0.1127 | 0.0288 |  | -0.1007 | 0.0205 | 7.33E-01 |
| rs1487241 | 8 | 128,021,488 | *POU5F1B* | A | T |  | 0.0259 | 0.0074 |  | 0.0241 | 0.0054 | 8.45E-01 |
| rs6475068 | 9 | 16,693,216 | *BNC2* | G | C |  | -0.0454 | 0.0117 |  | -0.0399 | 0.0085 | 7.05E-01 |
| rs138945742 | 9 | 18,645,278 | *ADAMTSL1* | G | T |  | 0.0652 | 0.0183 |  | 0.0523 | 0.0133 | 5.67E-01 |
| rs10992867 | 9 | 96,461,013 | *PHF2* | G | A |  | -0.0303 | 0.0079 |  | -0.0308 | 0.0058 | 9.60E-01 |
| rs1057713 | 9 | 96,714,161 | *BARX1* | A | G |  | -0.0185 | 0.0071 |  | -0.0219 | 0.0051 | 7.04E-01 |
| rs1877456 | 9 | 98,275,097 | *PTCH1* | C | G |  | 0.0417 | 0.0083 |  | 0.0272 | 0.0061 | 1.58E-01 |
| rs10979249 | 9 | 110,907,520 | *KLF4* | G | T |  | 0.0224 | 0.0083 |  | 0.0391 | 0.0060 | 1.04E-01 |
| rs3802342 | 9 | 133,473,303 | *FUBP3* | C | G |  | 0.0282 | 0.0073 |  | 0.0311 | 0.0052 | 7.54E-01 |
| rs2519093 | 9 | 136,141,870 | *ABO* | C | T |  | 0.0266 | 0.0089 |  | 0.0305 | 0.0064 | 7.22E-01 |
| rs74119759 | 10 | 20,143,109 | *PLXDC2* | C | T |  | 0.0396 | 0.0096 |  | 0.0289 | 0.0069 | 3.69E-01 |
| rs6482632 | 10 | 27,890,672 | *RAB18* | T | C |  | 0.0317 | 0.0069 |  | 0.0204 | 0.0050 | 1.86E-01 |
| rs12354769 | 10 | 28,531,051 | *MPP7* | T | C |  | -0.0445 | 0.0101 |  | -0.0392 | 0.0072 | 6.70E-01 |
| rs7919498 | 10 | 31,047,997 | *ZNF438* | C | T |  | -0.0318 | 0.0070 |  | -0.0384 | 0.0050 | 4.39E-01 |
| rs17662822 | 10 | 54,412,481 | *MBL2* | C | A |  | 0.0587 | 0.0074 |  | 0.0311 | 0.0053 | 2.57E-03 |
| rs10824760 | 10 | 54,425,325 | *MBL2* | C | T |  | -0.1232 | 0.0113 |  | -0.1474 | 0.0081 | 8.08E-02 |
| rs1877998 | 10 | 79,411,740 | *KCNMA1* | G | A |  | 0.0262 | 0.0089 |  | 0.0384 | 0.0065 | 2.66E-01 |
| rs17173698 | 10 | 89,468,953 | *PAPSS2* | G | A |  | -0.0650 | 0.0216 |  | -0.0769 | 0.0158 | 6.57E-01 |
| rs603424 | 10 | 102,075,479 | *PKD2L1* | G | A |  | 0.0385 | 0.0092 |  | 0.0256 | 0.0067 | 2.55E-01 |
| rs11196170 | 10 | 114,722,621 | *TCF7L2* | G | A |  | 0.0275 | 0.0086 |  | 0.0245 | 0.0061 | 7.74E-01 |
| rs10885447 | 10 | 115,166,770 | *HABP2* | G | A |  | -0.0368 | 0.0085 |  | -0.0259 | 0.0062 | 2.99E-01 |
| rs80226362 | 10 | 134,413,500 | *INPP5A* | G | T |  | -0.0349 | 0.0089 |  | -0.0383 | 0.0064 | 7.56E-01 |
| rs2653559 | 11 | 8,900,394 | *ST5* | C | T |  | 0.0395 | 0.0094 |  | 0.0270 | 0.0067 | 2.79E-01 |
| rs61880664 | 11 | 15,343,532 | *INSC* | A | G |  | -0.0412 | 0.0116 |  | -0.0439 | 0.0083 | 8.50E-01 |
| rs78152188 | 11 | 16,635,424 | *C11orf58* | C | G |  | -0.0319 | 0.0076 |  | -0.0324 | 0.0055 | 9.59E-01 |
| rs11029901 | 11 | 27,287,108 | *CCDC34* | A | G |  | 0.0419 | 0.0073 |  | 0.0451 | 0.0052 | 7.19E-01 |
| rs117941208 | 11 | 27,346,727 | *CCDC34* | C | T |  | 0.0222 | 0.0111 |  | 0.0548 | 0.0080 | 1.75E-02 |
| rs2098878 | 11 | 35,096,310 | *CD44* | A | G |  | -0.0233 | 0.0069 |  | -0.0357 | 0.0050 | 1.45E-01 |
| rs4579897 | 11 | 47,097,364 | *C11orf49* | G | T |  | 0.0422 | 0.0074 |  | 0.0491 | 0.0053 | 4.45E-01 |
| rs1530914 | 11 | 60,028,940 | *MS4A4A* | C | T |  | -0.0341 | 0.0070 |  | -0.0280 | 0.0051 | 4.87E-01 |
| rs174574 | 11 | 61,600,342 | *FADS2* | A | C |  | 0.0153 | 0.0072 |  | 0.0305 | 0.0052 | 8.90E-02 |
| rs117111740 | 11 | 62,201,239 | *AHNAK* | T | C |  | 0.0963 | 0.0218 |  | 0.1525 | 0.0155 | 3.56E-02 |
| rs4930295 | 11 | 65,390,554 | *PCNXL3* | C | G |  | 0.0331 | 0.0083 |  | 0.0291 | 0.0060 | 6.99E-01 |
| rs61887821 | 11 | 68,099,622 | *LRP5* | G | A |  | 0.1514 | 0.0452 |  | 0.2149 | 0.0339 | 2.62E-01 |
| rs4988321 | 11 | 68,174,189 | *LRP5* | G | A |  | 0.0835 | 0.0155 |  | 0.0753 | 0.0114 | 6.70E-01 |
| rs1060435 | 11 | 68,855,595 | *TPCN2* | A | G |  | -0.0314 | 0.0070 |  | -0.0301 | 0.0051 | 8.86E-01 |
| rs149504726 | 11 | 86,653,988 | *FZD4* | G | A |  | -0.1777 | 0.0478 |  | -0.1949 | 0.0330 | 7.67E-01 |
| rs649693 | 11 | 86,869,577 | *TMEM135* | T | C |  | 0.0820 | 0.0075 |  | 0.0659 | 0.0054 | 8.27E-02 |
| rs7124639 | 11 | 112,450,073 | *C11orf34* | A | G |  | 0.0570 | 0.0070 |  | 0.0534 | 0.0051 | 6.82E-01 |
| rs10891479 | 11 | 112,826,303 | *NCAM1* | G | A |  | 0.0327 | 0.0093 |  | 0.0373 | 0.0067 | 6.92E-01 |
| rs7125361 | 11 | 115,080,042 | *CADM1* | C | G |  | -0.0424 | 0.0070 |  | -0.0423 | 0.0051 | 9.85E-01 |
| rs2846901 | 11 | 115,483,879 | *CADM1* | A | G |  | 0.0380 | 0.0097 |  | 0.0313 | 0.0070 | 5.82E-01 |
| rs10790255 | 11 | 118,515,579 | *PHLDB1* | G | T |  | -0.0361 | 0.0080 |  | -0.0280 | 0.0058 | 4.17E-01 |
| rs1622638 | 11 | 121,800,971 | *MIR100HG* | G | A |  | -0.0226 | 0.0071 |  | -0.0203 | 0.0051 | 7.90E-01 |
| rs4980826 | 12 | 578,349 | *B4GALNT3* | C | A |  | -0.0293 | 0.0070 |  | -0.0240 | 0.0051 | 5.43E-01 |
| rs9668575 | 12 | 1,636,381 | *LOC100292680* | A | G |  | -0.0458 | 0.0081 |  | -0.0315 | 0.0058 | 1.52E-01 |
| rs7959604 | 12 | 1,637,129 | *LOC100292680* | C | G |  | 0.0678 | 0.0133 |  | 0.0525 | 0.0095 | 3.48E-01 |
| rs56151937 | 12 | 2,502,996 | *CACNA1C* | T | G |  | -0.0191 | 0.0096 |  | -0.0423 | 0.0069 | 4.94E-02 |
| rs117481343 | 12 | 13,328,208 | *EMP1* | C | T |  | -0.1471 | 0.0213 |  | -0.1257 | 0.0150 | 4.13E-01 |
| rs118115924 | 12 | 49,379,537 | *WNT1* | G | T |  | 0.1747 | 0.0318 |  | 0.1866 | 0.0231 | 7.63E-01 |
| rs10875906 | 12 | 49,385,679 | *DDN* | C | T |  | -0.0308 | 0.0078 |  | -0.0285 | 0.0056 | 8.14E-01 |
| rs11832031 | 12 | 65,952,149 | *MSRB3* | T | A |  | 0.0215 | 0.0083 |  | 0.0292 | 0.0060 | 4.50E-01 |
| rs10858944 | 12 | 90,419,192 | *LOC338758* | G | A |  | -0.0682 | 0.0071 |  | -0.0549 | 0.0051 | 1.26E-01 |
| rs10859561 | 12 | 94,107,712 | *CRADD* | C | T |  | -0.0194 | 0.0069 |  | -0.0249 | 0.0050 | 5.24E-01 |
| rs112073168 | 12 | 108,985,976 | *TMEM119* | G | A |  | 0.0709 | 0.0216 |  | 0.0565 | 0.0154 | 5.89E-01 |
| rs8002850 | 13 | 22,811,940 | *FGF9* | G | A |  | 0.0239 | 0.0073 |  | 0.0273 | 0.0053 | 6.99E-01 |
| rs1328042 | 13 | 37,466,947 | *SMAD9* | G | A |  | -0.0244 | 0.0081 |  | -0.0252 | 0.0058 | 9.35E-01 |
| rs8001611 | 13 | 42,965,694 | *AKAP11* | C | T |  | -0.0482 | 0.0070 |  | -0.0471 | 0.0050 | 8.92E-01 |
| rs770379 | 13 | 51,139,771 | *DLEU7* | A | G |  | 0.0338 | 0.0070 |  | 0.0232 | 0.0050 | 2.19E-01 |
| rs3812849 | 13 | 74,701,736 | *KLF12* | A | C |  | 0.0245 | 0.0079 |  | 0.0283 | 0.0057 | 6.95E-01 |
| rs7998877 | 13 | 94,428,340 | *GPC6* | G | T |  | -0.0326 | 0.0177 |  | -0.0665 | 0.0128 | 1.20E-01 |
| rs2008411 | 13 | 99,587,929 | *DOCK9* | C | T |  | 0.0381 | 0.0076 |  | 0.0402 | 0.0054 | 8.26E-01 |
| rs1042704 | 14 | 23,312,594 | *MMP14* | G | A |  | 0.0346 | 0.0084 |  | 0.0310 | 0.0061 | 7.23E-01 |
| rs10145299 | 14 | 35,215,602 | *BAZ1A* | T | C |  | -0.0320 | 0.0070 |  | -0.0209 | 0.0051 | 1.99E-01 |
| rs4444235 | 14 | 54,410,919 | *BMP4* | T | C |  | -0.0556 | 0.0069 |  | -0.0529 | 0.0050 | 7.53E-01 |
| rs2588829 | 14 | 68,612,920 | *RAD51L1* | G | A |  | -0.0215 | 0.0093 |  | -0.0327 | 0.0068 | 3.28E-01 |
| rs56375669 | 14 | 95,629,615 | *FLJ45244* | T | C |  | 0.0309 | 0.0123 |  | 0.0489 | 0.0088 | 2.33E-01 |
| rs10139279 | 14 | 103,926,454 | *MARK3* | G | A |  | 0.0356 | 0.0074 |  | 0.0454 | 0.0053 | 2.79E-01 |
| rs11636403 | 15 | 51,548,744 | *CYP19A1* | C | T |  | -0.0352 | 0.0071 |  | -0.0355 | 0.0051 | 9.67E-01 |
| rs28587205 | 15 | 67,427,897 | *SMAD3* | A | T |  | 0.0211 | 0.0070 |  | 0.0245 | 0.0050 | 6.98E-01 |
| rs2002122 | 15 | 70,594,071 | *TLE3* | T | G |  | -0.0341 | 0.0070 |  | -0.0237 | 0.0050 | 2.26E-01 |
| rs11637971 | 15 | 91,079,863 | *CRTC3* | A | C |  | -0.0295 | 0.0075 |  | -0.0219 | 0.0054 | 4.14E-01 |
| rs2301522 | 16 | 359,953 | *AXIN1* | A | G |  | 0.0354 | 0.0073 |  | 0.0300 | 0.0053 | 5.50E-01 |
| rs34676698 | 16 | 407,549 | *AXIN1* | G | A |  | 0.1108 | 0.0236 |  | 0.1077 | 0.0164 | 9.13E-01 |
| rs6497636 | 16 | 23,053,035 | *USP31* | G | A |  | -0.0291 | 0.0076 |  | -0.0208 | 0.0054 | 3.75E-01 |
| rs1381448 | 16 | 51,723,131 | *LOC388276* | G | A |  | 0.0269 | 0.0073 |  | 0.0307 | 0.0053 | 6.77E-01 |
| rs72805220 | 16 | 51,903,981 | *LOC388276* | C | A |  | 0.0539 | 0.0142 |  | 0.0707 | 0.0103 | 3.37E-01 |
| rs8064086 | 16 | 67,013,043 | *CES3* | G | C |  | -0.0381 | 0.0102 |  | -0.0355 | 0.0073 | 8.32E-01 |
| rs17680862 | 16 | 67,322,118 | *PLEKHG4* | G | C |  | 0.0410 | 0.0214 |  | 0.1102 | 0.0155 | 8.94E-03 |
| rs4888151 | 16 | 81,559,009 | *CMIP* | A | C |  | 0.0312 | 0.0080 |  | 0.0258 | 0.0058 | 5.87E-01 |
| rs71390846 | 16 | 86,714,715 | *FOXL1* | G | C |  | 0.0510 | 0.0089 |  | 0.0368 | 0.0064 | 1.94E-01 |
| rs113478686 | 16 | 88,850,897 | *FAM38A* | C | CT |  | 0.0200 | 0.0082 |  | 0.0276 | 0.0060 | 4.56E-01 |
| rs2663339 | 17 | 927,516 | *ABR* | A | C |  | 0.0226 | 0.0075 |  | 0.0288 | 0.0054 | 5.03E-01 |
| rs11078596 | 17 | 1,618,262 | *C17orf91* | C | T |  | 0.0327 | 0.0089 |  | 0.0263 | 0.0064 | 5.60E-01 |
| rs8072532 | 17 | 2,045,273 | *SMG6* | G | A |  | -0.0539 | 0.0076 |  | -0.0561 | 0.0054 | 8.20E-01 |
| rs117573122 | 17 | 7,388,716 | *POLR2A* | G | C |  | -0.1096 | 0.0454 |  | -0.1500 | 0.0323 | 4.68E-01 |
| rs74439044 | 17 | 7,781,019 | *CHD3* | T | C |  | 0.0343 | 0.0117 |  | 0.0486 | 0.0085 | 3.25E-01 |
| rs77152265 | 17 | 9,138,318 | *NTN1* | T | C |  | 0.0211 | 0.0095 |  | 0.0422 | 0.0069 | 7.30E-02 |
| rs3760456 | 17 | 27,948,844 | *CORO6* | C | T |  | 0.0258 | 0.0070 |  | 0.0374 | 0.0050 | 1.78E-01 |
| rs7215205 | 17 | 29,818,258 | *RAB11FIP4* | T | C |  | 0.0184 | 0.0072 |  | 0.0279 | 0.0052 | 2.83E-01 |
| rs143043662 | 17 | 39,913,771 | *JUP* | C | T |  | -0.0880 | 0.0296 |  | -0.0931 | 0.0216 | 8.90E-01 |
| rs7209826 | 17 | 41,796,406 | *SOST* | A | G |  | -0.0544 | 0.0072 |  | -0.0382 | 0.0052 | 6.98E-02 |
| rs2696264 | 17 | 48,333,349 | *TMEM92* | G | A |  | -0.0422 | 0.0082 |  | -0.0267 | 0.0059 | 1.25E-01 |
| rs72829754 | 17 | 54,233,702 | *ANKFN1* | G | A |  | -0.0446 | 0.0071 |  | -0.0301 | 0.0051 | 9.77E-02 |
| rs1036902 | 17 | 58,950,791 | *BCAS3* | T | C |  | -0.0444 | 0.0098 |  | -0.0568 | 0.0071 | 3.01E-01 |
| rs11869530 | 17 | 63,549,979 | *AXIN2* | G | A |  | -0.0217 | 0.0072 |  | -0.0323 | 0.0052 | 2.34E-01 |
| rs73997493 | 17 | 75,319,800 | *SEPT9* | C | T |  | 0.0587 | 0.0124 |  | 0.0395 | 0.0089 | 2.06E-01 |
| rs12150031 | 17 | 79,420,224 | *BAHCC1* | C | G |  | 0.0249 | 0.0071 |  | 0.0231 | 0.0051 | 8.41E-01 |
| rs11875132 | 18 | 10,348,334 | *APCDD1* | T | C |  | 0.0173 | 0.0071 |  | 0.0282 | 0.0051 | 2.14E-01 |
| rs4430817 | 18 | 13,682,666 | *C18orf19* | G | C |  | 0.0503 | 0.0072 |  | 0.0489 | 0.0052 | 8.79E-01 |
| rs12967019 | 18 | 46,474,178 | *SMAD7* | T | C |  | -0.0219 | 0.0071 |  | -0.0215 | 0.0051 | 9.66E-01 |
| rs2957137 | 18 | 60,066,888 | *TNFRSF11A* | A | T |  | -0.0295 | 0.0073 |  | -0.0195 | 0.0052 | 2.66E-01 |
| rs657693 | 18 | 77,162,462 | *NFATC1* | A | G |  | -0.0281 | 0.0072 |  | -0.0180 | 0.0052 | 2.58E-01 |
| rs8108787 | 19 | 817,980 | *LPPR3* | T | C |  | 0.0209 | 0.0080 |  | 0.0385 | 0.0058 | 7.45E-02 |
| rs11084888 | 19 | 1,162,376 | *SBNO2* | G | A |  | 0.0414 | 0.0075 |  | 0.0357 | 0.0054 | 5.43E-01 |
| rs12609327 | 19 | 2,183,000 | *DOT1L* | A | C |  | 0.0186 | 0.0070 |  | 0.0255 | 0.0050 | 4.22E-01 |
| rs60507951 | 19 | 33,544,187 | *RHPN2* | G | A |  | -0.1157 | 0.0120 |  | -0.1092 | 0.0086 | 6.63E-01 |
| rs28364580 | 19 | 41,724,885 | *AXL* | G | A |  | 0.0265 | 0.0081 |  | 0.0235 | 0.0059 | 7.66E-01 |
| rs3170167 | 19 | 45,976,718 | *FOSB* | T | C |  | 0.0462 | 0.0098 |  | 0.0304 | 0.0070 | 1.89E-01 |
| rs71338564 | 20 | 6,405,944 | *FERMT1* | G | A |  | -0.0313 | 0.0073 |  | -0.0368 | 0.0053 | 5.42E-01 |
| rs6117854 | 20 | 7,551,554 | *HAO1* | G | A |  | 0.0314 | 0.0074 |  | 0.0379 | 0.0054 | 4.76E-01 |
| rs35308216 | 20 | 10,629,525 | *JAG1* | T | C |  | 0.0737 | 0.0127 |  | 0.0584 | 0.0092 | 3.29E-01 |
| rs6040286 | 20 | 10,985,143 | *LOC339593* | T | C |  | -0.0516 | 0.0070 |  | -0.0354 | 0.0050 | 6.02E-02 |
| rs34778574 | 20 | 11,150,594 | *LOC339593* | T | C |  | 0.0865 | 0.0181 |  | 0.0689 | 0.0131 | 4.31E-01 |
| rs13044413 | 20 | 33,020,957 | *ITCH* | A | G |  | -0.0292 | 0.0070 |  | -0.0205 | 0.0050 | 3.16E-01 |
| rs6016547 | 20 | 39,962,991 | *LPIN3* | A | C |  | 0.0213 | 0.0094 |  | 0.0333 | 0.0068 | 2.99E-01 |
| rs1150442 | 20 | 45,603,836 | *EYA2* | T | C |  | -0.0392 | 0.0080 |  | -0.0293 | 0.0058 | 3.17E-01 |
| rs2830907 | 21 | 28,770,608 | *NCRNA00113* | G | A |  | -0.0344 | 0.0070 |  | -0.0286 | 0.0050 | 5.03E-01 |
| rs465004 | 21 | 40,021,851 | *ERG* | T | C |  | -0.0381 | 0.0082 |  | -0.0329 | 0.0059 | 6.10E-01 |
| rs9982895 | 21 | 40,343,087 | *ETS2* | T | C |  | 0.0509 | 0.0078 |  | 0.0426 | 0.0056 | 3.87E-01 |
| rs9606139 | 22 | 19,679,303 | *SEPT5* | G | A |  | 0.1058 | 0.0114 |  | 0.1233 | 0.0082 | 2.11E-01 |
| rs2049939 | 22 | 29,483,920 | *KREMEN1* | G | C |  | 0.0391 | 0.0073 |  | 0.0427 | 0.0052 | 6.92E-01 |

**Supplementary Table 6.** Association results of weighted GRS comprising 235 SNPs associated with eBMD in UKBB with multiple skeletal sites using DXA in 1285 participants from the UK Biobank study. The weighted GRS was devised by multiplying the number of Ebmd-increasing alleles by the corresponding beta coefficient (derived from 20-fold cross validation) and summing across the 235 SNPs. ^a^ Estimates (β) are standardized differences in each BMD trait per 1-unit increase in the weighted eBMD GRS (corresponding to a 1-SD higher eBMD = 0.14 g/cm^2^).

| **Skeletal site** | **^a^ β (se) per 1-unit increase of weighted eBMD GRS** | ***P*** |
| --- | --- | --- |
|  |  |  |
| **Total BMD** | 0.477 (0.079) | 1.85×10^-9^ |
| **Head BMD** | 0.335 (0.064) | 1.98×10^-7^ |
| **Arms BMD** | 0.452 (0.054) | 7.75×10^-17^ |
| **Ribs BMD** | 0.384(0.057) | 2.87×10^-11^ |
| **Spine BMD** | 0.397(0.067) | 3.29×10^-9^ |
| **L1-L4 BMD** | 0.492 (0.066) | 1.48×10^-13^ |
| **Femur neck BMD** | 0.386 (0.064) | 2.16×10^-9^ |
| **Legs BMD** | 0.393 (0.062) | 2.25×10^-10^ |

**Supplementary Table 7.** Mendelian randomization sensitivity analysis using weighted mode MR and MR-PRESSO outlier removal.

| **Methods** | **Odds Ratio of CHD (95% CI) per 1-SD (0.14 g/cm^2^) higher eBMD** | ***P*** |
| --- | --- | --- |
|  |  |  |
| **Type 2 Diabetes** |  |  |
| Weighted mode MR | 1.09 (0.98, 1.22) | 0.110 |
| MR-PRESSO outlier removal | 1.09 (1.03, 1.15) | 0.001 |
| **Coronary heart disease** |  |  |
| Weighted mode MR | 1.07 (0.97, 1.18) | 0.149 |
| MR-PRESSO outlier removal | 1.05 (1.01, 1.09) | 0.018 |

**Supplementary Table 8.** Sensitivity analysis of Mendelian randomization estimates of eBMD and risks of T2D and CHD using sequentially more stringent *P*-value thresholds for the SNP to eBMD associations. Effect estimates correspond to a 1-SD higher eBMD (equivalent to 0.14 g/cm^2^) derived from inverse variance weighted MR.

| **GWAS threshold** | **Number of SNPs** | **PVE ^a^** | **T2D** | |  | **CHD** | |
| --- | --- | --- | --- | --- | --- | --- | --- |
|  |  |  | **OR(95% CI) ^b^** | ***P*** |  | **OR(95% CI) ^b^** | ***P*** |
| <5.0x10^-8^ | 232 | 15.9% | 1.08 (1.02, 1.14) | 0.012 |  | 1.05 (1.00, 1.10) | 0.034 |
| <5.0x10^-9^ | 191 | 14.7% | 1.08 (1.02, 1.15) | 0.009 |  | 1.05 (1.01, 1.10) | 0.027 |
| <5.0x10^-10^ | 158 | 13.7% | 1.09 (1.03, 1.15) | 0.004 |  | 1.06 (1.01, 1.11) | 0.020 |
| <5.0x10^-11^ | 135 | 12.9% | 1.09 (1.03, 1.15) | 0.003 |  | 1.05 (1.00, 1.10) | 0.039 |
| <5.0x10^-12^ | 115 | 12.2% | 1.10 (1.04, 1.17) | 0.002 |  | 1.06 (1.00, 1.11) | 0.036 |
| <5.0x10^-13^ | 101 | 11.6% | 1.10 (1.04, 1.17) | 0.003 |  | 1.05 (0.99, 1.11) | 0.089 |
| <5.0x10^-14^ | 89 | 11.1% | 1.10 (1.03, 1.17) | 0.004 |  | 1.05 (0.99, 1.11) | 0.122 |
| <5.0x10^-15^ | 81 | 10.7% | 1.09 (1.02, 1.16) | 0.009 |  | 1.06 (1.00, 1.12) | 0.050 |
| <5.0x10^-16^ | 72 | 10.2% | 1.08 (1.01, 1.15) | 0.024 |  | 1.07 (1.01, 1.13) | 0.028 |
| <5.0x10^-20^ | 53 | 9.1% | 1.09 (1.02, 1.17) | 0.020 |  | 1.08 (1.02, 1.15) | 0.013 |

^a^ PVE, proportion of variance explained were calculated by summing up the individual PVE of eBMD for each SNP using the formula $\frac{2\beta^{2}EAF(1-EAF)}{2\beta^{2}EAF\left( 1-EAF \right)+{(se(\hat{\beta}))}^{2}2N EAF(1-EAF )}$; ^b^ OR, Odds Ratio (95% Confidence interval) per 1-SD increase in eBMD.

**Supplementary Table 9.** Mendelian randomization analysis using genetic variants that are associated with DEXA-BMD at the lumbar spine (LSBMD) and femoral neck (FNBMD) as instrumental variables.

| **Conventional MR (IVW)** | **PVE (%)** | **Odds Ratio of CHD (95% CI) per 1-SD higher DEXA-BMD** | ***P*** |
| --- | --- | --- | --- |
| **Type 2 Diabetes** |  |  |  |
| FNBMD (29 SNPs) | 2.74% | 1.067 (0.938, 1.214) | 0.333 |
| LSBMD (30 SNPs) | 3.19% | 1.050 (0.936, 1.177) | 0.411 |
| **Coronary heart disease** |  |  |  |
| FNBMD (30 SNPs) | 2.80% | 1.096 (0.991, 1.213) | 0.077 |
| LSBMD (28 SNPs) | 3.06% | 1.016 (0.945, 1.093) | 0.749 |

**Supplementary Table 10.** Genetic variants that associated with BMD at the lumbar spine (LSBMD) and femoral neck (FNBMD).

| **SNP** | **Chr** | **Position** | **Locus** | **Effect Allele** | **Other Allele** | **EAF** | **β** | ***P*** | **Lead Trait** | **Reference** |
| --- | --- | --- | --- | --- | --- | --- | --- | --- | --- | --- |
| rs7521902 | 1 | 22490724 | 1p36.12 | A | C | 0.31 | -0.05 | 9.66E-11 | LSBMD | 22504420 |
| rs6426749 | 1 | 22711473 | 1p36.12 | C | G | 0.17 | 0.11 | 7.39E-57 | FNBMD | 22504420 |
| rs12407028 | 1 | 68647716 | 1p31.3 | T | C | 0.6 | 0.08 | 3.11E-45 | LSBMD | 22504420 |
| rs17482952 | 1 | 69105068 | 1p31.3 | A | G | 0.93 | 0.08 | 1.31E-11 | FNBMD | 22504420 |
| rs479336 | 1 | 1.72E+08 | 1q24.3 | T | G | 0.74 | -0.04 | 8.51E-15 | FNBMD | 22504420 |
| rs7584262 | 2 | 42250549 | 2p21 | T | C | 0.23 | 0.04 | 1.27E-09 | FNBMD | 22504420 |
| rs4233949 | 2 | 54659707 | 2p16.2 | C | G | 0.38 | 0.05 | 2.25E-18 | LSBMD | 22504420 |
| rs17040773 | 2 | 1.13E+08 | 2q13 | A | C | 0.76 | 0.04 | 1.51E-09 | FNBMD | 22504420 |
| rs1878526 | 2 | 1.19E+08 | 2q14.2 | A | G | 0.22 | 0.04 | 1.22E-10 | LSBMD | 22504420 |
| rs1346004 | 2 | 1.67E+08 | 2q24.3 | A | G | 0.5 | -0.06 | 3.87E-30 | LSBMD | 22504420 |
| rs430727 | 3 | 41128564 | 3p22.1 | T | C | 0.48 | -0.06 | 4.41E-25 | FNBMD | 22504420 |
| rs1026364 | 3 | 1.13E+08 | 3q13.2 | T | G | 0.37 | 0.03 | 4.08E-10 | FNBMD | 22504420 |
| rs344081 | 3 | 1.57E+08 | 3q25.31 | T | C | 0.87 | 0.06 | 4.46E-12 | LSBMD | 22504420 |
| rs3755955 | 4 | 994414 | 4p16.3 | A | G | 0.16 | -0.06 | 5.24E-15 | LSBMD | 22504420 |
| rs6532023 | 4 | 88773849 | 4q22.1 | T | G | 0.34 | 0.06 | 1.23E-27 | LSBMD | 22504420 |
| rs1366594 | 5 | 88376061 | 5q14.3 | A | C | 0.54 | -0.08 | 4.47E-61 | FNBMD | 22504420 |
| rs9466056 | 6 | 21384613 | 6p22.3 | A | G | 0.38 | -0.04 | 2.73E-13 | FNBMD | 22504420 |
| rs11755164 | 6 | 44639184 | 6p21.1 | T | C | 0.4 | -0.04 | 5.6E-11 | LSBMD | 22504420 |
| rs13204965 | 6 | 1.27E+08 | 6q22.32 | A | C | 0.76 | -0.04 | 8.12E-12 | FNBMD | 22504420 |
| rs10226308 | 7 | 37938422 | 7p14.1 | A | G | 0.84 | -0.06 | 6.4E-13 | LSBMD | 22504420 |
| rs6959212 | 7 | 38128326 | 7p14.1 | T | C | 0.32 | -0.07 | 3.76E-38 | LSBMD | 22504420 |
| rs4727338 | 7 | 96120675 | 7q21.3 | C | G | 0.67 | 0.08 | 8.1E-48 | FNBMD | 22504420 |
| rs13245690 | 7 | 1.21E+08 | 7q31.31 | A | G | 0.65 | -0.05 | 1.65E-11 | LSBMD | 22504420 |
| rs3801387 | 7 | 1.21E+08 | 7q31.31 | A | G | 0.74 | -0.09 | 3.17E-51 | LSBMD | 22504420 |
| rs7812088 | 7 | 1.51E+08 | 7q36.1 | A | G | 0.13 | -0.05 | 7.28E-09 | FNBMD | 22504420 |
| rs7017914 | 8 | 71591203 | 8q13.3 | A | G | 0.49 | 0.03 | 2.29E-07 | FNBMD | 22504420 |
| rs2062377 | 8 | 1.2E+08 | 8q24.12 | A | T | 0.57 | -0.08 | 3.16E-39 | LSBMD | 22504420 |
| rs7851693 | 9 | 1.33E+08 | 9q34.11 | C | G | 0.64 | 0.05 | 3.37E-22 | FNBMD | 22504420 |
| rs3905706 | 10 | 28479942 | 10p11.23 | T | C | 0.22 | 0.05 | 2.41E-16 | LSBMD | 22504420 |
| rs1373004 | 10 | 54427825 | 10q21.1 | T | G | 0.13 | -0.06 | 1.56E-12 | LSBMD | 22504420 |
| rs7071206 | 10 | 79401316 | 10q22.3 | T | C | 0.78 | -0.06 | 5.02E-19 | LSBMD | 22504420 |
| rs7084921 | 10 | 1.02E+08 | 10q24.2 | T | C | 0.39 | 0.03 | 9.03E-10 | FNBMD | 22504420 |
| rs7108738 | 11 | 15710084 | 11p15.2 | T | G | 0.83 | -0.08 | 1.08E-32 | FNBMD | 22504420 |
| rs10835187 | 11 | 27505677 | 11p14.1 | T | C | 0.55 | -0.03 | 4.9E-08 | LSBMD | 22504420 |
| rs163879 | 11 | 30951674 | 11p14.1 | T | C | 0.68 | -0.04 | 2.19E-11 | LSBMD | 22504420 |
| rs7932354 | 11 | 46722221 | 11p11.2 | T | C | 0.31 | 0.05 | 5.12E-18 | FNBMD | 22504420 |
| rs3736228 | 11 | 68201295 | 11q13.2 | T | C | 0.16 | -0.08 | 2.08E-26 | LSBMD | 22504420 |
| rs2887571 | 12 | 1638171 | 12p13.33 | A | G | 0.76 | -0.04 | 5.59E-12 | LSBMD | 22504420 |
| rs7953528 | 12 | 28017159 | 12p11.22 | A | T | 0.18 | 0.05 | 1.87E-12 | FNBMD | 22504420 |
| rs12821008 | 12 | 49474605 | 12q13.12 | T | C | 0.39 | 0.05 | 1.17E-15 | LSBMD | 22504420 |
| rs2016266 | 12 | 53727955 | 12q13.13 | A | G | 0.68 | -0.05 | 2.95E-20 | LSBMD | 22504420 |
| rs736825 | 12 | 54417576 | 12q13.13 | C | G | 0.56 | 0.05 | 7.68E-16 | LSBMD | 22504420 |
| rs1053051 | 12 | 1.07E+08 | 12q23.3 | T | C | 0.52 | 0.03 | 9.6E-10 | FNBMD | 22504420 |
| rs9533090 | 13 | 42951449 | 13q14.11 | T | C | 0.49 | -0.1 | 4.82E-68 | LSBMD | 22504420 |
| rs1286083 | 14 | 91442779 | 14q32.12 | T | C | 0.81 | -0.05 | 2.02E-15 | FNBMD | 22504420 |
| rs11623869 | 14 | 1.04E+08 | 14q32.32 | T | G | 0.35 | -0.04 | 5.2E-16 | FNBMD | 22504420 |
| rs9921222 | 16 | 375782 | 16p13.3 | T | C | 0.48 | -0.04 | 1E-16 | LSBMD | 22504420 |
| rs13336428 | 16 | 1532463 | 16p13.3 | A | G | 0.43 | -0.04 | 1.49E-16 | FNBMD | 22504420 |
| rs4985155 | 16 | 15129459 | 16p13.11 | A | G | 0.67 | -0.03 | 1.74E-10 | FNBMD | 22504420 |
| rs1564981 | 16 | 50986308 | 16q12.1 | A | G | 0.5 | -0.04 | 1.95E-10 | LSBMD | 22504420 |
| rs1566045 | 16 | 51021803 | 16q12.1 | T | C | 0.8 | -0.06 | 1.94E-22 | FNBMD | 22504420 |
| rs10048146 | 16 | 86710660 | 16q24.1 | A | G | 0.8 | 0.05 | 1E-14 | FNBMD | 22504420 |
| rs4790881 | 17 | 2068932 | 17p13.3 | A | C | 0.69 | 0.05 | 9.75E-19 | FNBMD | 22504420 |
| rs4792909 | 17 | 41798824 | 17q21.31 | T | G | 0.37 | 0.04 | 1.95E-11 | FNBMD | 22504420 |
| rs227584 | 17 | 42225547 | 17q21.31 | A | C | 0.7 | -0.06 | 2.56E-24 | FNBMD | 22504420 |
| rs1864325 | 17 | 43977827 | 17q21.31 | T | C | 0.22 | -0.04 | 4.89E-11 | LSBMD | 22504420 |
| rs7217932 | 17 | 69949016 | 17q24.3 | A | G | 0.46 | 0.03 | 1.92E-11 | FNBMD | 22504420 |
| rs4796995 | 18 | 13708574 | 18p11.21 | A | G | 0.63 | 0.03 | 4.85E-08 | FNBMD | 22504420 |
| rs884205 | 18 | 60054857 | 18q21.33 | A | C | 0.27 | -0.05 | 1.58E-17 | LSBMD | 22504420 |
| rs3790160 | 20 | 10639988 | 20p12.2 | T | C | 0.5 | 0.05 | 3.07E-19 | LSBMD | 22504420 |

EAF, effect allele frequency

**Supplementary Table 11.** Genetic liability to type 2 diabetes or coronary heart disease and its impact on eBMD

| **Methods** | **Standardized eBMD (95% CI) per 1 log-odds higher liability to T2D or CHD** | ***P*** |
| --- | --- | --- |
|  |  |  |
| **Type 2 Diabetes (94 SNPs)** | |  |
| **Conventional MR (IVW)** | 0.017 (-0.004, 0.037) | 0.116 |
| **MR-Egger** | 0.019 (-0.019, 0.057) | 0.319 |
| Intercept | 0.00 (-0.003, 0.003) | 0.877 |
| **Coronary heart disease (52 SNPs)** | |  |
| **Conventional MR (IVW)** | 0.010(-0.024, 0.043) | 0.578 |
| **MR-Egger** | -0.033 (-0.101, 0.035) | 0.344 |
| Intercept | 0.004 (-0.002, 0.010) | 0.161 |

**Supplementary Table 12.** Characteristics of 94 SNPs associated with type 2 diabetes and 52 SNPs associated with coronary heart disease used for Mendelian randomization in the reverse direction.

| **Locus** | **SNP** | **Chr** | **Position** | **Risk Allele** | **Other Allele** | **Risk allele frequency** |
| --- | --- | --- | --- | --- | --- | --- |
| Type 2 diabetes associated SNPs | | | | | | |
| *MACF1* | rs2296172 | 1 | 39835817 | G | A | 0.19 |
| *FAF1* | rs17106184 | 1 | 50909985 | G | A | 0.90 |
| *NOTCH2* | rs10923931 | 1 | 120517959 | T | G | 0.11 |
| *ATP8B2* | rs67156297 | 1 | 154336716 | A | G | 0.25 |
| *PROX1* | rs2075423 | 1 | 214154719 | G | T | 0.63 |
| *GCKR* | rs780094 | 2 | 27741237 | C | T | 0.59 |
| *THADA* | rs10203174 | 2 | 43690030 | C | T | 0.90 |
| *ASB3* | rs9309245 | 2 | 53397048 | G | C | 0.35 |
| *CCDC85A* | rs1116357 | 2 | 57287411 | G | A | 0.53 |
| *BCL11A* | rs243088 | 2 | 60568745 | T | A | 0.45 |
| *RBMS1* | rs7593730 | 2 | 161171454 | C | T | 0.79 |
| *GRB14* | rs13389219 | 2 | 165528876 | C | T | 0.56 |
| *IRS1* | rs2943640 | 2 | 227093585 | C | A | 0.64 |
| *DNER* | rs1861612 | 2 | 230522398 | A | G | 0.55 |
| *PPARG* | rs1801282 | 3 | 12393125 | C | G | 0.88 |
| *UBE2E2* | rs1496653 | 3 | 23454790 | A | G | 0.78 |
| *PSMD6* | rs831571 | 3 | 64048297 | C | T | 0.79 |
| *ADAMTS9* | rs6795735 | 3 | 64705365 | C | T | 0.56 |
| *ADCY5* | rs11717195 | 3 | 123082398 | T | C | 0.83 |
| *IGF2BP2* | rs4402960 | 3 | 185511687 | T | G | 0.30 |
| *ST6GAL1* | rs16861329 | 3 | 186666461 | C | T | 0.87 |
| *LPP* | rs6808574 | 3 | 187740523 | C | T | 0.61 |
| *MAEA* | rs6815464 | 4 | 1309901 | C | G | 0.97 |
| *WFS1* | rs4458523 | 4 | 6289986 | G | T | 0.63 |
| *TMEM154* | rs6813195 | 4 | 153520475 | C | T | 0.71 |
| *ARL15* | rs702634 | 5 | 53271420 | A | G | 0.69 |
| *ANKRD55* | rs459193 | 5 | 55806751 | G | A | 0.71 |
| *ZBED3* | rs6878122 | 5 | 76427311 | G | A | 0.33 |
| *PAM* | rs35658696 | 5 | 102338811 | G | A | 0.04 |
| *SSR1/RREB1* | rs9505118 | 6 | 7290437 | A | G | 0.58 |
| *CDKAL1* | rs10440833 | 6 | 20688121 | A | T | 0.28 |
| *POU5F1* | rs3132524 | 6 | 31136714 | C | T | 0.78 |
| *HLA-B* | rs2244020 | 6 | 31347451 | G | A | 0.41 |
| *ZFAND3* | rs9470794 | 6 | 38106844 | C | T | 0.09 |
| *KCNK16* | rs1535500 | 6 | 39284050 | T | G | 0.49 |
| *TCF7L2* | rs11759026 | 6 | 126792095 | G | A | 0.25 |
| *DGKB* | rs17168486 | 7 | 14898282 | T | C | 0.17 |
| *JAZF1* | rs849135 | 7 | 28196413 | G | A | 0.50 |
| *GCK* | rs4607517 | 7 | 44235668 | A | G | 0.18 |
| *PAX4, ARF5, SND1* | rs10229583 | 7 | 127246903 | G | A | 0.76 |
| *MIR129, LEP* | rs791595 | 7 | 127862802 | A | G | 0.18 |
| *KLF14* | rs972283 | 7 | 130466854 | G | A | 0.56 |
| *ANK1* | rs515071 | 8 | 41519462 | G | A | 0.77 |
| *TP53INP1* | rs896854 | 8 | 95960511 | T | C | 0.46 |
| *SLC30A8* | rs3802177 | 8 | 118185025 | G | A | 0.72 |
| *GLIS3* | rs7041847 | 9 | 4287466 | A | G | 0.53 |
| *PTPRD* | rs17584499 | 9 | 8879118 | T | C | 0.18 |
| *CDKN2A/B* | rs944801 | 9 | 22051670 | C | G | 0.58 |
| *CDKN2A/B* | rs10811661 | 9 | 22134094 | T | C | 0.83 |
| *DMRTA1* | rs1575972 | 9 | 22301092 | T | A | 0.97 |
| *TLE4* | rs13292136 | 9 | 81952128 | C | T | 0.94 |
| *TLE1* | rs2796441 | 9 | 84308948 | G | A | 0.58 |
| *GPSM1* | rs11787792 | 9 | 139252148 | A | G | 0.67 |
| *CDC123/CAMK1D* | rs12779790 | 10 | 12328010 | G | A | 0.20 |
| *VPS26A* | rs1802295 | 10 | 70931474 | T | C | 0.29 |
| *ZMIZ1* | rs12571751 | 10 | 80942631 | A | G | 0.55 |
| *HHEX/IDE* | rs5015480 | 10 | 94465559 | C | T | 0.57 |
| *TCF7L2* | rs7903146 | 10 | 114758349 | T | C | 0.32 |
| *GRK5* | rs10886471 | 10 | 121149403 | C | T | 0.52 |
| *DUSP8* | rs2334499 | 11 | 1696849 | T | C | 0.43 |
| *MIR4686* | rs7107784 | 11 | 2215089 | G | A | 0.28 |
| *KCNQ1* | rs231361 | 11 | 2691500 | A | G | 0.24 |
| *KCNQ1* | rs163184 | 11 | 2847069 | G | T | 0.49 |
| *KCNJ11* | rs5215 | 11 | 17408630 | C | T | 0.35 |
| *CENTD2, ARAP1* | rs1552224 | 11 | 72433098 | A | C | 0.85 |
| *MTNR1B* | rs10830963 | 11 | 92708710 | G | C | 0.29 |
| *CCND2* | rs11063069 | 12 | 4374373 | G | A | 0.20 |
| *KLHDC5* | rs10842994 | 12 | 27965150 | C | T | 0.79 |
| *HMGA2* | rs2261181 | 12 | 66212318 | T | C | 0.12 |
| *TSPAN8/LGR5* | rs7955901 | 12 | 71433293 | C | T | 0.44 |
| *HNF1A* | rs7957197 | 12 | 121460686 | T | A | 0.80 |
| *MPHOSPH9* | rs4275659 | 12 | 123447928 | C | T | 0.69 |
| *SPRY2* | rs1359790 | 13 | 80717156 | G | A | 0.73 |
| *RASGRP1* | rs7403531 | 15 | 38822905 | T | C | 0.22 |
| *INFAFM2* | rs67839313 | 15 | 40619724 | C | T | 0.11 |
| *C2CD4A,C2CD4B* | rs7172432 | 15 | 62396389 | A | G | 0.59 |
| *HMG20A* | rs7178572 | 15 | 77747190 | G | A | 0.69 |
| *ZFAND6* | rs11634397 | 15 | 80432222 | G | A | 0.66 |
| *AP3S2* | rs2028299 | 15 | 90374257 | C | A | 0.27 |
| *PRC1* | rs12899811 | 15 | 91544076 | G | A | 0.31 |
| *FTO* | rs9936385 | 16 | 53819169 | C | T | 0.41 |
| *BCAR1* | rs7202877 | 16 | 75247245 | T | G | 0.90 |
| *SRR* | rs391300 | 17 | 2216258 | C | T | 0.61 |
| *SLC16A13* | rs312457 | 17 | 6940393 | G | A | 0.02 |
| *HNF1B (TCF2)* | rs11651755 | 17 | 36099840 | C | T | 0.48 |
| *LAMA1* | rs8090011 | 18 | 7068462 | G | C | 0.38 |
| *MC4R* | rs12970134 | 18 | 57884750 | A | G | 0.27 |
| *BCL2* | rs12454712 | 18 | 60845884 | T | C | 0.61 |
| *CILP2* | rs10401969 | 19 | 19407718 | C | T | 0.07 |
| *PEPD* | rs3786897 | 19 | 33893008 | A | G | 0.60 |
| *GIPR* | rs8108269 | 19 | 46158513 | G | T | 0.30 |
| *FITM2,R3HDML,HNF4A* | rs6017317 | 20 | 42946966 | G | T | 0.16 |
| *HNF4A* | rs4812829 | 20 | 42989267 | A | G | 0.16 |
| *MTMR3* | rs41278853 | 22 | 30416527 | A | G | 0.94 |
| Coronary heart disease associated SNPs | | | | | | |
| *PCSK9* | rs11206510 | 1 | 55496039 | T | C | 0.85 |
| *PPAP2B* | rs9970807 | 1 | 56965664 | C | T | 0.92 |
| *SORT1* | rs7528419 | 1 | 109817192 | A | G | 0.79 |
| *IL6R* | rs6689306 | 1 | 154395946 | G | A | 0.55 |
| *MIA3* | rs67180937 | 1 | 222823743 | G | T | 0.66 |
| *AK097927* | rs16986953 | 2 | 19942473 | G | A | 0.90 |
| *VAMP5-VAMP8-GGCX* | rs7568458 | 2 | 85788175 | T | A | 0.55 |
| *ZEB2-ACO74093.1* | rs17678683 | 2 | 145286559 | T | G | 0.91 |
| *MRAS* | chr3:138099161 | 3 | 138099161 | D | I | 0.84 |
| *REST - NOA1* | rs17087335 | 4 | 57838583 | G | T | 0.79 |
| *EDNRA* | rs4593108 | 4 | 148281001 | C | G | 0.80 |
| *GUCY1A3* | rs72689147 | 4 | 156639888 | G | T | 0.82 |
| *SLC22A4-SLC22A5* | rs273909 | 5 | 131667353 | A | G | 0.88 |
| *ADTRP-C6orf105* | rs932344 | 6 | 11719855 | G | A | 0.40 |
| *PHACTR1* | rs9349379 | 6 | 12903957 | A | G | 0.57 |
| *ANKS1A* | rs3822921 | 6 | 35057331 | G | A | 0.89 |
| *KCNK5* | rs56336142 | 6 | 39134099 | T | C | 0.81 |
| *LPA* | rs55730499 | 6 | 161005610 | C | T | 0.94 |
| *PLG* | rs2315065 | 6 | 161108144 | C | A | 0.94 |
| *HDAC9* | rs2107595 | 7 | 19049388 | G | A | 0.80 |
| *7q22* | rs68170813 | 7 | 107259721 | T | C | 0.78 |
| *ZC3HC1* | rs11556924 | 7 | 129663496 | C | T | 0.69 |
| *NOS3* | rs3918226 | 7 | 150690176 | C | T | 0.94 |
| *TRIB1* | rs2001846 | 8 | 126478450 | C | T | 0.52 |
| *9p21* | rs2891168 | 9 | 22098619 | A | G | 0.51 |
| *ABO* | rs2519093 | 9 | 136141870 | C | T | 0.81 |
| *KIAA1462* | rs2487928 | 10 | 30323892 | G | A | 0.58 |
| *CXCL12* | rs1870634 | 10 | 44480811 | G | T | 0.64 |
| *LIPA* | rs1412444 | 10 | 91002927 | C | T | 0.63 |
| *CYP17A1-CNNM2-NT5C2* | rs11191416 | 10 | 104604916 | T | G | 0.87 |
| *SWAP70* | rs10840293 | 11 | 9751196 | A | G | 0.55 |
| *PDGFD* | rs2128739 | 11 | 103673277 | C | A | 0.68 |
| *ZNF259-APOA5-APOA1* | rs964184 | 11 | 116648917 | C | G | 0.82 |
| *ATP2B1* | rs2681472 | 12 | 90008959 | A | G | 0.80 |
| *SH2B3* | rs11065979 | 12 | 112059557 | C | T | 0.63 |
| *FLT1* | rs1924981 | 13 | 29022645 | C | T | 0.66 |
| *COL4A1/A2* | rs11617955 | 13 | 110818102 | T | A | 0.89 |
| *COL4A1/A2* | rs11838776 | 13 | 111040681 | G | A | 0.74 |
| *HHIPL1* | rs10139550 | 14 | 100145710 | C | G | 0.58 |
| *SMAD3* | rs56062135 | 15 | 67455630 | C | T | 0.79 |
| *ADAMTS7* | rs4468572 | 15 | 79124475 | C | T | 0.59 |
| *MFGE8 - ABHD2* | rs8042271 | 15 | 89574218 | A | G | 0.10 |
| *FURIN-FES* | rs2521501 | 15 | 91437388 | A | T | 0.70 |
| *SMG6* | rs9914266 | 17 | 2133250 | T | C | 0.65 |
| *RAI1-PEMT-RASD1* | rs7214245 | 17 | 17591759 | T | A | 0.56 |
| *UBE2Z* | rs35895680 | 17 | 47060322 | C | A | 0.72 |
| *BCAS3* | rs7212798 | 17 | 59013488 | T | C | 0.85 |
| *PMAIP1 - MC4R* | rs663129 | 18 | 57838401 | G | A | 0.74 |
| *LDLR* | rs56289821 | 19 | 11188247 | G | A | 0.90 |
| *APOC1* | rs4420638 | 19 | 45422946 | A | G | 0.83 |
| *KCNE2* | rs28451064 | 21 | 35593827 | G | A | 0.88 |
| *POM121L9P* | rs180803 | 22 | 24658858 | T | G | 0.03 |

**Supplementary Figure 1.** Quantile-quantile (Q-Q) plot of the GWAS results before (black) and after (blue) removing SNPs within 1 Mb of the previously reported genome-wide significant hits for different bone mineral density measurements.
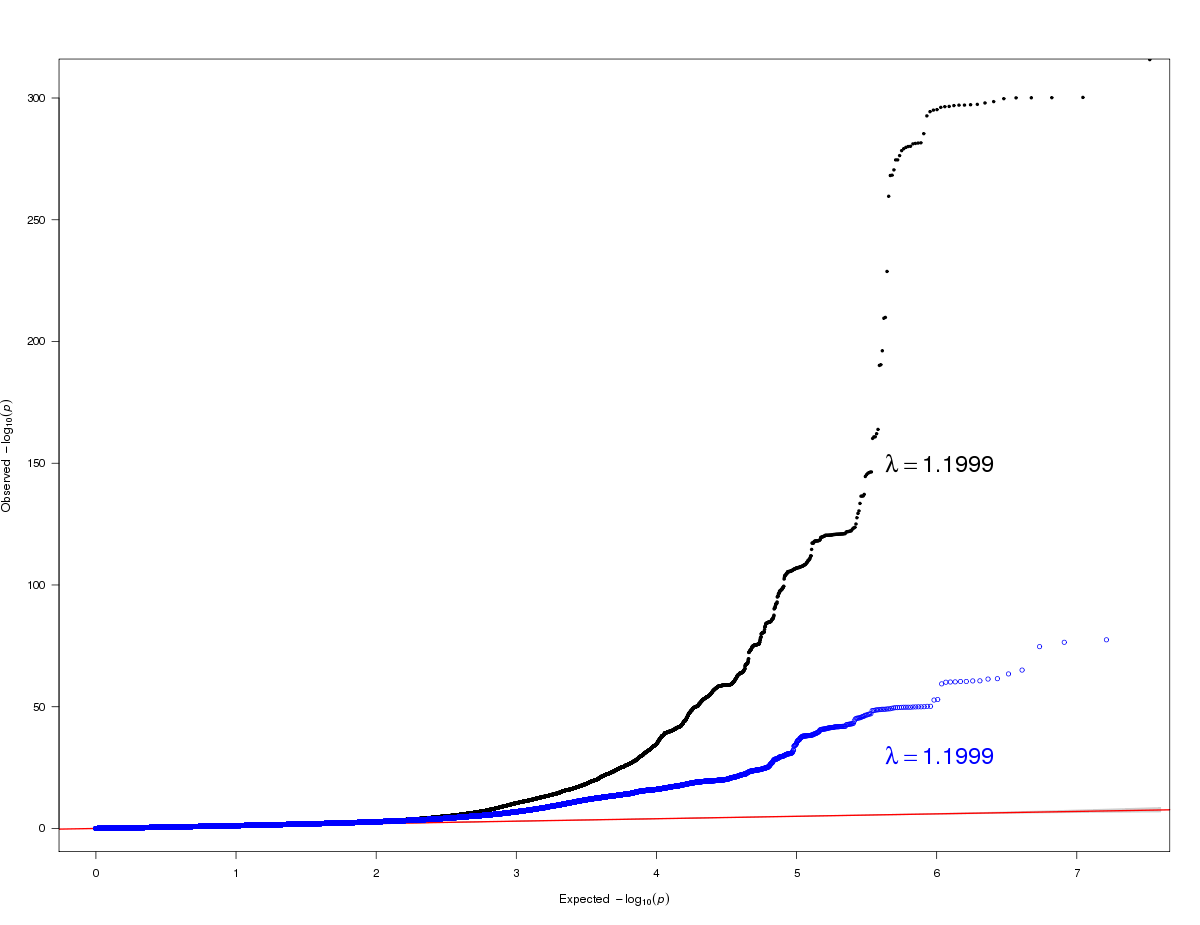


**Supplementary Figure 2.** Comparison of the effect estimates of femoral neck BMD (FNBMD, **A**) and lumbar spine BMD (LSBMD, **B**) related SNPs with their effect on heel eBMD (eBMD).


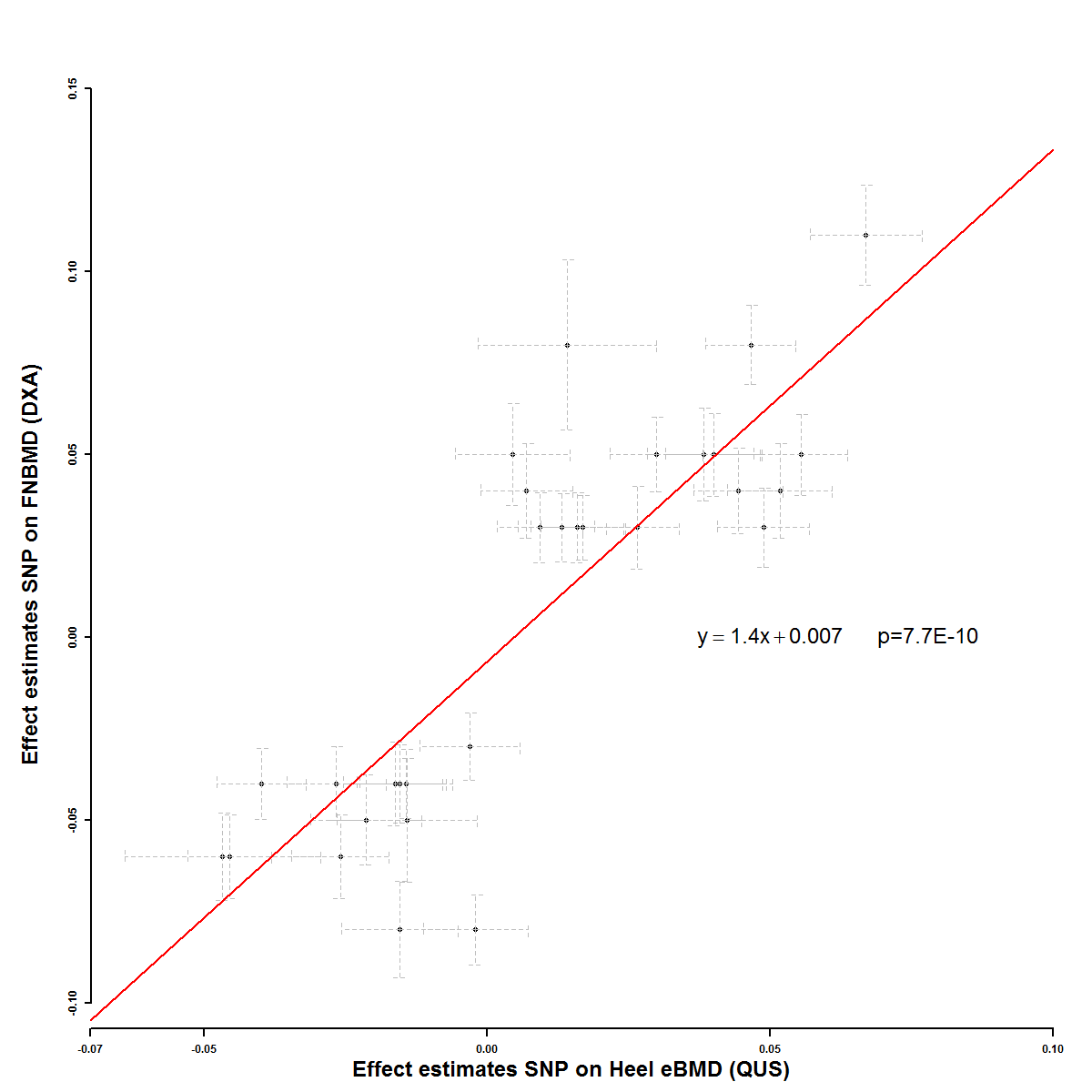

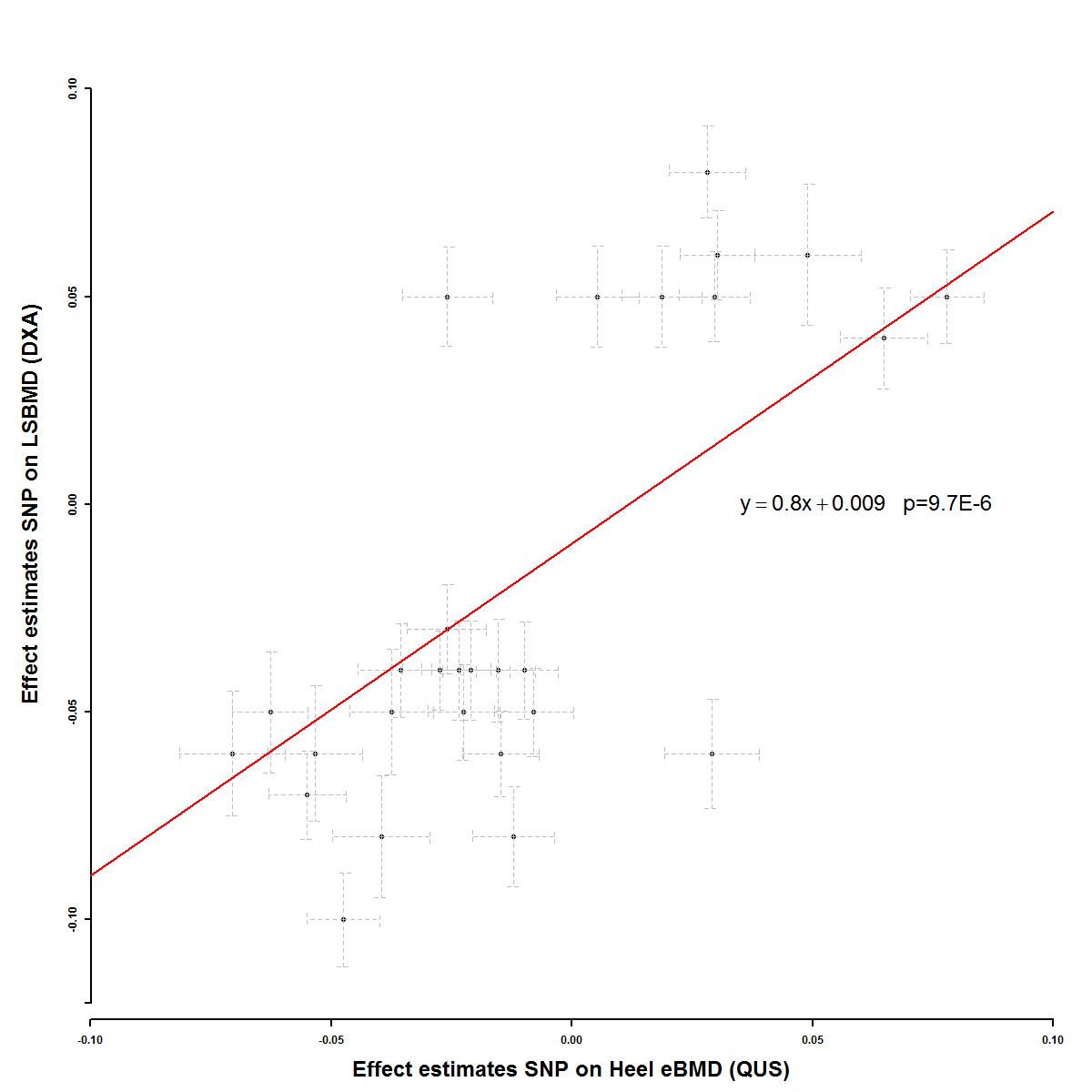


**B**

**A**

**Supplementary Figure 3.** Individual instrumental variable estimates for each of the 232 eBMD-associated SNPs with risks of type 2 diabetes (A) and coronary heart diseases (B ).


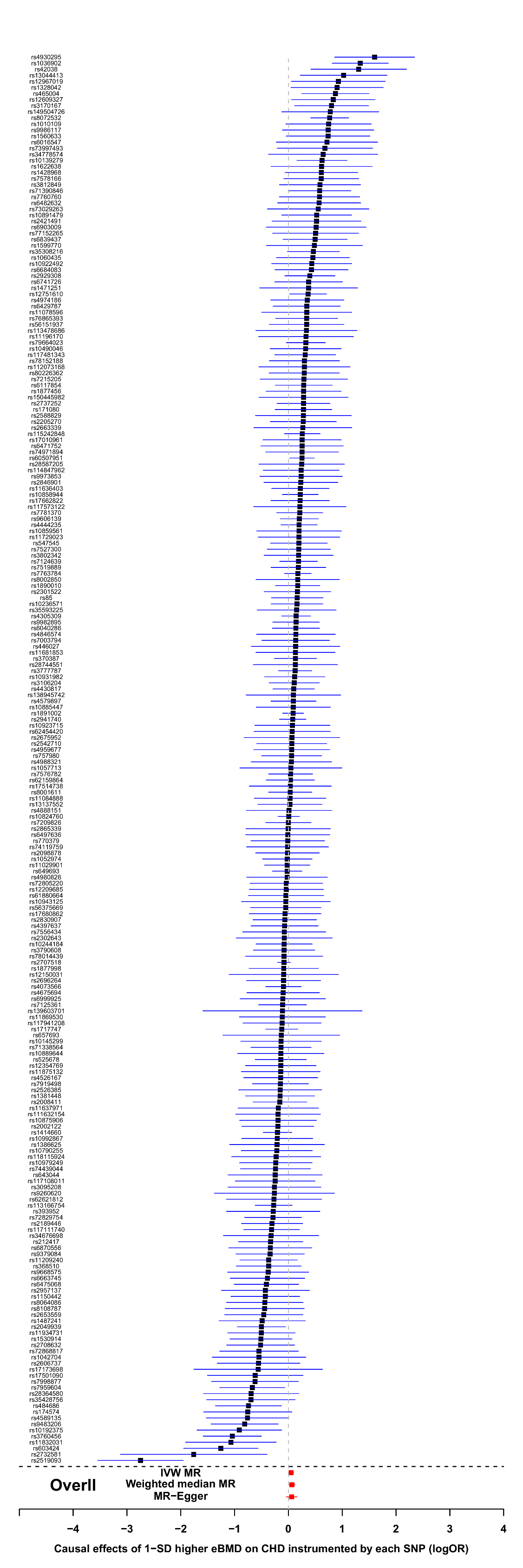

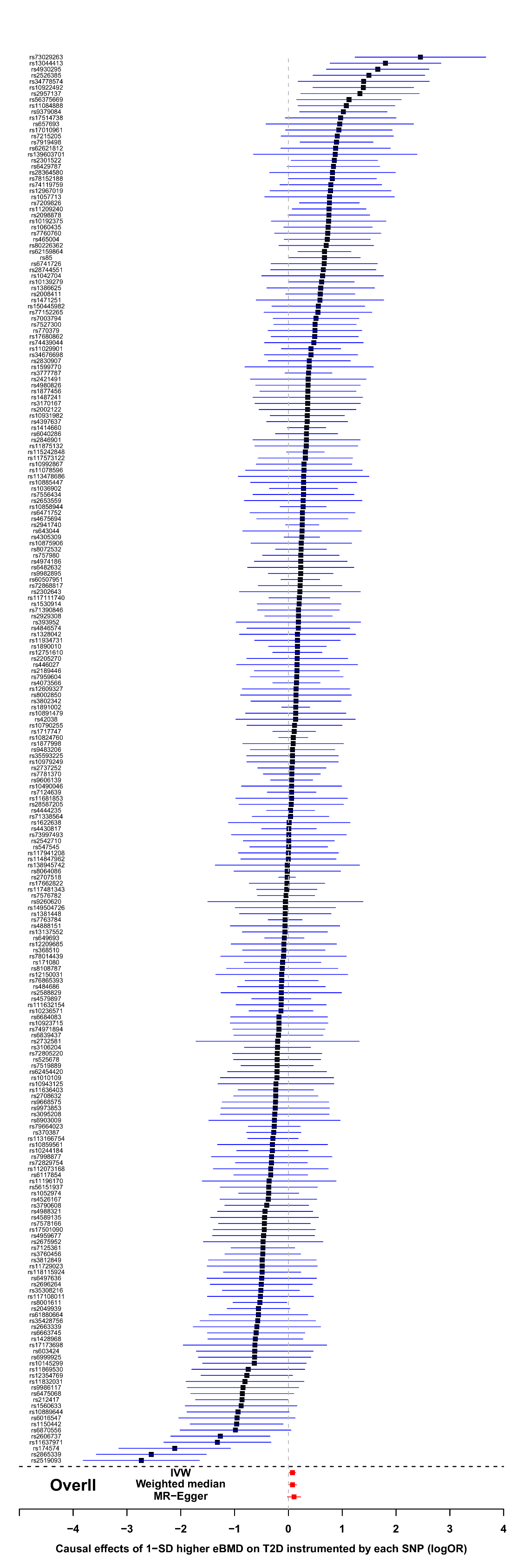


**A**

**B**

**Supplementary Figure 4.** Funnel plot of the instrument strength against causal estimates of eBMD on type 2 diabetes (A) and coronary heart disease (B).

**A**

**B**
