# Supplementary material for: Bone mineral density and risk of type 2 diabetes and coronary heart disease: A Mendelian randomization study
Source: Wellcome Open Res. 2017 Aug 22;2:68. [Version 1] doi: 10.12688/wellcomeopenres.12288.1 (PMC5606062; doi:10.12688/wellcomeopenres.12288.1)
Supplement: Peer review history

# Peer review: Referee response for version 1

**DOI:** 10.21956/wellcomeopenres.13302.r25236

**Published:** 2017-09-18

**Authors:** Guillaume Paré, Sébastien Thériault

**Affiliations:** 1 Department of Pathology and Molecular Medicine, McMaster University, Hamilton, ON, Canada; 2 Department of Molecular Biology, Medical Biochemistry and Pathology, Laval University, Quebec City, QC, Canada

The authors report an extensive genetic analysis looking at the relationship between bone mineral density (BMD), type 2 diabetes (T2D) and coronary heart disease (CHD). A genome-wide association study using the UK Biobank first release allowed the identification of 235 SNPs associated with estimated heel BMD (eBMD) using quantitative ultrasound. These SNPs were then used as instrumental variables in Mendelian randomization (MR) analyses which led the authors to conclude that an elevated eBMD is causally associated with higher risk of T2D and CHD.

 The article helps to answer an interesting question using the latest Mendelian randomization methods and recent sources of publicly available genetic data. The article is well organized and clearly written. The authors performed several analyses to ensure the robustness of the findings.

 In the conclusions, the authors suggest that BMD is directly causal for T2D and CHD, which is a simplified interpretation of the findings. It is plausible that variants affecting bone mineral density do so through biological pathways themselves responsible for the effect on T2D and CHD risk (i.e. osteocalcin leading to insulin resistance, as mentioned by the authors in the discussion). In other words, factors leading to elevated BMD could be causing T2D and CHD rather than BMD itself. Only certain pathways might be involved, especially considering that the overall effect is weak and in the opposite direction for an important proportion of the 235 variants. As noted by the authors, a parallel can be made with the decreased risk of CAD with increasing height. In that case, it seems even more plausible that factors leading to increase height are responsible for the association rather than height itself. For these reasons, we believe that the conclusions should be revised to take into account this interpretation. 

 MR associations remain weakly significant and many hypotheses have been tested, the authors should also take this into account in their conclusion.

 We suggest also testing height and waist-to-hip ratio as potential risk factors associated with the eBMD genetic instrument (i.e. in Table 2).

 This statement in the Discussion should be revised: “However, the lack of a negative association of CHD SNPs with eBMD argues against the presence of CHD causally impacting on eBMD, and thus the discrepancy between the observational analysis and MR findings warrants further investigation.” This does not take into account the possibility that it is the CHD event that leads participants to decrease activity, such that no genetic association would be observed in individuals free of CHD, even if genetically predisposed.

 We also have a few minor suggestions:

- Characteristics of the UK Biobank participants included in the analysis (first genotyping release) should be described in Table 1 (n=116,501) in addition to the whole UK Biobank European population (n=457,395).
- In the Results section, it would be more logical to present the eBMD GWAS results before the results of the association between eBMD and T2D/CHD, and invert Figure 2 and 3.
- We suggest replacing the term "contribute" by "associated" in the header of Table 2.
- A figure showing the association in terms of loci (instead of rs numbers only) would be interesting to get a better idea of the pathways involved. We suggest for example using only the stronger SNPs (p-value < 5X10 ^-20^) and plotting the effect on eBMD vs the effect on T2D and CHD.
- It should be mentioned in the limitations that applicability to non-European ethnic groups remains uncertain as mostly European studies were used in the analysis.
- In the Conclusions section, the word “casual” should read “causal”.

 

 In the Supplementary Material:

- The font of axes labels in Supplementary Figures 1, 2 and 4 should be increased to facilitate reading.
- The axes should be added to Supplementary Figure 2 to help visualize which loci have a consistent direction of effect.
- In Supplementary Figure 3, “Overll” should read “Overall”.

We have read this submission. We believe that we have an appropriate level of expertise to confirm that it is of an acceptable scientific standard.

# Peer review: Referee response for version 1

**DOI:** 10.21956/wellcomeopenres.13302.r25703

**Published:** 2017-09-15

**Authors:** Christina Ellervik

**Affiliations:** 1 Faculty of Health and Medical Sciences, University of Copenhagen, Copenhagen, Denmark

The authors have undertaken a GWAS and bidirectional Mendelian Randomization study of the relationship between eBMD and CVD to identify potential new pathways, to estimate causality, to investigate direction of the estimated causality, and to address potential pleiotropy. The authors have used current technology, current software and current statistical methods to address these questions. The article is precise and concise, well presented, and the authors justify their arguments.

 Could the authors please comment on why this study was relevant to undertake in text, discussion, and if space allows also in the abstract? Which potential clinical implications could this have? Does the study open up for any potential drug targets? What would be the equivalent target randomized trial that this MR study mimicks?

I have read this submission. I believe that I have an appropriate level of expertise to confirm that it is of an acceptable scientific standard.

# Peer review: Referee response for version 1

**DOI:** 10.21956/wellcomeopenres.13302.r25235

**Published:** 2017-09-01

**Authors:** J. Brent Richards

**Affiliations:** 1 Department of Human Genetics, Lady Davis Institute for Medical Research, Jewish General Hospital, McGill University, Montreal, QC, Canada

Gan *et al. *have undertaken a two-sample MR study using 235 independent SNPs for eBMD in UKbiobank and tested them for T2D and CHD risk in DIAGRAM and CARDIOGRAMplusC4D. They found a small increase in T2D and CHD risk, whose confidence intervals overlapped, or nearly overlapped the null.

 This is a well-done study, addressing an important problem using modern Mendelian randomization techniques.

 Major Comments:

1. The authors have tested 12 traits and two diseases. They have made no attempt to correct for multiple testing. Given that the effects on T2D and CHD have p-values very near to 0.05, should they not make the reader aware that these results may have arisen by chance? I acknowledge that many of the traits are correlated, but given the number of hypothesis tested, it would be quite helpful to discuss the role that multiple testing may have had in their results.
2. A standard deviation change in BMD is a very large change. The resultant changes in risk of T2D and CHD were correspondingly small. In my opinion, it would be helpful to emphasize this to the reader in the abstract, the results and conclusions.
3. It would be helpful to know how population stratification was handled in the GWAS for eBMD.
4. The authors should state that MR-Egger tests for unbalanced horizontal pleiotropy, rather than “pleiotropy”.
5. It is not clear how the genetic associations with the 12 established metabolic risk factors were identified. It appears they were mostly from UKB? If so, they would be biased towards the observational effect.
6. The authors should be applauded for bringing up the clinically relevant point that bisphosphonate use is not at all associated with an increased risk of CHD in studies available to date. Even if they were, the effects of these medications on BMD are far lower than one standard deviation and thus the effects on CHD risk would be anticipated to be even closer to the null than the results presented by the authors.

I have read this submission. I believe that I have an appropriate level of expertise to confirm that it is of an acceptable scientific standard, however I have significant reservations, as outlined above.
